# tRNA‐Derived Fragment *tRF‐22* Promotes Immunosuppression by Inhibiting HnRNPAB Ubiquitination in Esophageal Squamous Cell Carcinoma

**DOI:** 10.1002/advs.202505806

**Published:** 2025-10-27

**Authors:** Ling Pan, Xin Qin, Li Gong, Haining Liu, Bo Cheng, Jing Wang, Yajie Hu, Lingxing Zeng, Yiping Wang, Qingxu Song, Yufeng Cheng

**Affiliations:** ^1^ Department of Radiation Oncology Qilu Hospital of Shandong University Cheeloo College of Medicine Shandong University Jinan 250012 China; ^2^ Shandong Provincial Key Laboratory of Malignant Tumor Precision Treatment Jinan 250012 China; ^3^ Shandong Provincial Engineering Research Center for Tumor Precision Treatment Jinan 250012 China; ^4^ Department of Urology Qilu Hospital of Shandong University Cheeloo College of Medicine Shandong University Jinan 250012 China; ^5^ Department of Organ Transplantation Qilu Hospital of Shandong University Cheeloo College of Medicine Shandong University Jinan 250012 China; ^6^ Department of Urologic Oncology The First Affiliated Hospital of USTC Division of Life Sciences and Medicine University of Science and Technology of China Hefei 230001 China; ^7^ State Key Laboratory of Oncology in South China and Guangdong Provincial Clinical Research Center for Cancer Sun Yat‐sen University Cancer Center Guangzhou 510060 China

**Keywords:** esophageal squamous cell carcinoma, hnRNPAB, immunosuppressive tumor microenvironment, immunotherapy, tRF‐22

## Abstract

Small proportions of patients with esophageal squamous cell carcinoma (ESCC) benefit from immune checkpoint blockade therapy, making it urgent to identify factors that limit its effectiveness. It is found that *tRF‐22*, a small RNA derived from *tRNA^GlnCTG/TTG^*, promotes an immunosuppressive tumor microenvironment. High *tRF‐22* expression is associated with worse prognosis in ESCC. *tRF‐22* shapes immunosuppression by increasing polymorphonuclear myeloid‐derived suppressor cells (PMN‐MDSCs) infiltration and suppressing CD8^+^ T cells. Mechanistically, it binds to Lys91 on hnRNPAB, inhibiting its ubiquitination by TRIM25, leading to the stabilization of hnRNPAB, which activates *TGFB2* transcription. Accumulated TGFβ2 promotes MDSCs generation to drive immunosuppression in ESCC. Using *tRF‐22* antagomir or TGFβ signaling blockade in combination with anti‐PD1 therapy enhances immune response and reduces tumor growth. Overall, a *tRF‐22*–hnRNPAB–TGFβ2–PMN‐MDSCs–CD8^+^ T cell pathway is identified that drives immunosuppression and tumor growth. Targeting *tRF‐22* may be a promising strategy to improve immunotherapy efficacy in ESCC.

## Graphical abstract

*tRF‐22* fosters an immunosuppressive and pro‐oncogenic tumor microenvironment in ESCC by stabilizing hnRNPAB against TRIM25‐mediated ubiquitination and enhancing TGFβ2 expression, which increases PMN‐MDSCs infiltration and suppresses CD8^+^ T cells. Targeting *tRF‐22* or blocking TGFβ signalling improves anti‐PD1 response, offering a promising therapeutic strategy to enhance immunotherapy efficacy in ESCC.

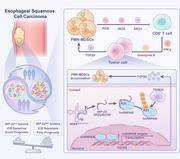

## 1 Introduction

Over the past decades, immune checkpoint blockade (ICB) has shown promising clinical benefits across various cancers by reversing the exhaustion of tumor‐infiltrating lymphocytes.^[^ ^1^, ^2^ ^]^ However, a significant proportion of cancer patients remain non‐responsive or resistant to ICB.^[^ ^3^ ^]^ Esophageal squamous cell carcinoma (ESCC), accounting for over 80% of all esophageal cancer cases, is a leading cause of cancer‐related deaths, particularly in East Asia.^[^ ^4^, ^5^ ^]^ ESCC exhibits a relatively high tumor mutation burden, highlighting the molecular foundation for the efficacy of ICB therapy.^[^ ^6^, ^7^ ^]^ Recent clinical trials demonstrate that combining PD‐1 inhibitors with chemotherapy provides better outcomes than chemotherapy alone, but this approach remains only partially effective.^[^ ^8^, ^9^, ^10^ ^]^ A major challenge in the ICB treatment is the immunosuppressive tumor microenvironment (TME). The complex interplay between the tumor and its surrounding microenvironment poses considerable obstacles to effective immunotherapy in ESCC.^[^ ^11^ ^]^

Myeloid‐derived suppressor cells (MDSCs), key players in the immunosuppressive TME, are classified into two main subtypes: monocytic MDSCs (M‐MDSCs) and polymorphonuclear MDSCs (PMN‐MDSCs). Both subsets inhibit T cell activation and proliferation but exert their effects through distinct mechanisms. PMN‐MDSCs primarily mediate immune suppression via reactive oxygen species (ROS), peroxynitrite, arginase‐1, and prostaglandin E2 (PGE2). In contrast, M‐MDSCs utilize nitric oxide (NO), cytokines such as IL‐10 and TGF‐β, and immune checkpoint molecules like PD‐L1. Metabolically, PMN‐MDSCs enhance fatty acid oxidation through CD36 and FATP2 and increase glycolysis to survive in the TME. M‐MDSCs, however, exhibit a metabolically dormant phenotype characterized by reduced glucose metabolism and ATP production, leading to methylglyoxal accumulation that impairs T cell function via protein glycation and amino acid depletion.^[^ ^12^, ^13^, ^14^, ^15^, ^16^ ^]^ The generation of MDSCs from myeloid progenitors in bone marrow and peripheral blood mononuclear cells (PBMCs) is a complex and gradual process regulated by multiple factors, including GM‐CSF, IL‐6, and TGFβ.^[^ ^17^, ^18^, ^19^ ^]^ Notably, TGFβ has been demonstrated to be a potent enhancer, synergistically working with IL‐6 and GM‐CSF to promote the induction of MDSCs.^[^ ^20^, ^21^, ^22^, ^23^ ^]^ MDSCs are significantly enriched in ESCC, and their high infiltration is associated with poor prognosis and immunotherapy efficacy in ESCC patients.^[^ ^11^ ^]^ Identifying the factors that drive MDSCs expansion in ESCC could help overcome the immunosuppressive barriers in the TME and enhance the overall therapeutic outcomes for these patients.

Transfer RNA‐derived fragments (tRFs), a novel class of small non‐coding RNAs derived from mature or precursor tRNAs, are classified into distinct types, including 5′ tRFs, 3′ tRFs, and i‐tRFs. These fragments have gained significant attention in cancer research^[^ ^24^, ^25^ ^]^ due to their roles in gene silencing,^[^ ^26^, ^27^ ^]^ ribosome genesis,^[^ ^28^ ^]^ translation efficiency^[^ ^29^ ^]^ and RBP‐dependent regulation.^[^ ^30^, ^31^ ^]^ Recently, their role in immune regulation has garnered significant attention. T cells release specific immunosuppressive tRFs through extracellular vesicles, boosting interleukin‐2 (IL‐2) production and T cell activation.^[^ ^32^ ^]^ Additionally, the translation of IL‐2 receptors is inhibited by tRFs, halting T cell proliferation.^[^ ^33^ ^]^ Although the roles of tRFs in immune regulation have been preliminarily reported, their impacts on the immune system and the crosstalk between tumor cells and TME remain largely unexplored, particularly in the context of ESCC.

Here, we identified a novel RNA fragment, *tRF‐22‐DRFU8U76F* (*tRF‐22*), which is associated with the survival of patients with ESCC based on deep mining of The Cancer Genome Atlas (TCGA) small RNA‐seq data. *tRF‐22* promotes cancer progression by suppressing the immune system rather than directly affecting tumor cell malignant behavior. We demonstrated that *tRF‐22* binds to heterogeneous nuclear ribonucleoprotein A/B (hnRNPAB) and prevents its ubiquitination by tripartite motif‐containing protein 25 (TRIM25), increasing the expression of hnRNPAB, which enhances the production of TGFβ2, an important factor in the generation of immunosuppressive PMN‐MDSCs. The resulting increase in PMN‐MDSCs and decrease in CD8^+^ T cell number enable tumor cells to evade the immune system. Consequently, ESCC patients with higher *tRF‐22* levels have poorer responses to immunotherapy and shorter progression‐free survival (PFS). Targeting *tRF‐22* and TGFβ2 could significantly improve the effectiveness of PD‐1 blockade. These findings suggest that *tRF‐22* could serve as a promising therapeutic target in ESCC treatment, potentially enhancing the efficacy of current immunotherapies.

## 2 Results

### 2.1 Prognosis‐Associated *tRF‐22* Drives ESCC Progression via Immune Modulation

**Figure 1:**
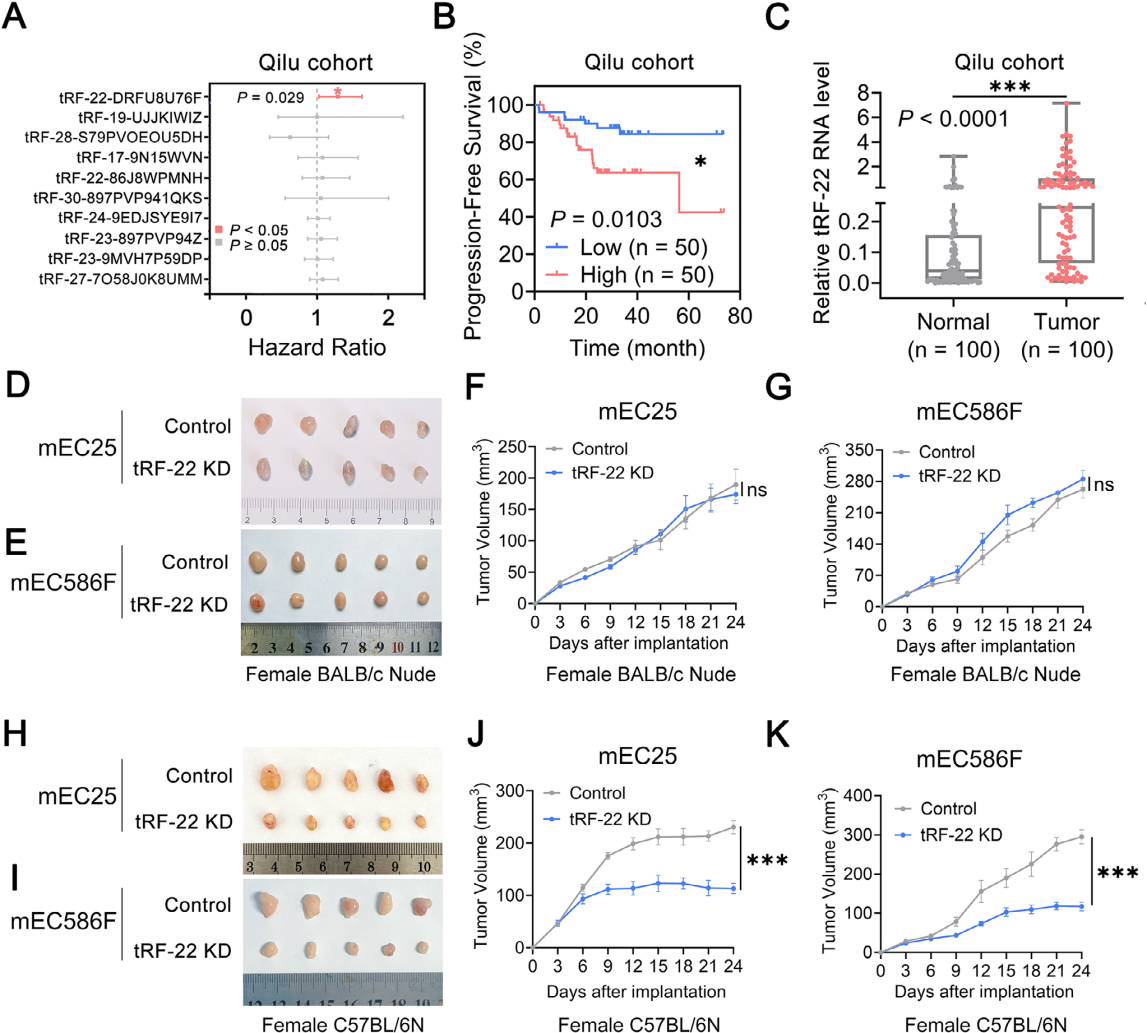
*tRF‐22* is associated with survival time in patients with ESCC and drives tumor progression via immune modulation. A) Associations between the expression levels of ten tRFs and patients’ overall survival time in the Qilu hospital cohort (*n* = 100), showing a significant association only for tRF‐22. ^*^, *p* < 0.05. B) Kaplan‐Meier estimates of patients’ progression‐free survival time in the Qilu hospital cohort according to *tRF‐22* levels in ESCC. *P* values were determined by the log‐rank test. ^*^, *p* < 0.05. C) Relative *tRF‐22* levels in ESCC tissues and paired normal tissues from Qilu hospital cohort (*n* = 100) by RT‐qPCR. ^***^, *p* < 0.001 by t‐test. D–K) Effects of *tRF‐22* expression changes on tumor burden in female BALB/c nude mice (D–G) and female C57BL/6N mice H–K) with subcutaneous mEC25 and mEC586F cell transplantation (*n* = 5 per group). F, G, D, K) Data presented as mean ± SEM. ^***^, *p* < 0.001, and ns, not significant by t‐test.

We investigated the association between tRFs and survival time in 94 patients from the TCGA ESCC cohort. Univariate Cox regression analysis identified 10 out of 714 tRFs in the short RNA data that were significantly associated with overall survival (Figure S1A,B, Supporting Information). To validate these findings, we analyzed ten tRFs in the Qilu hospital cohort. Among these, only *tRF‐22‐DRFU8U76F* (*tRF‐22*) levels were significantly correlated with patients’ overall survival (**Figure** **1**A). Survival analysis further revealed that ESCC patients with elevated *tRF‐22* levels had significantly shorter overall and progression‐free survival times (Figure 1B; Figure S1C, Supporting Information). We also assessed the expression levels of *tRF‐22* in patients with ESCC and found that they were significantly higher in tumor regions compared to paired normal tissues (Figure 1C) and significantly higher in advanced tumor stages (stages III/IV) than in early tumor stages (stages I/II) (Figure S1D, Supporting Information). The median levels of *tRF‐22* tend to be higher in tumor tissues than in adjacent normal tissues, likely due to limited statistical power from only three normal samples in the TCGA ESCC cohort. Notably, *tRF‐22* expression is significantly elevated in advanced tumor stages (III/IV) compared to early stages (I/II) (Figure S1E,F, Supporting Information). Additionally, serum *tRF‐22* expression is significantly higher in ESCC patients than in healthy individuals and higher in advanced tumor stages (III/IV) than in early tumor stages (I/II) (Figure S1G,H, Supporting Information). Despite the higher incidence of esophageal cancer in males, no significant sex‐based differences in *tRF‐22* expression were observed in either the Qilu or TCGA cohort (Figure S1I,J, Supporting Information). Additionally, no significant correlations between *tRF‐22* expression and treatment regimens were found in either cohort (Figure S1K,L, Supporting Information). All these results suggest that *tRF‐22* is involved in ESCC progression.

We further confirmed that *tRF‐22* is derived from mature *tRNA^GlnCTG/TTG^* ^[^ ^34^ ^]^ and could be exactly amplified using stem‐loop primers (Figure S2A,B, Supporting Information). Northern blot analysis confirmed the existence of *tRF‐22* in human ESCC cells (Figure S2C, Supporting Information). Fluorescence in situ hybridization and cell fractionation analyses revealed that *tRF‐22* is predominantly located in the cytoplasm (Figure S2D,E, Supporting Information). We next examined the effects of *tRF‐22* on malignant cell phenotypes by establishing human ESCC cell lines with *tRF‐22* stably overexpressed or silenced (Figure S3A, Supporting Information). The results showed that, although changing *tRF‐22* levels in ESCC cells did not alter the cellular mature *tRNA^GlnCTG/TTG^* levels, *tRF‐22* had no obvious effects on cell proliferation, migration, or invasion in vitro (Figure S3A–F, Supporting Information). Similarly, *tRF‐22* did not notably affect tumor growth in nude mice xenograft assays (Figure S3G–I, Supporting Information). Given the significant prognostic value of *tRF‐22*, we hypothesized that it may play a crucial role in the TME regulation. Given the high conservation of *tRF‐22* between human and mouse (Table S1, Supporting Information), we established a mouse mEC25 cell line with stable *tRF‐22* knockdown or applied antagotRF‐22 treatment. Northern blot and tsRNA/tRNA‐seq analyses both confirmed specific reduction of *tRF‐22* levels without altering the abundance of its parental *tRNA^GlnCTG/TTG^* (Figures S3A and S4A–C, Supporting Information). In female BALB/c nude mice, neither *tRF‐22* knockdown in mEC25 cells nor mEC586F cells (derived from female mice) nor antagotRF‐22 treatment significantly affected tumor growth compared to controls (Figure 1D–G; Figure S5A–D, Supporting Information). In contrast, *tRF‐22* depletion in female C57BL/6N mice resulted in a pronounced tumor‐suppressive effect (Figure 1H–K; Figure S5E–H, Supporting Information). To further control for potential sex‐related confounding effects, we separately inoculated *tRF‐22*–silenced mEC25 and mEC525M (derived from male mice) cell lines into male nude mice and C57BL/6N mice. The results were consistent with those observed in the female model, where *tRF‐22* deletion did not suppress tumor growth in nude mice but significantly inhibited tumor progression in C57BL/6N mice (Figure S5I–P, Supporting Information). Consistently, KEGG enrichment analysis revealed that genes that were significantly correlated with *tRF‐22* expression based on TCGA ESCC data were notably enriched in numerous immune signaling pathways (Figure S6A, Supporting Information). Collectively, all these findings suggest that *tRF‐22* may promote ESCC progression by modulating the immune microenvironment in a sex‐independent manner.

### 2.2 *tRF‐22* Fosters an Immunosuppressive and Pro‐Oncogenic Tumor Microenvironment via PMN‐MDSCs

**Figure 2:**
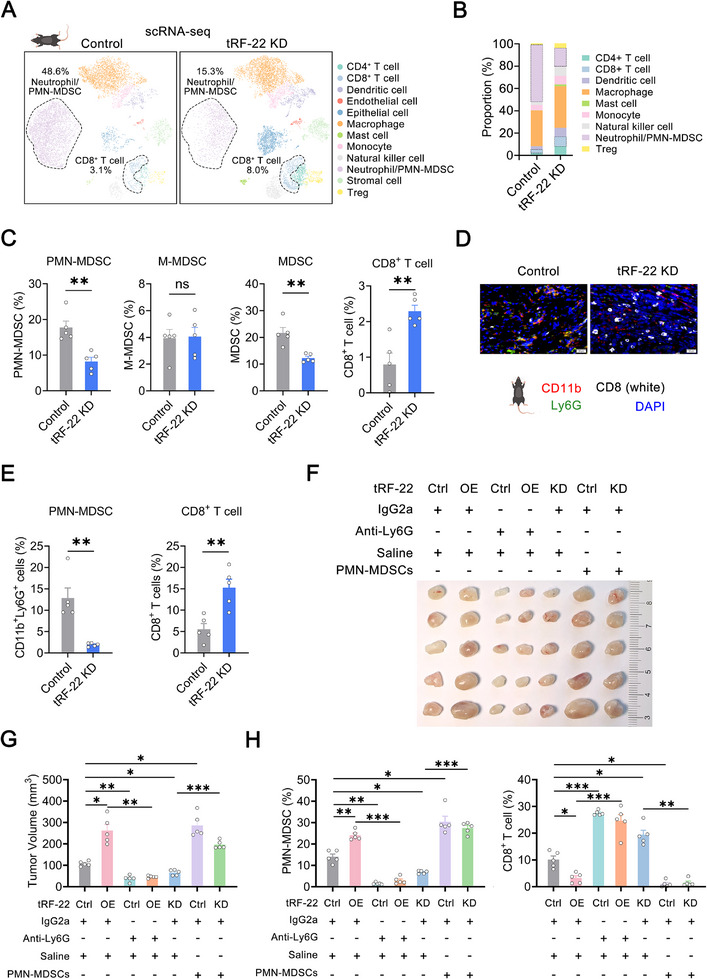
*tRF‐22* enhances ESCC progression via PMN‐MDSCs. A, B) UMAP plots (A) and bar plots (B) for different proportions of cell subclusters. C) The proportions of CD11b^+^Gr‐1^+^ MDSCs, CD11b^+^Gr‐1^+^Ly6G^+^Ly6C^low^ PMN‐MDSCs, CD11b^+^Gr‐1^+^Ly6G^−^Ly6C^high^ M‐MDSCs and CD8^+^ T cells in tumors by flow cytometry (*n* = 5). Data presented as mean ± SEM. ^**^, *p* < 0.01 and ns, not significant by t‐test. D, E) The abundance of CD11b^+^Ly6G^+^ PMN‐MDSCs and CD8^+^ T cells by mIHC. Representative mIHC staining images (D) and quantification (*n* = 5) (E). Data presented as mean ± SEM. ^**^, *p* < 0.01 by t‐test. Scale bars, 20 µm. F, G) Image (F) and quantification (G) of tumors receiving the indicated treatments (*n* = 5 per group). H) The proportions of CD11b^+^Gr‐1^+^Ly6G^+^Ly6C^low^ PMN‐MDSCs and CD3^+^CD8^+^ T cells in these tumors were determined by flow cytometry (*n* = 5 per group). (G, H) Data presented the mean ± SEM. ^*^, *p* < 0.05, ^**^, *p* < 0.01, ^***^, *p* < 0.001 by Brown‐Forsythe ANOVA with Dunnett's T3 multiple comparison test.

To further characterize the immunological landscape of the TME, we performed single‐cell RNA sequencing (scRNA‐seq) on fresh tissue samples derived from *tRF‐22* knockdown and control mEC25 tumors. Based on the expression of known lineage marker genes, a total of 15 400 cells from two samples were classified into 12 distinct cell types, including neutrophil/PMN‐MDSC, macrophage, dendritic cell, CD8^+^ T cell, CD4^+^ T cell, natural killer cell, Treg cell, and additional types (**Figure** **2**A; Figure S6B–D, Supporting Information). While all cell clusters were present in both groups, their proportions varied. The proportion of neutrophil/PMN‐MDSC was dramatically reduced in the *tRF‐22* knockdown group, whereas anti‐tumor immune cells, including CD8^+^ T cells, CD4^+^ T cells, DC, and NK cells, increased (Figure 2B). We then validated the scRNA‐seq results using flow cytometry to detect various immune cell types in these two tumor samples, and the results indicated a decrease in CD11b^+^Gr‐1^+^ cells (MDSCs), especially Ly6G^+^Ly6C^low^ cells (PMN‐MDSCs) within this population in the *tRF‐22* knockdown group. Conversely, the proportion of Ly6G^−^Ly6C^high^ cells (M‐MDSCs) was similar between the two groups (Figure 2C; Figure S6E,F, Supporting Information). Distinguishing PMN‐MDSCs from neutrophils solely by gene expression features is challenging. Therefore, we further isolated CD11b^+^Gr‐1^+^Ly6G^+^Ly6C^low^ cells from fresh tumors and co‐cultured them with CFSE‐labeled splenic CD8^+^ T cells from non‐tumor‐bearing mice. The dose‐dependent suppression of CD8^+^ T cells' proliferation and reduction of IFN‐γ secretion confirmed that these cells were indeed PMN‐MDSCs (Figure S7A–C, Supporting Information). Consistent with the immunosuppressive role of PMN‐MDSCs, the proportion of CD8^+^ T cells in tumor tissues significantly increased after *tRF‐22* knockdown (Figure 2C; Figure S6F, Supporting Information). Other immune cell populations, such as macrophages, DC, NK cells, and CD4^+^ T cells, did not show significant changes (Figure S6G, Supporting Information). Multiple Immunohistochemistry (mIHC) assays for CD11b, Ly6G, and CD8 confirmed the results of flow cytometry, showing decreased CD11b^+^Ly6G^+^ PMN‐MDSCs infiltration and increased CD8^+^ T cells infiltration in the *tRF‐22* knockdown group (Figure 2D,E). Correspondingly, antagotRF‐22 treatment suppressed tumor growth, reduced PMN‐MDSCs levels, and enhanced CD8^+^ T cell infiltration (Figures S5E–H and S7D–F, Supporting Information). Based on the above findings, we inferred that *tRF‐22* might reduce CD8^+^ T cell numbers and suppress its function by promoting the accumulation of PMN‐MDSCs.

For validation, rescue experiments were performed. We administered an anti‐Ly6G antibody to mice with tumors overexpressing *tRF‐22* and investigated the roles of PMN‐MDSCs in *tRF‐22*‐driven ESCC progression (Figure S8A, Supporting Information). PMN‐MDSCs deletion reversed the reduction of CD8^+^ T cells and the tumor growth caused by *tRF‐22* overexpression. In contrast, adoptive transfer of PMN‐MDSCs reversed the increase of CD8^+^ T cells and the tumor shrinkage resulting from *tRF‐22* silencing (Figure 2F–H; Figure S8B, Supporting Information). These observations were further confirmed by mIHC (Figure S8C,D, Supporting Information). Taken together, these findings further support that PMN‐MDSCs are key mediators of *tRF‐22*–driven ESCC progression.

### 2.3 *tRF‐22* Binds to hnRNPAB, Preventing Its Degradation and Promoting PMN‐MDSCs Accumulation

**Figure 3:**
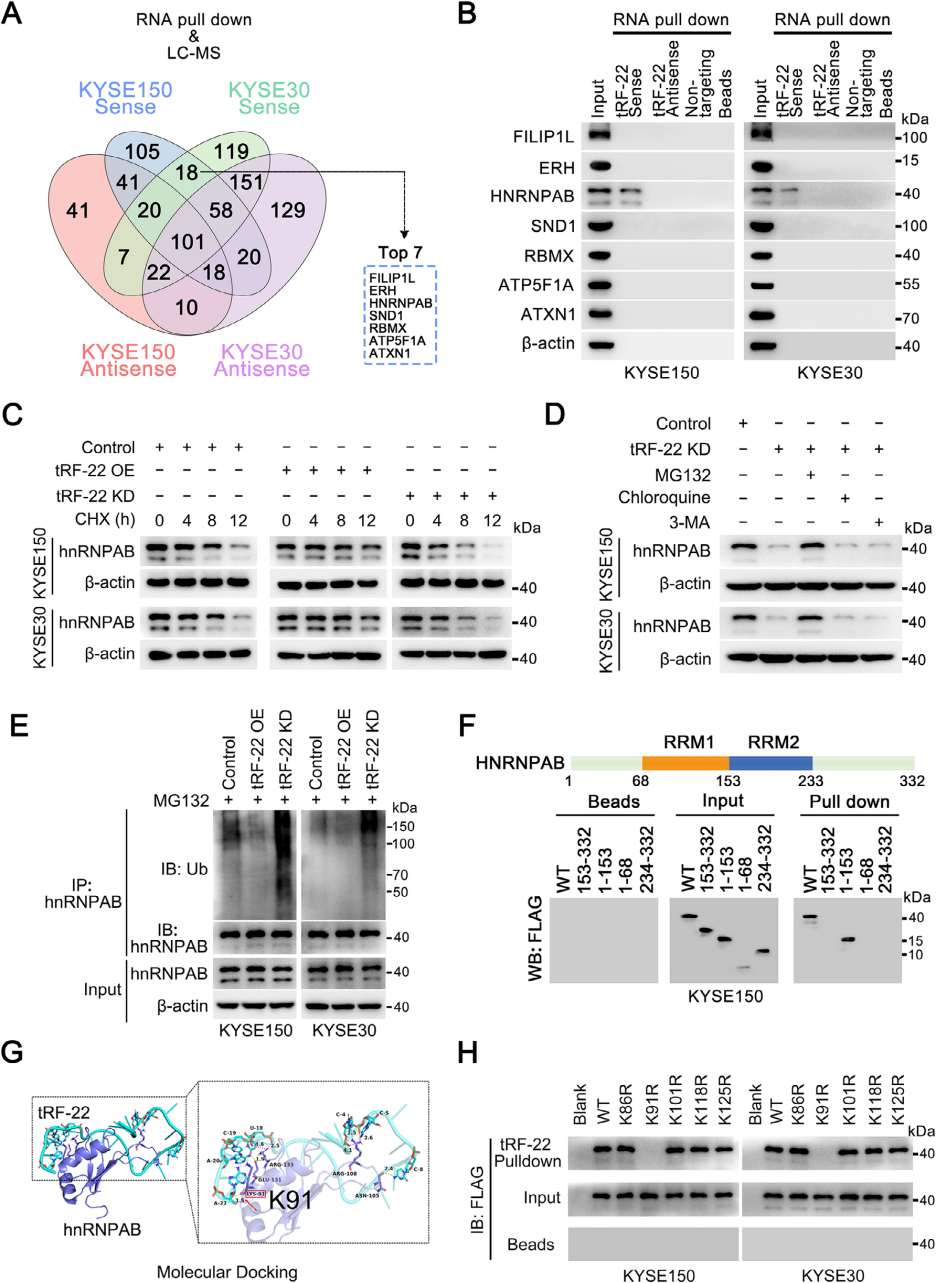
*tRF‐22* interacts with hnRNPAB at Lys91. A) Schematic of the RNA‐pulldown assays followed by liquid chromatography‐mass spectrometry (LC‐MS) using a *tRF‐22* sense or antisense probe for the identification of proteins that specifically bind *tRF‐22*. B) Representative immunoblot of products from RNA‐pulldown assays using *tRF‐22* sense, *tRF‐22* antisense, or a non‐targeting oligo suggested 7 potential *tRF‐22*–binding proteins. C) ESCC cells were transfected with the indicated plasmids, followed by cycloheximide (CHX, 10 µg mL^−1^) treatment for the indicated duration. Representative immunoblot of hnRNPAB and β‐actin after the above treatment. D) Following transfection with the indicated plasmids and treatment with 3‐MA, chloroquine, or MG132 for 4 h, cell lysates were subjected to western blot analyses, and the representative immunoblots are shown. E) ESCC cells were co‐transfected with the indicated plasmids for 48 h, and treatment with MG132 for 4 h Cell lysates were then subjected to IP using anti‐hnRNPAB antibody, followed by western blot analyses, and the representative immunoblots are shown. F) Truncation mapping of the *tRF‐22*–hnRNPAB binding domains. The schematic diagram shows the FLAG‐tagged hnRNPAB protein domain structure (*upper panel*). Representative immunoblots show FLAG‐tagged wild‐type hnRNPAB and its truncated forms pulled down by *tRF‐22* (*lower panel*). G) Molecular docking model simulates the binding site between *tRF‐22* and hnRNPAB. H) Representative immunoblots show FLAG‐tagged wild‐type hnRNPAB (WT) and its mutated forms (K86R, K91R, K101R, K118R, and K125R) retrieved by *tRF‐22*. All experiments were performed at least three times.

To investigate how *tRF‐22* promotes PMN‐MDSCs accumulation, we conducted RNA pulldown assays using biotinylated *tRF‐22* and its antisense oligonucleotide with ESCC cell lysates, followed by mass spectrometry analyses. We identified 18 proteins that specifically interact with *tRF‐22* but not its antisense RNA. Then we focused on the top 7 most abundant proteins for further validation (**Figure** **3**A; Table S2, Supporting Information) and found that only hnRNPAB, a key transcriptional regulator, interacted with *tRF‐22* (Figure 3B). RNA immunoprecipitation (RIP) assays with the hnRNPAB antibody further validated this specific interaction (Figure S9A, Supporting Information). The mRNA levels of *HNRNPAB* remained unchanged when *tRF‐22* levels were altered, but the protein levels of hnRNPAB increased with *tRF‐22* overexpression and decreased with *tRF‐22* knockdown (Figure S9B, Supporting Information). This suggests that *tRF‐22* may influence hnRNPAB protein stability. Further experiments showed that *tRF‐22* overexpression extended the half‐life of hnRNPAB, while knockdown of *tRF‐22* shortened it (Figure 3C; Figure S9C, Supporting Information). The proteasome inhibitor MG132 prevented the degradation of hnRNPAB caused by *tRF‐22* knockdown, whereas inhibitors of lysosomal and the autophagy pathway had no effect, indicating that *tRF‐22* stabilizes hnRNPAB by protecting it from proteasomal degradation (Figure 3D). Consistently, *tRF‐22* overexpression reduced hnRNPAB ubiquitination, while *tRF‐22* knockdown increased it (Figure 3E). Protein domain mapping assays revealed that the RRM1 domain of hnRNPAB is essential for its interaction with *tRF‐22* (Figure 3F). Using the Ubibrowser^2.0^ database, we identified five candidate ubiquitination sites within the RRM1 domain of hnRNPAB and predicted that they are highly conserved across different species (Figure S9D, Supporting Information). Among them, Lys91 is proposed to be the key residue for *tRF‐22* binding, according to molecular docking studies (Figure 3G). RNA pulldown assays with ESCC cells expressing FLAG‐tagged hnRNPAB mutants revealed that *tRF‐22* could not bind to the Lys91Arg mutant, while other mutations had no such effect (Figure 3H). RIP assays further confirmed that Lys91 is the crucial binding site for *tRF‐22* on hnRNPAB (Figure S9E, Supporting Information). In conclusion, *tRF‐22* promotes hnRNPAB stability through blocking Lys91 ubiquitination; however, there has been no direct evidence for the role of hnRNPAB in PMN‐MDSCs expansion (Figure S9F, Supporting Information). As the in vivo results in Figure 5F–I, *HNRNPAB* silencing reversed the increase of PMN‐MDSCs and the reduction of CD8^+^ T cells, and recovered the tumor growth caused by *tRF‐22* overexpression. All these findings indicate that *tRF‐22* promotes PMN‐MDSCs accumulation by enhancing the protein stability of hnRNPAB.

### 2.4 *tRF‐22* Prevents TRIM25 from Ubiquitinating hnRNPAB

**Figure 4:**
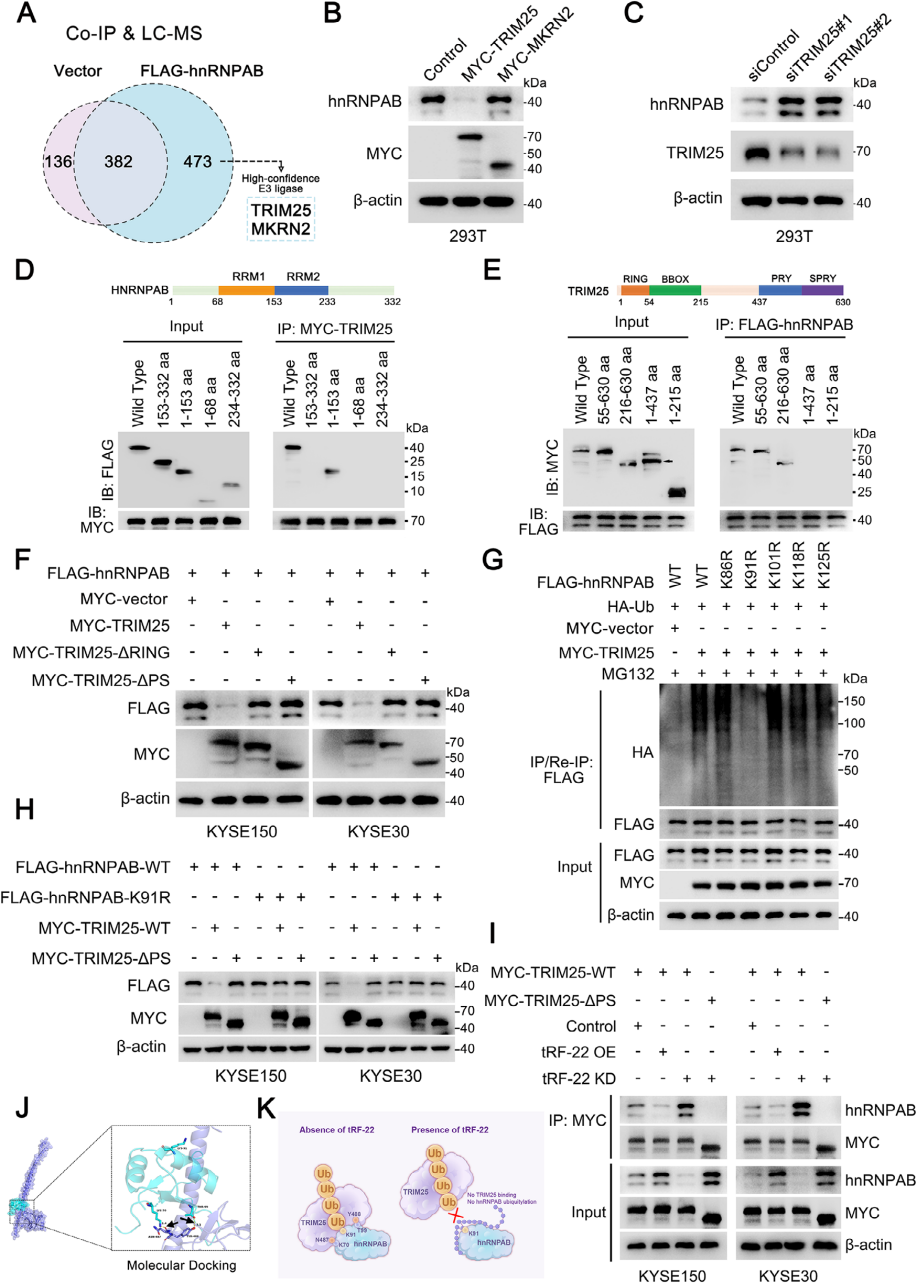
*tRF‐22* suppresses the ubiquitination of hnRNPAB at Lys91 by TRIM25. A) Lysates from 293T cells were IP with anti‐FLAG antibody, and the products were sequenced by LC‐MS analyses to identify the proteins that specifically bind hnRNPAB. B,C) 293T cells transfected with the indicated plasmids or siRNAs for 48 h were analyzed by western blotting, and the representative immunoblots are shown. D,E) Truncation mapping of the interaction between hnRNPAB and TRIM25. Schematic diagrams show FLAG‐hnRNPAB and MYC‐TRIM25 protein domain structures, respectively (*upper panel*). Representative immunoblots showed the association of FLAG‐hnRNPAB constructs (full‐length vs their truncated forms) and MYC‐TRIM25 (D, *lower panel*) or MYC‐TRIM25 constructs (full‐length vs their truncated forms) and FLAG‐hnRNPAB (E, *lower panel*). Arrow indicates MYC‐TRIM25‐ΔPS construct. F) ESCC cells with the indicated plasmids were analyzed by immunoblotting analyses with the indicated antibodies. The representative immunoblots are shown. G) IP analyses were performed for lysates prepared from 293T cells following HA‐Ub, MYC‐TRIM25/vector, and FLAG‐hnRNPAB (WT or point mutants) plasmids co‐transfection, and the representative immunoblots are shown. H) Immunoblotting analyses were performed for lysates prepared from 293T cells following MYC‐TRIM25‐WT/∆PS and FLAG‐hnRNPAB‐WT/ K91R plasmids co‐transfection, and the representative immunoblots are shown. I) Immunoprecipitation assays and subsequent immunoblotting analyses were performed for lysates prepared from ESCC cell lines following MYC‐TRIM25‐WT/∆PS and *tRF‐22* OE/KD plasmids co‐transfection, and the representative immunoblots are shown. J) Molecular docking model simulates the binding sites between hnRNPAB and TRIM25. K) Schematic of *tRF‐22* in the binding of TRIM25 with hnRNPAB. All experiments were performed at least three times.

To identify the E3 ubiquitin ligase for hnRNPAB and understand how *tRF‐22* regulates its ubiquitination, we performed immunoprecipitation following mass spectrometry using 293T cells expressing FLAG‐tagged hnRNPAB (Figure S10A, Supporting Information). Two candidate E3 ligases, TRIM25 and MKRN2, were identified among the hnRNPAB‐interacting proteins (**Figure** **4**A; Table S3, Supporting Information). However, only TRIM25 was shown to reduce hnRNPAB levels (Figure 4B). When TRIM25 was silenced, hnRNPAB levels increased, confirming its role in regulating hnRNPAB expression (Figure 4C). The interaction between hnRNPAB and TRIM25 was validated by co‐immunoprecipitation assays (Figure S10B,C, Supporting Information). Deletion of the PRY‐SPRY (PS) domain of TRIM25 prevented its binding to hnRNPAB, while deletion of the RRM1 domain in hnRNPAB prevented its binding to TRIM25, suggesting that the PS domain of TRIM25 and the RRM1 domain of hnRNPAB are crucial for their interaction (Figure 4D,E). TRIM25 has been reported to exert the E3 ubiquitin ligase activity depending on its RING domain.^[^ ^35^, ^36^ ^]^ When we overexpressed TRIM25 in ESCC cells, hnRNPAB expression decreased with wild‐type TRIM25, but not with TRIM25 mutants lacking the RING or PS domain (Figure 4F). This decrease was abrogated by MG132 treatment (Figure S10D, Supporting Information), confirming that the RING domain is responsible for E3 ubiquitin ligase activity and the PS domain is essential for TRIM25 binding to hnRNPAB.

Further experiments showed that wild‐type TRIM25, but not its ΔRING or ΔPS mutant, decreased hnRNPAB protein levels, enhanced its ubiquitination, and shortened its half‐life (Figure S10E–G, Supporting Information). Conversely, TRIM25 knockdown reduced hnRNPAB ubiquitination and extended its half‐life (Figure S10H–J, Supporting Information). Mutation analysis of the five Lys (K) residues in hnRNPAB showed that only the hnRNPAB Lys91Arg mutant was highly resistant to ubiquitination (Figure 4G), suggesting that Lys91 is the key site for TRIM25‐mediated ubiquitination. The Lys91Arg mutant also had a significantly longer half‐life than wild‐type hnRNPAB in ESCC cells (Figure S11A–C, Supporting Information). Only when both TRIM25 and hnRNPAB were wild‐type, hnRNPAB could be ubiquitinated and degraded. When either the hnRNPAB Lys91Arg mutant or the TRIM25 ΔPS mutant was present, hnRNPAB degradation did not occur (Figure 4H). This highlights the critical role of the PS domain of TRIM25 in binding to the RRM1 domain of hnRNPAB and promoting its ubiquitination at Lys91 residue.

The role of *tRF‐22* in this process is particularly critical. Co‐immunoprecipitation assays showed that increasing *tRF‐22* levels significantly hindered the interaction between hnRNPAB and TRIM25, while decreasing *tRF‐22* enhanced it. This effect was not observed with the TRIM25 ΔPS mutant, indicating that *tRF‐22* specifically modulates the interaction between hnRNPAB and the PS domain of TRIM25 (Figure 4I). Rescue experiments further demonstrated that *tRF‐22* overexpression could restore TRIM25‐mediated degradation of hnRNPAB (Figure S11D, Supporting Information). Compared with wild‐type hnRNPAB, the Lys91Arg mutant immunoprecipitated less *tRF‐22* by RIP assays, despite increased TRIM25 binding (Figure S11E, Supporting Information). Protein‐protein molecular docking model suggested that residues Asn487 and Tyr488 of the TRIM25 PS domain interact directly with the residues Lys70 and Thr99 of the hnRNPAB RRM1 domain, facilitating the ubiquitination of TRIM25 toward residue Lys91 of hnRNPAB (Figure 4J,K). Furthermore, in vivo validation confirmed the consistency of these findings with our in vitro observations (Figure S12A–D, Supporting Information). Altogether, these findings highlight the essential role of residue Lys91 in hnRNPAB for *tRF‐22* binding and subsequent TRIM25‐mediated proteasomal degradation.

### 2.5 *tRF‐22* and hnRNPAB Enhance PMN‐MDSCs Generation via TGFβ2 Induction

**Figure 5:**
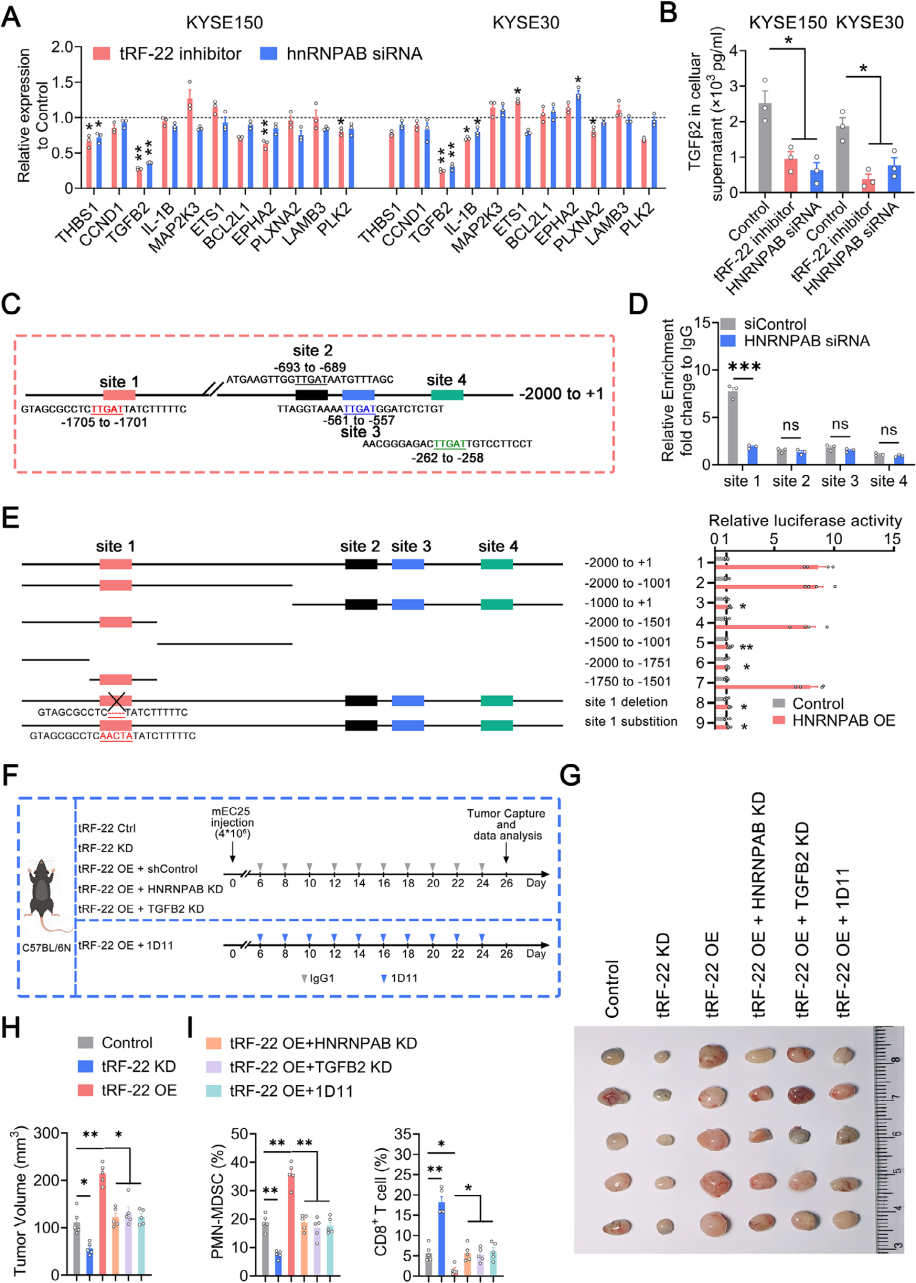
PMN‐MDSCs accumulation is regulated by *tRF‐22* and hnRNPAB‐mediated transcription activation of *TGFB2. A*) The mRNA levels of differentially expressed genes from RNA‐seq were detected by RT‐qPCR in ESCC cells with the silencing of *tRF‐22* or *HNRNPAB*. Data represent enrichment relative to control (*n* = 3). B) ELISA analysis of TGFβ2 in the cellular supernatant of ESCC cells with silence of *tRF‐22* or *HNRNPAB* (*n* = 3). The control group in (A, B) was co‐transfected with a negative control for *tRF‐22* and siControl for *HNRNPAB*. C) Schematic diagram of the predicted hnRNPAB‐binding four core pentanucleotide motifs TTGAT within the promoter region of *TGFB2*. D) CUT&RUN‐qPCR analyses showing the interaction of the promoter region (s) of *TGFB2* with hnRNPAB in KYSE150 cells treated with either siControl or *HNRNPAB* siRNA (*n* = 3). E) Deletion analysis and selective mutagenesis identified HNRNPAB‐responsive site (s) in the *TGFB2* promoter (*left panel*), along with the relative luciferase activity measured (*right panel*). F) Schematic diagram of *tRF‐22* overexpression or silence, combined with *HNRNPAB* or *TGFB2* silence, or administration of anti‐TGFβ antibody in ESCC mouse models. G, H) Image (G) and quantification (H) of tumors receiving treatments indicated above (*n* = 5 per group). I) The proportions of CD11b^+^Gr‐1^+^Ly6G^+^ PMN‐MDSCs and CD8^+^ T cells in these tumors were determined by flow cytometry (*n* = 5 per group). (A, B, E, H, I) Data presented as mean ± SEM. ^*^, *p* < 0.05; ^**^, *p* < 0.01 by Brown‐Forsythe ANOVA with Dunnett's T3 multiple comparison test. (D) Data presented as mean ± SEM. ^***^, *p* < 0.001 and ns, not significant by t‐test. All experiments were performed at least three times.

Evidence indicates that hnRNPAB is engaged in regulating key biological processes, including gene transcription^[^ ^37^, ^38^, ^39^ ^]^ and RNA splicing.^[^ ^40^ ^]^ Analysis of TCGA ESCC RNA‐seq data revealed that genes positively correlated with *HNRNPAB* expression are enriched in RNA polymerase and spliceosome pathways (Figure S13A, Supporting Information). Given the prominent transcriptional activity of hnRNPAB, we carried out RNA‐seq from KYSE150 cells with *tRF‐22* or *HNRNPAB* silenced (Figure S13B, Supporting Information). In the *tRF‐22* and *HNRNPAB* silenced groups, we identified 2148 and 3079 differentially expressed genes, respectively. Among these, 1459 genes were co‐regulated by both factors and showed significant enrichment in key cellular pathways, including the MAPK and TGF‐beta signaling pathways (Figure S13B,C, Supporting Information). We then silenced *tRF‐22* and *HNRNPAB* in ESCC cells to assess their effects on the significantly downregulated genes. *TGFB2* expression was notably downregulated by silencing both *tRF‐22* and *HNRNPAB* in two ESCC cell lines (**Figure** **5**A). Further enzyme‐linked immunosorbent assay (ELISA) confirmed that silencing these factors reduced TGFβ2 protein levels in cell supernatants (Figure 5B). It has been reported that hnRNPAB shows a potent ability to bind DNA at the canonical FTS‐1 motif, which contains the core pentanucleotide sequence TTGAT.^[^ ^37^, ^38^ ^]^ Sequence analysis of the *TGFB2* promoter identified four potential hnRNPAB binding sites (Figure 5C). Using CUT&RUN‐qPCR, we observed a significant reduction in hnRNPAB binding to site 1 of the *TGFB2* promoter in the hnRNPAB‐silenced group (Figure 5D). Luciferase reporter assays indicated that the region containing site 1 (−1705 to −1701 bp) is crucial for hnRNPAB‐induced *TGFB2* promoter activity (Figure 5E). These findings suggest that hnRNPAB is a critical regulator of *TGFB2* expression, with site 1 playing a key role in this transcriptional regulation.

We next measured TGFβ2 levels in peripheral blood and tumor interstitial fluid from mEC25 tumor‐bearing C57BL/6N mice. TGFβ2 secretion significantly decreased in the *tRF‐22*‐silenced group (Figure S13D,E, Supporting Information). Given the role of TGFβ in MDSCs generation, we employed an ex vivo culture system to examine the effects of TGFβ2 on the induction of CD11b^+^CD33^+^HLA‐DR^−^ early‐stage MDSCs.^[^ ^15^, ^20^, ^41^ ^]^ PBMCs were cultured with conditioned medium from KYSE150 cells with different *tRF‐22* levels. We found that *tRF‐22* silence decreased the number of MDSCs, while *tRF‐22* overexpression increased it. However, knockdown of *HNRNPAB* or *TGFB2*, or the addition of anti‐TGFβ antibody, reversed the *tRF‐22*‐induced MDSCs generation (Figure S13F,G, Supporting Information). In vivo, *tRF‐22* overexpression resulted in tumor growth accompanied by an increase in PMN‐MDSCs and a decrease in CD8^+^ T cells, while these effects were mitigated by silencing *HNRNPAB* or *TGFB2*, or by applying anti‐TGFβ antibody (Figure 5F–I; Figure S13H, Supporting Information). Summarily, *tRF‐22* likely prompts tumor growth through a “hnRNPAB–TGFβ2–PMN‐MDSCs–CD8^+^ T cell” axis.

To assess the broader relevance of *tRF‐22*, we analyzed its expression and its correlation with survival across 17 additional common cancer types in the TCGA database. While certain clinical associations were identified, substantial heterogeneity was observed. For instance, *tRF‐22* was significantly upregulated in stomach adenocarcinoma (STAD) and bladder cancer (BLCA) compared to adjacent normal tissues but showed no correlation with patient survival, suggesting its involvement in tumorigenesis rather than progression. In contrast, while *tRF‐22* expression didn't differ significantly between bile duct cancer (CHOL) and adjacent normal tissues, higher expression was associated with improved survival, indicating its role in tumor progression rather than tumorigenesis in this cancer type (Figure S14A,B, Supporting Information). Collectively, these results suggest that *tRF‐22* may exert diverse biological functions depending on the tumor context.

### 2.6 *tRF‐22* Inhibition Combining TGFβ2 Blockade Enhances ICB Efficacy in Eradicating ESCC

**Figure 6:**
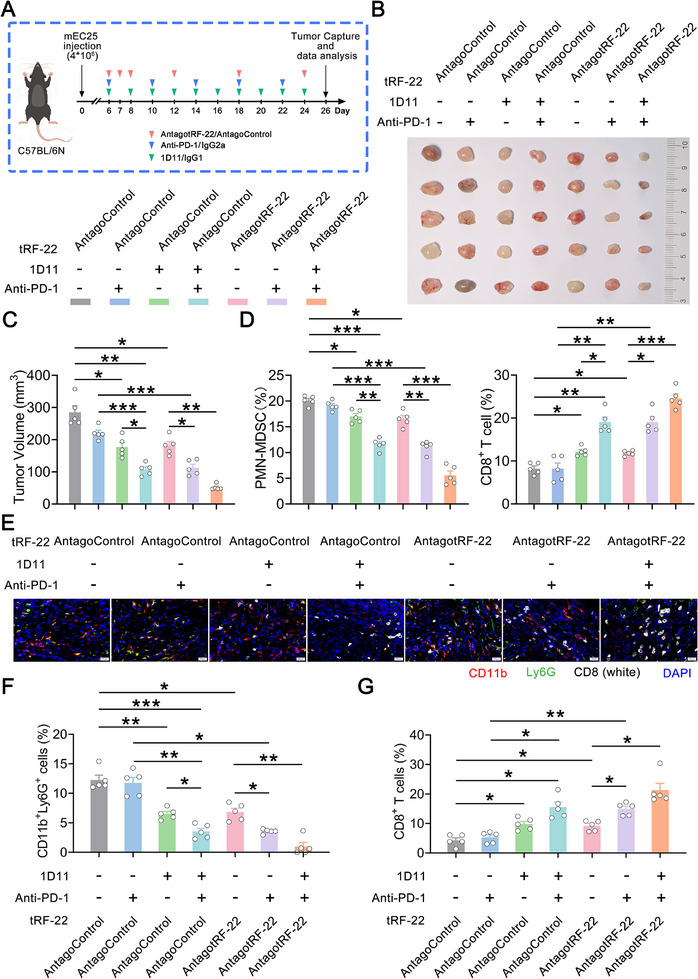
*tRF‐22* inhibition and TGFβ2 blockade enhance ICB efficacy in ESCC. A) Schematic diagram of treatment regimens involving AntagotRF‐22, Anti‐PD‐1, and Anti‐TGFβ, used individually or in combination in ESCC mouse models. Image B) and quantification C) of tumors obtained after receiving treatments indicated above (*n* = 5 per group). D) The proportions of CD11b^+^Gr‐1^+^Ly6G^+^ PMN‐MDSCs (*middle panel*) and CD8^+^ T cells (*right panel*) in these tumors were determined by flow cytometry. E–G) The abundance of CD11b^+^Ly6G^+^ PMN‐MDSCs and CD8^+^ T cells within these tumors (*n* = 5 per group). Representative mIHC staining images (E) and quantification (F, G). E) Scale bars, 20 µm. (C, D, F, G) Data presented as mean ± SEM. ^*^, *p* < 0.05; ^**^, *p* < 0.01; ^***^, *p* < 0.001 by Brown‐Forsythe ANOVA with Dunnett's T3 multiple comparison test. All experiments were performed at least three times.

We investigated whether *tRF‐22* inhibition and/or TGFβ2 blockade could improve ICB efficacy for ESCC. We administered antagotRF‐22, anti‐TGFβ antibody, and anti‐PD‐1 antibody, alone or in combination, in a subcutaneous mEC25 tumor model (**Figure** **6**A). Single anti‐PD‐1 antibody therapy resulted in only a slight reduction in tumor size, indicating that ICB alone was insufficient. In contrast, anti‐TGFβ antibody or antagotRF‐22 led to a moderate reduction of PMN‐MDSCs, an increase in CD8^+^ T cells, and smaller tumor lesions. Combining either anti‐TGFβ antibody or antagotRF‐22 with anti‐PD‐1 antibody resulted in a significant decrease in PMN‐MDSCs, an increase in CD8^+^ T cells, and a dramatic impairment in tumor growth. Notably, triple blockade of *tRF‐22*, TGFβ2, and PD‐1 nearly eradicated tumor lesions (Figure 6B–D; Figure S15A, Supporting Information). In addition, we applied the same treatments to ESCC tumors derived from *tRF‐22*‐deficient mEC25 cells. We found that antagotRF‐22 failed to enhance the efficacy of anti‐PD‐1, either alone or in combination with anti‐TGFβ, in such models with low *tRF‐22* levels (Figure S15B–F, Supporting Information). Excellently, combined antagotRF‐22, anti‐PD‐1 antibody, and anti‐TGFβ antibody showed low toxicity, as indicated by stable body weight (Figure S15G, Supporting Information). mIHC assays further verified the results of flow cytometry (Figure 6E–G). In summary, these results declare that the combined targeting of *tRF‐22* and TGFβ2 reduces PMN‐MDSCs infiltration, alleviates CD8^+^ T cells suppression, and improves the ICB efficacy in ESCC.

### 2.7 *tRF‐22* Exhibits Strong Potential for Evaluating Immunotherapy Efficacy in ESCC

**Figure 7:**
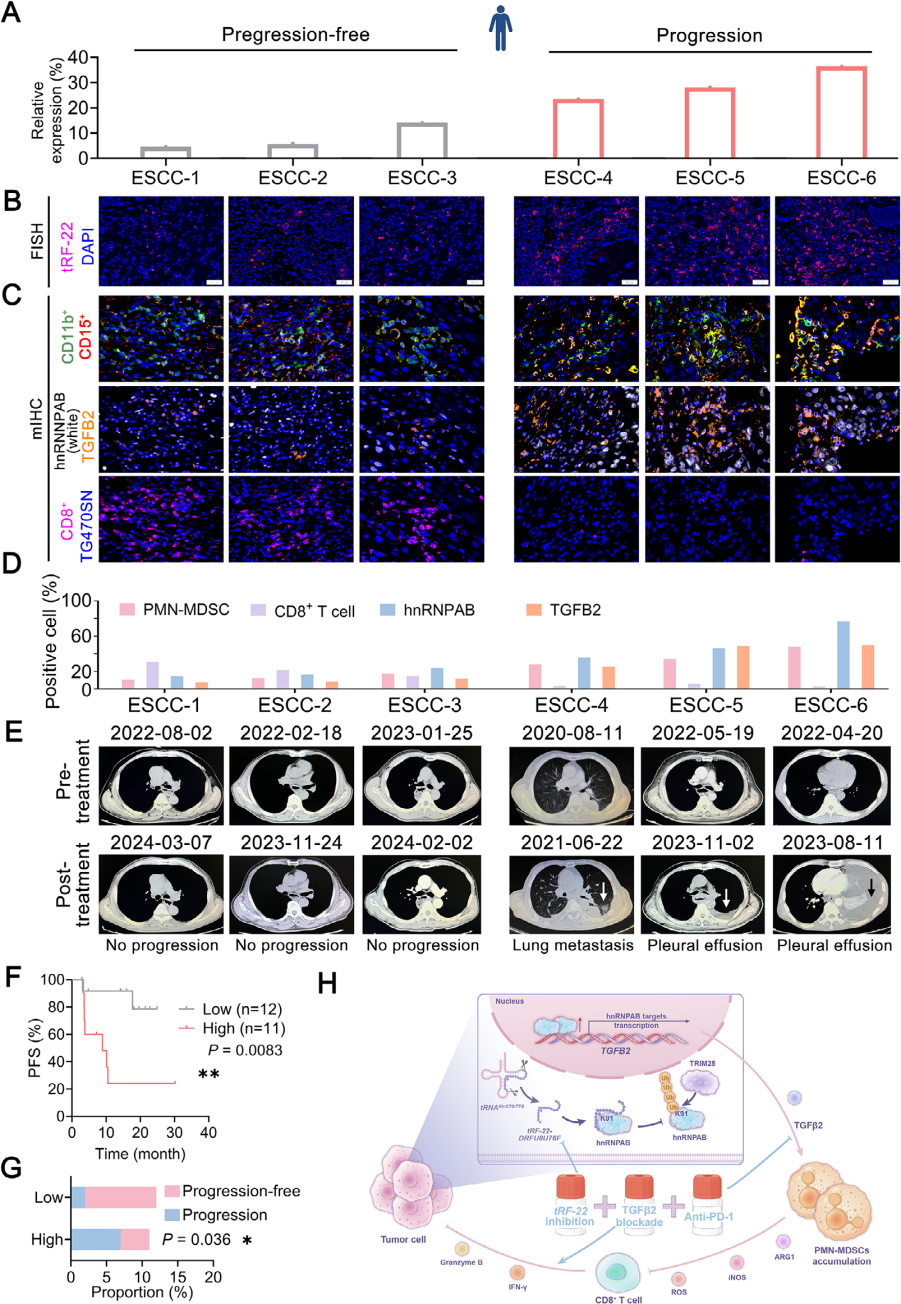
*tRF‐22* expression correlated with the ICB efficacy and prognosis of postoperative ESCC patients. A, B) The percentage (A) and representative images (B) of *tRF‐22* positive cells in representative ESCC patients by RNA FISH (*n* = 3 per group). Scale bars, 50 µm. C, D) The abundance of CD11b^+^CD15^+^ PMN‐MDSCs, CD8^+^ T cells, hnRNPAB, and secreted TGFβ2 levels within tumor tissues from these representative patients. Representative mIHC staining images (C) and quantification (D). E) The biomedical images of the postoperative ESCC patients before treatment (*upper panel*) and after treatment (*lower panel*). F) Kaplan–Meier estimates of patients’ progression‐free survival time in our ESCC‐ICB cohort (*n* = 23) according to *tRF‐22* levels. *P* values were determined by the log‐rank test. ^**^, *p* < 0.01. G) The ESCC‐ICB cohort with ICB treatment in our hospital was included. *tRF‐22* levels were also significantly correlated with the PFS of patients. ^*^, *p* < 0.05 by Fisher's exact test. H) Schematic diagram depicting *tRF‐22* drives immune suppression and the novel strategy to overcome ICB resistance in ESCC.

Having clarified the powerful abilities of *tRF‐22* in improving the efficacy of ICB therapy in mouse models, we then assessed its potential for evaluating the effectiveness of postoperative immunotherapy in ESCC patients. *tRF‐22* levels, measured by RNA FISH, were higher in patients with disease progression, such as lung metastasis or pleural effusion, compared to those who were progression‐free (**Figure** **7**A,B,E). We also examined the number of PMN‐MDSCs and CD8^+^ T cells, as well as the protein levels of hnRNPAB and TGFβ2 in ESCC tissue specimens by mIHC assays. As *tRF‐22* levels rose, hnRNPAB, TGFβ2 protein levels, and PMN‐MDSC number also increased, while CD8^+^ T cell number decreased (Figure 7C,D). Kaplan‐Meier estimates showed that patients with higher *tRF‐22* levels had shorter progression‐free survival compared with those with lower *tRF‐22* levels, making it a potential biomarker for predicting immunotherapy outcomes (Figure 7F,G). These results demonstrate that *tRF‐22* fosters an immunosuppressive microenvironment through hnRNPAB and TGFβ2‐mediated PMN‐MDSCs accumulation. Dual targeting *tRF‐22* and TGFβ2 could improve ICB therapy effectiveness in ESCC patients (Figure 7H).

## 3 Discussion

Immunosuppressive TME is a key contributing factor for the low response rate to ICB therapy in patients with ESCC.^[^ ^11^ ^]^ However, the intricate interactions between tumor cells and TME have yet to be fully elucidated. In the present study, we have demonstrated that tumorigenic *tRF‐22* enhances the stabilization of hnRNPAB, increases the expression and secretion of TGFβ2, and reinforces the infiltration of PMN‐MDSCs into tumors, which collectively contribute to immune suppression and ICB resistance in ESCC. We also found that targeting *tRF‐22* with antagotRF‐22 reagents potentiated the efficacy of PD‐1 blockade and constrained tumor growth. All of these experimental findings, along with the observed relationship between *tRF‐22* levels and ICB efficacy in ESCC patients, may provide a novel strategy for patient selection and combination therapy to overcome ICB resistance in *tRF‐22*‐overexpressing ESCC.

Numerous studies have highlighted the critical roles that tRFs play in tumor cell processes.^[^ ^24^, ^25^ ^]^ Typically, four specific tRFs induced by serum starvation suppress breast cancer metastasis by destabilizing key oncogenic transcripts.^[^ ^30^ ^]^ *LeuCAG3′tsRNA* represses liver cancer cell apoptosis by binding to RPS28 mRNA and enhancing its translation.^[^ ^28^ ^]^ Our previous work also demonstrated that inflammatory cytokine‐regulated *tRF‐21* inhibits the proliferation and metastasis of pancreatic cancer cells by modulating the alternative splicing of key genes.^[^ ^31^ ^]^ Despite these findings, there has been limited exploration of tRFs' roles in regulating the TME. Some sequencing works have revealed that tRFs are abundant in the cytoplasm of immune cells, with predominantly 5′ tRFs being selectively secreted into the extracellular compartment as immune signaling molecules to support the immune response.^[^ ^32^, ^42^, ^43^ ^]^ However, these studies have not explored the underlying mechanisms or evaluated the potential clinical applications of tRFs. In this study, we identified a certain tRF (i.e., *tRF‐22*) that is significantly associated with the prognosis of ESCC patients and found that *tRF‐22* significantly promotes tumor growth in immunocompetent C57BL/6N mice, but not in immunodeficient nude mice. This striking contrast suggests that *tRF‐22* primarily exerts its influence by shaping the TME in ESCC. Further experiments validated that *tRF‐22* promotes tumor growth by enhancing PMN‐MDSCs infiltration, thus expanding our knowledge of the full scope of tRFs' biological activities.

The present study uncovers that intracellular *tRF‐22* binds to Lys91 in the RRM1 domain of the oncogenic RNA‐binding protein hnRNPAB, preventing its ubiquitination by TRIM25, which in turn stabilizes the hnRNPAB protein and increases its protein levels. Notably, we are the first to report hnRNPAB as a new substrate of the known E3 ligase TRIM25. Additionally, we propose for the first time that *tRF‐22* could disrupt the interaction between hnRNPAB and TRIM25, potentially reducing hnRNPAB ubiquitination and altering its subsequent function. We further determined *TGFB2* as a direct target gene of hnRNPAB through our unbiased high‐throughput RNA sequencing and subsequent CUT&RUN experiments. TGFβ2 is an important enhancer involved in MDSCs generation induced by IL‐6 and GM‐CSF.^[^ ^20^, ^23^ ^]^ In line with the results of our scRNA‐seq and flow cytometry, *tRF‐22* significantly promoted MDSCs, particularly PMN‐MDSCs expansion. All these discoveries advance our understanding of the roles of endogenous tRFs in the interaction between tumor cells and MDSCs within the TME.

It has been reported that the overall response rate (ORR) of single‐agent and dual‐agent ICB with anti‐PD‐1/PD‐L1 and/or anti‐CTLA4 in patients with ESCC in the overall population was 19.3% and 28%, respectively.^[^ ^44^, ^45^ ^]^ These findings indicate that the combination of a single immunosuppressive cellular or molecular component with ICB shows limited effectiveness in improving the overall efficacy of ICB. Therefore, greater efforts should be directed toward combining integrated targets and stratifying patients based on biomarkers to improve efficacy. Given the crucial roles of *tRF‐22* in ESCC TME, we treated tumor‐bearing C57BL/6N mice with antagotRF‐22 reagents. As expected, treatment with single antagotRF‐22 partially inhibits tumor growth. Notably, both single and combined treatments with antagotRF‐22 and TGFβ2 neutralizing antibody enhanced the efficacy of anti‐PD‐1 antibody, collectively highlighting the potential value of *tRF‐22* in combined therapies. Additionally, we examined the *tRF‐22*–hnRNPAB–TGFβ2–PMN‐MDSCs–CD8^+^ T cells regulatory axis in ESCC patient specimens from those receiving immunotherapy. We observed that specimens with high *tRF‐22* levels also exhibited elevated levels of hnRNPAB and TGFβ2, along with increased proportions of PMN‐MDSCs and reduced proportions of CD8^+^ T cells. *tRF‐22* was detectable in the serum of both healthy individuals and ESCC patients, with significantly higher expression levels observed in ESCC patients. Moreover, its expression was higher in advanced tumor stages than in early stages, suggesting its potential as a circulating biomarker. Although *tRF‐22* has been demonstrated to have the potential as a reliable predictor for ICB efficacy in a cohort of 23 ESCC patients and a circulating biomarker, it is crucial to validate these findings in a larger cohort.

While gender differences in ESCC incidence have been reported and gender is recognized as an important factor influencing immune responses in multiple cancer types,^[^ ^46^ ^]^ clinical studies examining the association between gender and immunotherapy response have yielded inconsistent results.^[^ ^47^ ^]^ In our study, extensive animal experiments were conducted to investigate this relationship, and the results clearly demonstrated that the immune‐regulatory role of *tRF‐22* is independent of sex. Furthermore, pan‐cancer analysis across 17 additional common cancer types in the TCGA database revealed considerable heterogeneity in *tRF‐22* biology. Potential mechanisms underlying this variability may include *tRF‐22* targeting genes or signaling pathways beyond hnRNPAB in other cancer types, as well as its regulation being influenced by co‐expressed regulatory factors or transcriptional variations. Even if *tRF‐22* interacts with hnRNPAB, its regulation of hnRNPAB's epigenetic modifications, protein abundance, and biological functions may vary across cancer types.

Altogether, our study revealed the critical role of tumor cell‐derived tRFs in shaping immunosuppressive TME and limiting the efficacy of anti‐PD‐1 treatment, providing a novel mechanism‐based candidate strategy for combination therapy to overcome ICB resistance in ESCC.

## 4 Conclusion

In summary, we identified a *tRF‐22*–hnRNPAB–TGFβ2–PMN‐MDSCs–CD8^+^ T cell pathway that illustrates the formation of the immunosuppressive TME in ESCC, providing a novel mechanism‐based candidate strategy for combination therapy to overcome ICB resistance in ESCC.

## 5 Experimental Section

### Clinical Specimens

Surgically removed ESCC samples and paired adjacent normal tissues (*n* = 100) were collected from Qilu Hospital of Shandong University between 2017 and 2023 (Table S4, Supporting Information). ESCC was diagnosed by histopathology, assessed by at least three pathologists, and clinical information was obtained from medical records. All patients underwent esophagectomy, and none of them received any preoperative anti‐cancer treatment. Overall survival (OS) time was defined as the duration from diagnosis to the date of last follow‐up or death, while progression‐free survival (PFS) time was the time from diagnosis to relapse or disease progression. Survival status was determined through medical records, patient families, or follow‐up calls. ESCC and the corresponding adjacent normal tissue samples were collected at the time of esophagectomy and were immediately frozen and stored in liquid nitrogen until use. For the ESCC‐ICB cohort, 23 patients receiving postoperative ICB treatment were included in Qilu Hospital (Table S5, Supporting Information). Blood samples were collected from 30 ESCC patients and 30 healthy individuals in BD red cap Vacutainer serum tubes. All samples were allowed to clot at room temperature for 30 min, followed by centrifugation at 1800 g for 10 min. The serum was transferred to new tubes and centrifuged at 13 000g for 10 min to remove residual cells and fragments. Total RNA was then extracted from 200 µL of serum using the miRNeasy Serum/Plasma Kit (QIAGEN) according to the manufacturer's instructions for subsequent RT‐qPCR analysis. All samples were obtained with informed consent, and the study was approved by the medical science research ethics committee of Qilu Hospital of Shandong University (Ethics Code: KYLL‐2022‐ZM‐721).

### Cell Lines

Two human ESCC cell lines (KYSE150 and KYSE30) and 293T cells were purchased from the Cell Bank of Type Culture Collection of the Chinese Academy of Sciences, Shanghai Institute of Biochemistry and Cell Biology. Mouse ESCC cell line mEC25 was donated by Prof. Fu from the International Cancer Center of Shenzhen University.^[^ ^48^ ^]^ Briefly, the primary ESCC tissue from the esophagus of 4‐NQO‐treated C57BL/6N mouse was transplanted into immun incomplete BALB/c nude mice to further enhance the tumorigenicity of cancer cells. The explants were successfully established and resected to isolate and purify primary ESCC cells by removing CAFs, which disturbs the generation of cancer cell clones. Mouse ESCC cell lines mEC586F and mEC525M were kindly provided by Prof. Liu from the Cancer Hospital of Chinese Academy of Medical Sciences. The stable mEC25, mEC586F, and mEC525M cell lines exhibited potent tumorigenicity in both C57BL/6N and BALB/c nude mice. KYSE150 and KYSE30 cells were cultured in RPMI 1640 with 10% FBS, while 293T, mEC25, mEC586F, and mEC525M cells were cultured in DMEM with 10% FBS, and they were incubated at 37 °C in a humidified atmosphere with 5% CO_2_. All cell lines tested negative for Mycoplasma bacteria.

### Animal Experiments

C57BL/6N mice and BALB/c nude mice used in this study were purchased from Beijing Vital River Laboratory Animal Technology, and acclimated for 1 week under a 12 h dark/12 h light cycle with adequate food and water. Animal experiments were carried out in compliance with and approved by the Shandong University Specific Pathogen Free (SPF)‐Animal Center. All experimental animal procedures were approved by the Animal Care and Animal Experiments Committee of Shandong University (Ethics Code: DWLL‐2023‐158).

### Establishment of Mouse Models

Human ESCC cell lines (KYSE150 and KYSE30) and mouse ESCC cell line (mEC25) was infected with lentiviruses carrying control (scrambled sequence), *tRF‐22* sense, or *tRF‐22* antisense sequence. Mouse ESCC cell lines (mEC586F and mEC525M) were infected with lentiviruses carrying control or *tRF‐22* antisense sequence. 4 × 10^6^ mouse cells were harvested in 100 µL PBS containing 50 µL Matrigel and subcutaneously inoculated in the groin area of 5‐week‐old female or male C57BL/6N mice.^[^ ^48^, ^49^, ^50^, ^51^ ^]^ 3 × 10^6^ human ESCC cell lines or mouse ESCC cell lines in 100 µL PBS (with 50 µL Matrigel for mouse cells) were subcutaneously injected into the hind legs of 5‐week‐old female or male nude mice. Tumor volumes were measured every three days with a vernier caliper and are expressed as 0.5 × length × width.^2^

### Treatment of ESCC Allografts

For *tRF‐22* inhibition, antagotRF‐22, a single strand *tRF‐22* with 2 phosphorothioates at the 5′ end, 4 phosphorothioates and one cholesterol group at the 3′ end, and one full‐length nucleotide 2′‐methoxy modification, together with the antagoControl, were synthesized and administrated in saline at the dose of 40 mg kg^−1^, i.v., every day for 3 days and then every 6 days for additional 3 times. For PD‐1 blockade, 200 µg anti‐PD‐1 antibody (i.p. every fourth day, clone 29F.1A12, BioxCell) or 200 µg anti‐IgG2a antibody (i.p. every fourth day, clone 2A3, BioxCell) was injected into mice. For TGFβ2 inhibition, 5 mg kg^−1^ anti‐TGFβ antibody (i.p. every other day, clone 1D11, BioxCell) or 5 mg kg^−1^ anti‐IgG1 antibody (i.p. every other day, Clone MOPC‐21, BioxCell) was administered to mice. And for PMN‐MDSCs elimination, 200 µg anti‐Ly6G antibody (i.p. every fourth day, clone 1A8, BioxCell) or 200 µg anti‐IgG2a antibody (i.p. every fourth day, clone 2A3, BioxCell) was administered to mice. For PMN‐MDSCs adoptive transfer, 4 × 10^6^ cells (i.v. every fourth day) were injected into mice.

### Analysis of Public Data

The raw small RNA sequence files (.bam files) were downloaded from the Cancer Genomics Hub (CGHub)^[^ ^52^ ^]^ with permission of the data access committee and extracted raw reads (FASTQ format) from BAM files of the TCGA database using the “bamToFastq”, a subcommand of the genome arithmetic toolset “BEDtools2” (v2.25.0). FASTQ files were analyzed with MINTmap^[^ ^53^ ^]^ software for the identification of tRFs and their abundance. tRFs with RPM ≥ 0.1 was chosen and presented in > 80% of total samples, and by this definition, 714 tRFs in the TCGA ESCC dataset were selected for further analysis. The correlations between the tRFs levels and survival time in patients with ESCC in the TCGA database were estimated by Kaplan–Meier plot with log‐rank test and univariate Cox regression analysis. *p* < 0.05 was considered significant. The correlation between *tRF‐22* levels and survival across various cancer types in the TCGA database was also analyzed using the same methods employed for ESCC. In the pan‐cancer analysis of *tRF‐22* expression, only cancer types with at least three tumor and adjacent normal tissue samples were included, with differences assessed using the Wilcoxon test.

### Molecular Docking

The X‐ray crystal structures of hnRNPAB (ROAA, 3S7R) and TRIM25 (6FLN) were retrieved from the Protein Data Bank. To ensure the accuracy of the docking results, the proteins were prepared using the AutoDockTools, and the water molecules were manually eliminated from the protein, and the polar hydrogen was added. Docking Web Server (HDOCK) was used for protein‐RNA/protein docking. The protein‐RNA/protein complex and its interactions were prepared by AutoDockTools‐1.5.7 and visualized by PyMOL.

### Analysis of Cell Malignant Phenotypes

Cell viability was measured with the CCK‐8 kit (Dojindo) by microplate assay. Colony formation ability was determined by the number of methanol‐fixed and crystal violet‐stained cell colonies. Cell invasion ability was measured by counting the methanol‐fixed and crystal violet‐stained cells crossed from serum‐free medium in the upper chamber through the Millicell chamber with 8‐µm pores coated with 30 µg of Matrigel (Corning). Migration ability was tested using the same method, but the inserts were not coated with Matrigel.

### Single‐Cell RNA Sequencing and Analysis—*Tissue Dissociation and Single‐Cell Suspensions Preparation*

Fifteen days after injecting C57BL/6N mice with 4 × 10^6^ mEC25 cells with *tRF‐22* silenced or control, tumor tissues were cut into small pieces and dissociated in a solution containing collagenase IV, papain, and DNase I at 37 °C for 20 min. Erythrocytes and dead cells were removed using 1 × erythrocyte lysis solution and Dead Cell Removal MicroBeads (Miltenyi Biotec), with cell activity assessed at > 85% using trypan blue.

### Chromium 10x Genomics Library and Sequencing

Single‐cell suspensions were processed with the 10x Genomics Chromium Single‐Cell 3′ kit (V3). Libraries were sequenced on an Illumina NovaSeq6000 system by LC‐Bio Technology.

### Bioinformatics Analysis

Sequencing data were converted to FASTQ format and aligned to the reference genome using CellRanger. The data were filtered for quality, clustered, and analyzed using Seurat (version 4.1.0).

### Flow Cytometry Analysis

Freshly isolated tumor tissues were cut into small pieces and dissociated into single‐cell suspensions by using Singleron PythoN Automated Tissue Dissociation System, followed by filtering with 70‐µm cell strainers (Biosharp). 1 × Viability Dye 780 (biogems) was stained in the dark at 4 °C for 30 min, and subsequently CD16/32 antibody (Fc block) at room temperature for 15 min. Cell surface markers were stained with fluorophore‐conjugated antibodies in the dark at room temperature for 20 min. For intracellular marker staining, Fixation/Permeabilization buffer was used to incubate cells at 4 °C for 45 min before staining with fluorophore‐conjugated antibodies at 4 °C for 1 h. Flow cytometric analysis was performed on a BD FACS Celesta Multicolor Flow Cytometer, and data were analyzed using FlowJo software. All the fluorophore‐conjugated antibodies used in this study are shown in Table S6 (Supporting Information).

### RT‐qPCR

Total RNA was extracted from cells or tissue samples with the Total RNA Extraction Kit (Fastagen). The rtStarTM tRF and tiRNA Pretreatment Kit (Arraystar) was used to pretreat modifications of RNA samples, and the Evo M‐MLV RT Kit with gDNA Clean for qPCR II (Accurate Biology) was used to reverse transcribe pretreated RNA into cDNA with random primers or specific tRF stem‐loop RT primers (custom‐made by GenePharma). Relative RNA level was determined by quantitative real‐time PCR on a CFX Connect Real‐Time System (BIO‐RAD) with SYBR Green reagents (Accurate Biology). *U6* and *ACTB* RNA were used as an internal control for quantification of tRFs and mRNAs, respectively. Each experiment had three biological replicates, and the relative expression level was determined with the 2^−ΔCt^ method. The primers were listed in Table S7 (Supporting Information).

### Western Blot

Cells and tissue samples were lysed with 1× RIPA buffer supplemented with the Protease/Phosphatase Inhibitor Cocktail (Pierce), and the lysate was high‐speed centrifuged to collect the supernatant. About 20 µg total protein was subjected to SDS‐PAGE and transferred to a PVDF membrane (Millipore). Specific primary antibody was incubated overnight at 4 °C, and secondary antibody at room temperature for 1 h. Immunoblotting bands were visualized with a SuperSignal West Pico PLUS Chemiluminescent Substrate (Thermo Scientific). The western blot images in this study represent one of three independent replicates. All antibodies in this study are listed in Table S6 (Supporting Information).

### Northern Blot

Total RNA (15 µg) from ESCC cells was pre‐heated at 90 °C and then subjected to 15% denaturing urea‐polyacrylamide gel electrophoresis and then transferred to Biodyne Nylon Membrane (Pall). After pre‐hybridization for 30 min, the membrane was hybridized overnight at 42 °C in DIG Easy Hybrid buffer containing the denatured *tRF‐22* probe labeled with digoxigenin (Table S7, Supporting Information). After washing, the membrane was incubated with anti‐digoxigenin‐AP (Table S6, Supporting Information), and the signal on the membrane was detected by using an Odyssey infrared scanner (Li‐Cor, Lincoln).

### Plasmids Construction, Transient and Stable Transfection

Lentivirus‐mediated tRF overexpression or silencing had been described before.^[^ ^30^ ^]^ In short, the synthesized *tRF‐22* antisense sequence was inserted into the pLent‐U6‐shRNA‐CMV‐luciferase‐P2A‐puro vector. For overexpression, the *tRF‐22* sequence was inserted into the same vector containing the miR‐30 backbone (WZ Biosciences). Similarly, human *HNRNPAB*, mouse *hnrnpab*, or *tgfb2* shRNA sequences were inserted into the pLent‐U6‐shRNA‐CMV‐copGFP‐P2A‐Puro vector. To construct the shRNA‐resistant mutant plasmid, a synonymous mutation was introduced at amino acids 186–192 in the coding sequence of mouse *hnrnpab* (NM_001048061.1) and the mutated sequence was subsequently inserted into the pLent‐U6‐shRNA‐CMV‐copGFP‐P2A‐Puro vector. Human *HNRNPAB* cDNA sequence was subcloned into the pLent‐EF1a‐FH‐CMV‐copGFP‐P2A‐Puro vector. For constructing FLAG‐tagged *HNRNPAB* expression plasmids, synthesized full‐length, truncated, or mutated *HNRNPAB* cDNA was respectively subcloned into the pcDNA3.1‐C‐3×FLAG‐P2A‐GFP vector. For constructing MYC‐tagged *TRIM25* expression plasmids, synthesized full‐length or truncated *TRIM25* cDNA was subcloned into the pcDNA3.1‐C‐myc‐P2A‐GFP vector. The same method was used for constructing MYC‐tagged *MKRN2* expression plasmids. All plasmids and their insertion sequences were authentic by DNA sequencing.

For stable transfection, plasmids and lentiviral packaging system (OBiO Technology) were then co‐transfected into 293T cells using Lipofectamine 3000. The resultant lentiviruses were collected and infected human or mouse ESCC cells in the presence of polybrene (Sigma–Aldrich). For transient transfection, plasmids, siRNAs, or *tRF‐22* inhibitor were transfected into human or mouse ESCC cells using Lipofectamine 3000. The siRNAs and inhibitors used in this study are shown in Table S8 (Supporting Information).

### RNA FISH

RNA FISH was performed with the Fluorescence in Situ Hybridization kit (SA‐Biotin System, GenePharma). Adherent cells, after being fixed and permeabilized, were hybridized with Biotin‐labeled *tRF‐22* probes (GenePharma) overnight in a humidified chamber at 37 °C in the dark. Paraffin sections, after dewaxed, were digested by proteinase K and denatured, were hybridized with *tRF‐22* probes overnight in a humidified chamber at 37 °C in the dark. Cell nuclei were counterstained with DAPI. The SA‐Biotin System could amplify the signals and be detected using 4′,6‐diamidino‐2‐phenylindole and Cy3 channels captured by SpinSR10 spinning disk confocal super resolution microscope (Olympus) or SLIDEVIEW VS200 research slide scanner (Olympus).

### RNA Pulldown Assay

RNA pulldown assays were performed using the Pierce Magnetic RNA‐Protein Pull‐Down Kit (Thermo Scientific). In short, the Biotin‐labeled *tRF‐22* probes (GenePharma) were bound to streptavidin magnetic beads, and then the cell lysate was added to construct the RNA‐Protein binding reaction system on a rotator overnight at 4 °C. The RNA‐binding protein complexes were subjected to Western blot or further mass spectrum analysis.

### Protein Immunoprecipitation Assay and Mass Spectrometry Analysis

Protein immunoprecipitation was performed using the Pierce Crosslink Magnetic IP/Co‐IP Kit (Thermo Scientific). Briefly, a specific antibody to the interested protein was bound to protein A/G magnetic beads and crosslinked by DSS (disuccinimidyl suberate). Then the prepared cell lysate was added and incubated overnight at 4 °C on a rotator. The next day, the magnetic bead‐bound proteins were eluted for western blotting or mass spectrometry analysis.

Eluate containing proteins associated with *tRF‐22* from RNA pulldown assays or proteins co‐precipitated with hnRNPAB from co‐immunoprecipitation assays was digested into peptides and then subjected to the mass spectrometer Q‐Exactive (Thermo Scientific). Proteome Discoverer software was then used for analysis, and proteins were ranked by the Score Sequest HT or Abundance.

### RNA Immunoprecipitation Assay

RNA immunoprecipitation (RIP) assays were performed with the Magna RIP RNA‐Binding Protein Immunoprecipitation kit (Millipore). In a nutshell, the antibody against RBP of interest was first immunoprecipitated with protein A/G magnetic beads, and then the cell lysate prepared in advance was put in. Magnetic beads bound complexes were immobilized, and RNAs were extracted for RT‐qPCR analysis.

### Cell Proliferation Assay

CD8^+^ T cells were isolated from the splenocytes of healthy tumor‐free mice by using CD8a (Ly‐2) microbeads (130‐117‐044, Miltenyi Biotec). PMN‐MDSCs were isolated from tumor tissues with the Myeloid‐Derived Suppressor Cell Isolation Kit (Miltenyi Biotec, 130‐094‐538). CD8^+^ T cells labeled with CFSE (5,6‐carboxyfluorescein diacetate succinimidyl ester, 5 µm, Thermo Scientific) were co‐cultured with different ratios of PMN‐MDSCs in the presence of mouse T‐Activator CD3/CD28 dynabeads (11456D, Thermo Scientific) and recombinant human IL‐2 (200‐02, PeproTech) for 3 days. Flow cytometry analysis was used for detecting the CFSE dilution rate of CD8^+^ T cells, and FlowJo software was used for analysis.

### MDSCs Generation—*Generation of Human MDSCs from PBMCs*

Human PBMCs were isolated from healthy donors, and subsequently, differential density gradient separation by Ficoll. PBMCs were cultured in 6‐well plates with ultra‐low attachment surface at 1 × 10^6^ cells per mL in conditioned medium from KYSE150 Control, *tRF‐22* KD, or *tRF‐22* OE with or without *HNRNPAB* KD, *TGFB2* KD, or anti‐TGFβ antibody (clone 1D11, BioxCell), supplemented with recombinant human GM‐CSF (40 ng mL^−1^, 300–03, PeproTech) and recombinant human IL‐6 (40 ng mL^−1^, 200–06, PeproTech) for 7 days. Medium and cytokines were refreshed every 2‐ 3 days during this week. Flow cytometry analysis was used to characterize the CD11b^+^CD33^+^HLA‐DR^−^ populations as early‐stage MDSCs.

### Adoptive Transfer of MDSCs

Total BM cells were isolated from tumor‐bearing C57BL/6N mice as described before.^[^ ^54^ ^]^ In brief, BM cells were cultured in complete medium supplemented with 40 ng mL^−1^ GM‐CSF and IL‐6 at 37 °C in a 5% CO_2_‐humidified atmosphere for 5 days. BM‐derived MDSCs enriched by Myeloid‐Derived Suppressor Cell Isolation Kit were intravenously injected into the mice with 4 × 10^6^ cells per mouse for adoptive transfer.

### Multiplex Immunohistochemistry (mIHC)—*Mouse Tumor Samples*

mIHC staining of formalin‐fixed, paraffin‐embedded (FFPE) mouse tumor samples was performed using the Treble‐Fluorescence immunohistochemical mouse/rabbit kit (Immunoway, RS0035). Primary antibodies were listed in Table S6 (Supporting Information). Each staining employed a secondary HRP‐conjugated antibody with a tyramide‐coupled fluorophore: Opal 520 (CD8), Opal 620 (CD11b), Opal 650 (Ly6G), and 4′,6‐diamidino‐2‐phenylindole (DAPI). Images were captured using SLIDEVIEW VS200 research slide scanner (Olympus).

### ESCC Patients’ Tumor Samples

mIHC staining of FFPE ESCC patients’ tumor samples was conducted with TSA Multiplex IHC Assay Kits (TissueGnostics). Each staining used a secondary HRP‐conjugated antibody with a tyramide‐coupled fluorophore: TG520N (CD8), TG660S (CD11b), TG570N (CD15), TG440N (hnRNPAB), TG650N (TGFβ2), and TG470SN (nuclei). Images were captured with the TissueFAXS spectral system (TissueGnostics).

### Bulk RNA‐Seq

KYSE150 cells individually transfected with *tRF‐22* inhibitor, *HNRNPAB* siRNA, or co‐transfected with control sequences (3 replications per group) were collected for RNA extraction and sequencing library construction. After size selection and PCR amplification, sequencing was performed using 2 × 150 bp paired‐end reads on an Illumina Novaseq 6000 (LC‐Bio Technology).

After sequencing, clean reads were filtered, aligned to the reference genome, and gene abundance was quantified. Differentially expressed genes (DEGs) were identified using DESeq2, focusing on genes with an FDR below 0.05 and an absolute fold change ≥ 2 for KEGG pathway enrichment analysis.

### tRNA‐Seq and tsRNA‐Seq—*tRNA‐Seq*

mEC25 cells were treated with either antagoControl or antagotRF‐22 (3 replications per group) and then subjected to tRNA‐seq (Aksomics, Shanghai). tRNAs were purified from total RNA samples, demethylated at m^1^A and m^3^C positions, and partially hydrolyzed according to the Hydro‐tRNAseq method. Then, partially hydrolyzed and re‐phosphorylated tRNA fragments were converted into small RNA sequencing libraries using the NEBNext Multiplex Small RNA Library Prep Set for Illumina kit (New England Biolabs). Size selection was performed on PCR‐amplified fragments (≈140–170 bp, corresponding to ≈19–50 nt tRNA fragments). The resulting tRNA‐seq libraries were assessed for quality and quantified using the Agilent 2100 BioAnalyzer. Libraries were pooled and sequenced on an Illumina sequencer according to the manufacturer's instructions. Sequencing quality was evaluated with FastQC, and trimmed reads were aligned to cytoplasmic mature tRNA sequences from GtRNAdb and mitochondrial tRNA sequences from mitotRNAdb using BWA software. Differentially expressed tRNAs were identified based on count values using the R package edgeR.

### tsRNA‐Seq

mEC25 cells were treated with either antagoControl or antagotRF‐22 (3 replications per group) and then subjected to tsRNA‐seq (Aksomics, Shanghai). Total RNA samples were first pretreated to remove modifications that interfere with small RNA‐seq library construction, including 3′‐aminoacyl (charged) deacylation to 3′‐OH for 3′‐adaptor ligation, 3′‐cP (2′,3′‐cyclic phosphate) removal to 3′‐OH for 3′ ‐adaptor ligation, 5′‐OH (hydroxyl group) phosphorylation to 5′‐P for 5′ ‐adaptor ligation, and m^1^A and m^3^C demethylation for efficient reverse transcription. The pretreated total RNA was then used to prepare the sequencing library through the following steps: 1) 3′ ‐adapter ligation; 2) 5′ ‐adapter ligation; 3) cDNA synthesis; 4) PCR amplification; 5) size selection of 134–160 bp PCR amplified fragments (corresponding to 14–40 nt small RNA size range). The libraries were denatured as single‐stranded DNA molecules, captured on Illumina flow cells, amplified in situ as sequencing clusters, and sequenced on an Illumina sequencer according to the manufacturer's instructions. Sequencing quality was assessed using FastQC. The 5′ and 3′‐adaptor sequences were trimmed using Cutadapt. Trimmed reads were aligned with one mismatch allowed to mature tRNA sequences, and the remaining unmapped reads were aligned to precursor tRNA sequences, also allowing for one mismatch, with bowtie software. tsRNA expression was quantified by sequencing counts and normalized as counts per million (CPM) of total aligned reads. Differentially expressed genes were assessed based on the count values using the R package edgeR.

### ELISA

To measure TGFβ2 secretion, human ESCC cell lines (5 × 10^5^ cells per well) were cultured in 12‐well plates, and the supernatant was collected and centrifuged at 1000 × g for 10 min at 4 °C. Mouse serum was obtained from peripheral blood by centrifuging at 1000 × g for 20 min at 4 °C. For tumor interstitial fluid (TIF), 0.1 g of fresh tumor samples was cut into small pieces, washed with pre‐cold PBS, incubated in 0.5 mL PBS for 1 h at 37 °C, then centrifuged at 1000 rpm for 3 min, followed by centrifugation of the supernatants at 5000 rpm for 20 min at 4 °C.^[^ ^55^ ^]^ TGFβ2 concentration in cell culture supernatant was measured using a Human Transforming Growth Factor β2 (TGFβ2) ELISA Kit (mlbio, ml061149). The mouse serum specimens and TIF were used for examining TGFβ2 concentration by a Mouse Transforming Growth Factor β2 (TGFβ2) ELISA Kit (mlbio, ml037207).

### CUT&RUN Assay

5 × 10^4^ cells with *HNRNPAB* silence or control sequence were harvested and processed with the Hyperactive pG‐MNase CUT&RUN Assay Kit for PCR/qPCR (Vazyme, HD101) following the supplier's instructions. hnRNPAB antibody was used at 5 µg. DNA was purified using DNA spin columns and eluted in 50 µL buffer. Finally, binding of hnRNPAB onto the *TGFB2* promoter was quantified by RT‐qPCR, and the primers are shown in Table S7 (Supporting Information).

### Dual‐Luciferase Reporter Assay

*TGFB2* promoter sequence (2 kb upstream of the transcription start site) or its truncated or mutated forms were cloned into the pGL4‐promoter vector (Promega). KYSE150 cells were concurrently transfected with 400 ng of pGL4‐promoter vector containing TGFB2 promoter, 4 ng of pRL‐SV40 Renilla (Promega) luciferase reporter vector, and 250 ng of pcDNA3.1 vector or vector containing HNRNPAB cDNA for 48 h. The luciferase activities were determined by a Dual‐Luciferase Reporter Assay System (Promega), and the relative Fluc/Rluc activity was calculated by normalizing the activity of firefly luciferase to that of Renilla luciferase.

### Statistical Analysis

Student's *t*‐test was used to compare two means, and one‐way ANOVA with Dunnett's T3 multiple comparison test for multiple group comparisons. Fisher's exact test was used for independence between two categorical variables, and the Wilcoxon rank‐sum test was used for any independence test between a continuous variable and a binary categorical variable, when there was no covariate to adjust for. Univariate Cox regression analysis and log‐rank test were performed for survival analysis. Statistical analysis in this study was performed by using statistical software *R* (v. 4.3.1) or GraphPad Prism (version 8.3.0). Data were expressed as the mean ± SEM, with a sample size of *n* ≥ 3. All analyses with *p* < 0.05 were considered significant, and FDR was used for multiple comparisons. ^*^, *p *< 0.05; ^**^, *p *< 0.01; ^***^, *p *< 0.001; ns, *p *> 0.05.

### Ethics Approval and Consent to Participate

Experimental animal procedures were approved by the Animal Care and Animal Experiments Committee of Qilu Hospital of Shandong University. The tumor volumes in all experimental groups remained below 1500 mm^3^ until the endpoint of the experiment (Ethics Code: DWLL‐2023‐158). Human tumor tissue samples were obtained with informed consent, and the study was approved by the medical science research ethics committee of Qilu Hospital of Shandong University (Ethics Code: KYLL‐2022‐ZM‐721).

## Conflict of Interest

The authors declare no conflict of interest.

## Author Contributions

L.P. and X.Q. contributed equally to this work. Y.C. and L.P. conceptualized and supervised the study. L.P. and X.Q. performed phenotypic and functional experiments. L.P., X.Q., and J.W. conducted statistical and bioinformatics analyses. L.G., B.C., Y.W., and L.Z. were responsible for sample preparation, clinical data collection, and association analyses. H.L. and Q.S. provided technical support. Y.H. contributed to histopathological analyses. L.P. and X.Q. prepared the manuscript. All authors reviewed the manuscript.

## Supporting information



Supporting Information

Supporting Information

## Data Availability

All data needed to evaluate the conclusions in the paper are present in the paper and/or the Supplementary Materials. Sequencing data have been deposited in the Genome Sequence Archive in BIG Data Center (https://bigd.big.ac.cn/), Beijing Institute of Genomics, Chinese Academy of Sciences, under the accession number: HRA008538 and CRA028444. These data are accessible to all readers. The mass spectrometry proteomics data have been deposited to the ProteomeXchange Consortium via the PRIDE partner repository with the dataset identifier PXD057698.

## References

[1] D. Schadendorf , F. S. Hodi , C. Robert , J. S. Weber , K. Margolin , O. Hamid , D. Patt , T.‐T. Chen , D. M. Berman , J. D. Wolchok , J. Clin. Oncol. 2015, 33, 1889. 25667295 10.1200/JCO.2014.56.2736 PMC5089162

[2] K. M. Hargadon , C. E. Johnson , C. J. Williams , Int. Immunopharmacol. 2018, 62, 29. 29990692 10.1016/j.intimp.2018.06.001

[3] J. M. Pitt , M. Vétizou , R. Daillère , M. P. Roberti , T. Yamazaki , B. Routy , P. Lepage , I. G. Boneca , M. Chamaillard , G. Kroemer , L. Zitvogel , Immunity 2016, 44, 1255. 27332730 10.1016/j.immuni.2016.06.001

[4] F. Bray , M. Laversanne , H. Sung , J. Ferlay , R. L. Siegel , I. Soerjomataram , A. Jemal , CA Cancer J. Clin. 2024, 74, 229. 38572751 10.3322/caac.21834

[5] R. S. Zheng , R. Chen , B. F. Han , S. M. Wang , L. Li , K. X. Sun , H. M. Zeng , W. W. Wei , J. He , Zhonghua Zhong Liu Za Zhi 2022, 46, 221. 10.3760/cma.j.cn112152-20240119-00035 38468501

[6] L. B. Alexandrov , S. Nik‐Zainal , D. C. Wedge , S. A. J. R. Aparicio , S. Behjati , A. V. Biankin , G. R. Bignell , N. Bolli , A. Borg , A.‐L. Børresen‐Dale , S. Boyault , B. Burkhardt , A. P. Butler , C. Caldas , H. R. Davies , C. Desmedt , R. Eils , J. E. Eyfjörd , J. A. Foekens , M. Greaves , F. Hosoda , B. Hutter , T. Ilicic , S. Imbeaud , M. Imielinski , N. Jäger , D. T. W. Jones , D. Jones , S. Knappskog , M. Kool , et al., Nature 2013, 500, 415.23945592

[7] T. A. Chan , M. Yarchoan , E. Jaffee , C. Swanton , S. A. Quezada , A. Stenzinger , S. Peters , Ann. Oncol. 2019, 30, 44. 30395155 10.1093/annonc/mdy495 PMC6336005

[8] H. Luo , J. Lu , Y. Bai , T. Mao , J. Wang , Q. Fan , Y. Zhang , K. Zhao , Z. Chen , S. Gao , J. Li , Z. Fu , K. Gu , Z. Liu , L. Wu , X. Zhang , J. Feng , Z. Niu , Y. Ba , H. Zhang , Y. Liu , L. Zhang , X. Min , J. Huang , Y. Cheng , D. Wang , Y. Shen , Q. Yang , J. Zou , R.‐H. Xu , et al., JAMA, J. Am. Med. Assoc. 2021, 326, 916. 10.1001/jama.2021.12836 PMC8441593 34519801

[9] Z.‐X. Wang , C. Cui , J. Yao , Y. Zhang , M. Li , J. Feng , S. Yang , Y. Fan , J. Shi , X. Zhang , L. Shen , Y. Shu , C. Wang , T. Dai , T. Mao , L. Chen , Z. Guo , B. Liu , H. Pan , S. Cang , Y. Jiang , J. Wang , M. Ye , Z. Chen , D. Jiang , Q. Lin , W. Ren , J. Wang , L. Wu , Y. Xu , et al., Cancer Cell 2022, 40, 277. 35245446 10.1016/j.ccell.2022.02.007

[10] K. Kato , B. C. Cho , M. Takahashi , M. Okada , C.‐Y. Lin , K. Chin , S. Kadowaki , M.‐J. Ahn , Y. Hamamoto , Y. Doki , C.‐C. Yen , Y. Kubota , S.‐B. Kim , C.‐H. Hsu , E. Holtved , I. Xynos , M. Kodani , Y. Kitagawa , Lancet Oncol. 2019, 20, 1506. 31582355 10.1016/S1470-2045(19)30626-6

[11] Y. Zheng , Z. Chen , Y. Han , L. Han , X. Zou , B. Zhou , R. Hu , J. Hao , S. Bai , H. Xiao , W. V. Li , A. Bueker , Y. Ma , G. Xie , J. Yang , S. Chen , H. Li , J. Cao , L. Shen , Nat. Commun. 2020, 11, 6268. 33293583 10.1038/s41467-020-20019-0 PMC7722722

[12] S. A. Lasser , F. G. Ozbay Kurt , I. Arkhypov , J. Utikal , V. Umansky , Nat. Rev. Clin. Oncol. 2024, 21, 147. 38191922 10.1038/s41571-023-00846-y

[13] F. Veglia , E. Sanseviero , D. I Gabrilovich , Nat. Rev. Immunol. 2021, 21, 485. 33526920 10.1038/s41577-020-00490-y PMC7849958

[14] S. Hegde , A. M. Leader , M. Merad , Immunity 2021, 54, 875. 33979585 10.1016/j.immuni.2021.04.004 PMC8709560

[15] V. Bronte , S. Brandau , S.‐H. Chen , M. P. Colombo , A. B. Frey , T. F. Greten , S. Mandruzzato , P. J. Murray , A. Ochoa , S. Ostrand‐Rosenberg , P. C. Rodriguez , A. Sica , V. Umansky , R. H. Vonderheide , D. I. Gabrilovich , Nat. Commun. 2016, 7, 12150. 27381735 10.1038/ncomms12150 PMC4935811

[16] H. Liu , X. Zeng , X. Ren , Y. Zhang , M. Huang , L. Tan , Z. Dai , J. Lai , W. Xie , Z. Chen , S. Peng , L. Xu , S. Chen , S. Shen , M. Kuang , S. Lin , Gut 2023, 72, 1555. 36283801 10.1136/gutjnl-2022-327230

[17] F. Veglia , M. Perego , D. Gabrilovich , Nat. Immunol. 2018, 19, 108. 29348500 10.1038/s41590-017-0022-x PMC5854158

[18] J. Chen , X. Liu , Y. Zou , J. Gong , Z. Ge , X. Lin , W. Zhang , H. Huang , J. Zhao , P. E. Saw , Y. Lu , H. Hu , E. Song , Proc. Natl. Acad. Sci. USA 2024, 121, 2306776121. 10.1073/pnas.2306776121 PMC11098111 38709933

[19] H. Mohammadpour , C. R. MacDonald , G. Qiao , M. Chen , B. Dong , B. L. Hylander , P. L. McCarthy , S. I. Abrams , E. A. Repasky , J. Clin. Invest. 2019, 129, 5537. 31566578 10.1172/JCI129502 PMC6877316

[20] X. Zeng , G. Liao , S. Li , H. Liu , X. Zhao , S. Li , K. Lei , S. Zhu , Z. Chen , Y. Zhao , X. Ren , T. Su , A. S.‐L. Cheng , S. Peng , S. Lin , J. Wang , S. Chen , M. Kuang , Hepatology 2023, 77, 1122. 35598182 10.1002/hep.32585

[21] L. Wang , H. Wang , M. Zhu , X. Ni , L. Sun , W. Wang , J. Xie , Y. Li , Y. Xu , R. Wang , S. Han , P. Zhang , J. Peng , M. Hou , Y. Hou , Blood 2024, 144, 99. 38574321 10.1182/blood.2023022738

[22] M. Bodogai , K. Moritoh , C. Lee‐Chang , C. M. Hollander , C. A. Sherman‐Baust , R. P. Wersto , Y. Araki , I. Miyoshi , L. Yang , G. Trinchieri , A. Biragyn , Cancer Res. 2015, 75, 3456. 26183924 10.1158/0008-5472.CAN-14-3077 PMC4558269

[23] P. Cao , Z. Sun , F. Zhang , J. Zhang , X. Zheng , B. Yu , Y. Zhao , W. Wang , W. Wang , Front. Immunol. 2022, 13, 919674. 35874674 10.3389/fimmu.2022.919674 PMC9300822

[24] S. Muthukumar , C.‐T. Li , R.‐J. Liu , C. Bellodi , Nat. Rev. Mol. Cell Biol. 2024, 25, 359. 38182846 10.1038/s41580-023-00690-z

[25] M. Fu , J. Gu , M. Wang , J. Zhang , Y. Chen , P. Jiang , T. Zhu , X. Zhang , Mol. Cancer 2023, 22, 30. 36782290 10.1186/s12943-023-01739-5 PMC9926655

[26] R. L. Maute , C. Schneider , P. Sumazin , A. Holmes , A. Califano , K. Basso , R. Dalla‐Favera , Proc. Natl. Acad. Sci. USA 2013, 110, 1404. 23297232 10.1073/pnas.1206761110 PMC3557069

[27] B. Huang , H. Yang , X. Cheng , D. Wang , S. Fu , W. Shen , Q. Zhang , L. Zhang , Z. Xue , Y. Li , Y. Da , Q. Yang , Z. Li , L. Liu , L. Qiao , Y. Kong , Z. Yao , P. Zhao , M. Li , R. Zhang , Cancer Res. 2017, 77, 3194. 28446464 10.1158/0008-5472.CAN-16-3146

[28] H. K. Kim , G. Fuchs , S. Wang , W. Wei , Y. Zhang , H. Park , B. Roy‐Chaudhuri , P. Li , J. Xu , K. Chu , F. Zhang , M.‐S. Chua , S. So , Q. C. Zhang , P. Sarnow , M. A. Kay , Nature 2017, 552, 57. 29186115 10.1038/nature25005 PMC6066594

[29] N. Guzzi , M. Ciesla , P. C. T. Ngoc , S. Lang , S. Arora , M. Dimitriou , K. Pimková , M. N. E. Sommarin , R. Munita , M. Lubas , Y. Lim , K. Okuyama , S. Soneji , G. Karlsson , J. Hansson , G. Jönsson , A. H. Lund , M. Sigvardsson , E. Hellström‐Lindberg , A. C. Hsieh , C. Bellodi , Cell 2018, 173, 1204. 29628141 10.1016/j.cell.2018.03.008

[30] H. Goodarzi , X. Liu , H. C. B. Nguyen , S. Zhang , L. Fish , S. F. Tavazoie , Cell 2015, 161, 790. 25957686 10.1016/j.cell.2015.02.053 PMC4457382

[31] L. Pan , X. Huang , Z.‐X. Liu , Y. Ye , R. Li , J. Zhang , G. Wu , R. Bai , L. Zhuang , L. Wei , M. Li , Y. Zheng , J. Su , J. Deng , S. Deng , L. Zeng , S. Zhang , C. Wu , X. Che , C. Wang , R. Chen , D. Lin , J. Zheng , J. Clin. Invest. 2021, 131, 148130. 10.1172/JCI148130 PMC8592549 34779408

[32] N. T. Chiou , R. Kageyama , K. M. Ansel , Cell Rep. 2018, 25, 3356. 30566862 10.1016/j.celrep.2018.11.073 PMC6392044

[33] T. Yue , X. Zhan , D. Zhang , R. Jain , K.‐W. Wang , J. H. Choi , T. Misawa , L. Su , J. Quan , S. Hildebrand , D. Xu , X. Li , E. Turer , L. Sun , E. M. Y. Moresco , B. Beutler , Science 2021, 372, aba4220. 10.1126/science.aba4220 PMC8442736 33986151

[34] V. Pliatsika , P. Loher , R. Magee , A. G. Telonis , E. Londin , M. Shigematsu , Y. Kirino , I. Rigoutsos , Nucleic Acids Res. 2018, 46, D152. 29186503 10.1093/nar/gkx1075 PMC5753276

[35] S. Hatakeyama , Nat. Rev. Cancer 2011, 11, 792. 21979307 10.1038/nrc3139

[36] Q. Ma , H. Jiang , L. Ma , G. Zhao , Q. Xu , D. Guo , N. He , H. Liu , Z. Meng , J. Liu , L. Zhu , Q. Lin , X. Wu , M. Li , S. Luo , J. Fang , Z. Lu , Proc. Natl. Acad. Sci. USA 2023, 120, 2209435120. 10.1073/pnas.2209435120 PMC10104498 37011206

[37] Z.‐J. Zhou , Z. Dai , S.‐L. Zhou , Z.‐Q. Hu , Q. Chen , Y.‐M. Zhao , Y.‐H. Shi , Q. Gao , W.‐Z. Wu , S.‐J. Qiu , J. Zhou , J. Fan , Cancer Res. 2014, 74, 2750. 24638979 10.1158/0008-5472.CAN-13-2509

[38] C. D. Venkov , A. J. Link , J. L. Jennings , D. Plieth , T. Inoue , K. Nagai , C. Xu , Y. N. Dimitrova , F. J. Rauscher , E. G. Neilson , J. Clin. Invest. 2007, 117, 482. 17273560 10.1172/JCI29544 PMC1783826

[39] W. Li , W. Shen , B. Zhang , K. Tian , Y. Li , L. Mu , Z. Luo , X. Zhong , X. Wu , Y. Liu , Y. Zhou , Protein Cell 2020, 11, 161. 31317506 10.1007/s13238-019-0650-z PMC7026249

[40] A. Berson , S. Barbash , G. Shaltiel , Y. Goll , G. Hanin , D. S. Greenberg , M. Ketzef , A. J. Becker , A. Friedman , H. Soreq , EMBO Mol. Med. 2012, 4, 730. 22628224 10.1002/emmm.201100995 PMC3494073

[41] M. G. Lechner , C. Megiel , S. M. Russell , B. Bingham , N. Arger , T. Woo , A. L. Epstein , J. Transl. Med. 2011, 9, 90. 21658270 10.1186/1479-5876-9-90 PMC3128058

[42] E. N. M. Nolte‐'t Hoen , H. P. J. Buermans , M. Waasdorp , W. Stoorvogel , M. H. M. Wauben , P. A. C. Hoen , Nucleic Acids Res. 2012, 40, 9272. 22821563 10.1093/nar/gks658 PMC3467056

[43] M. Yang , Y. Mo , D. Ren , S. Liu , Z. Zeng , W. Xiong , Mol. Cancer 2023, 22, 32. 36797764 10.1186/s12943-023-01742-w PMC9933334

[44] Y. Doki , J. A. Ajani , K. Kato , J. Xu , L. Wyrwicz , S. Motoyama , T. Ogata , H. Kawakami , C.‐H. Hsu , A. Adenis , F. El Hajbi , M. Di Bartolomeo , M. I. Braghiroli , E. Holtved , S. A. Ostoich , H. R. Kim , M. Ueno , W. Mansoor , W.‐C. Yang , T. Liu , J. Bridgewater , T. Makino , I. Xynos , X. Liu , M. Lei , K. Kondo , A. Patel , J. Gricar , I. Chau , Y. Kitagawa , N. Engl. J. Med. 2022, 386, 449.35108470

[45] M. Okada , K. Kato , B. C. Cho , M. Takahashi , C.‐Y. Lin , K. Chin , S. Kadowaki , M.‐J. Ahn , Y. Hamamoto , Y. Doki , C.‐C. Yen , Y. Kubota , S.‐B. Kim , C.‐H. Hsu , E. Holtved , I. Xynos , Y. Matsumura , A. Takazawa , Y. Kitagawa , Clin. Cancer Res. 2022, 28, 3277. 35294546 10.1158/1078-0432.CCR-21-0985 PMC9662935

[46] L. Sabra , L. Katie , Nat. Rev. Immunol. 2016, 16, 626.27546235

[47] Y. Ye , Y. Jing , L. Li , G. B. Mills , L. Diao , H. Liu , L. Han , Nat. Commun. 2020, 11, 1779. 32286310 10.1038/s41467-020-15679-x PMC7156379

[48] T. Huang , J. Yang , B. Liu , L. Fu , Cancer Commun. 2020, 40, 316. 10.1002/cac2.12066 PMC7365451 32510874

[49] T.‐X. Huang , X.‐Y. Tan , H.‐S. Huang , Y.‐T. Li , B.‐L. Liu , K.‐S. Liu , X. Chen , Z. Chen , X.‐Y. Guan , C. Zou , L. Fu , Gut 2022, 71, 333. 33692094 10.1136/gutjnl-2020-322924 PMC8762012

[50] H. Wu , X. Leng , Q. Liu , T. Mao , T. Jiang , Y. Liu , F. Li , C. Cao , J. Fan , L. Chen , Y. Chen , Q. Yao , S. Lu , R. Liang , L. Hu , M. Liu , Y. Wan , Z. Li , J. Peng , Q. Luo , H. Zhou , J. Yin , K. Xu , M. Lan , X. Peng , H. Lan , G. Li , Y. Han , X. Zhang , Z.‐X. J. Xiao , et al., Cancer Res. 2023, 83, 3131. 37433041 10.1158/0008-5472.CAN-22-2593

[51] L. Li , R. Zhu , H. Zhou , C.‐P. Cui , X. Yu , Y. Liu , Y. Yin , Y. Li , R. Feng , J. P. Katz , Y. Zhao , Y. Zhang , L. Zhang , Z. Liu , Adv. Sci. 2023, 10, 2207458. 10.1002/advs.202207458 PMC10238178 37038094

[52] C. Wilks , M. S. Cline , E. Weiler , M. Diehkans , B. Craft , C. Martin , D. Murphy , H. Pierce , J. Black , D. Nelson , B. Litzinger , T. Hatton , L. Maltbie , M. Ainsworth , P. Allen , L. Rosewood , E. Mitchell , B. Smith , J. Warner , J. Groboske , H. Telc , D. Wilson , B. Sanford , H. Schmidt , D. Haussler , D. Maltbie , Database 2014, 2014, bau093. 25267794 10.1093/database/bau093 PMC4178372

[53] P. Loher , A. G. Telonis , I. Rigoutsos , Sci. Rep. 2017, 7, 41184. 28220888 10.1038/srep41184 PMC5318995

[54] Y.‐M. He , X. Li , M. Perego , Y. Nefedova , A. V. Kossenkov , E. A. Jensen , V. Kagan , Y.‐F. Liu , S.‐Y. Fu , Q.‐J. Ye , Y.‐H. Zhou , L. Wei , D. I. Gabrilovich , J. Zhou , Nat. Med. 2018, 24, 224. 29334374 10.1038/nm.4467 PMC5803434

[55] D.‐S. Kim , H. Dastidar , C. Zhang , F. J. Zemp , K. Lau , M. Ernst , A. Rakic , S. Sikdar , J. Rajwani , V. Naumenko , D. R. Balce , B. W. Ewanchuk , P. Tailor , R. M. Yates , C. Jenne , C. Gafuik , D. J. Mahoney , Nat. Commun. 2017, 8, 344. 28839138 10.1038/s41467-017-00324-x PMC5570934

