# Supplementary material for: tRNA‐Derived Fragment tRF‐22 Promotes Immunosuppression by Inhibiting HnRNPAB Ubiquitination in Esophageal Squamous Cell Carcinoma
Source: Adv Sci (Weinh). 2025 Oct 27;13(1):e05806. doi: 10.1002/advs.202505806 (PMC12767015; doi:10.1002/advs.202505806)
Supplement: Supplementary file 1 — Supporting Information [file ADVS-13-e05806-s001.pdf]

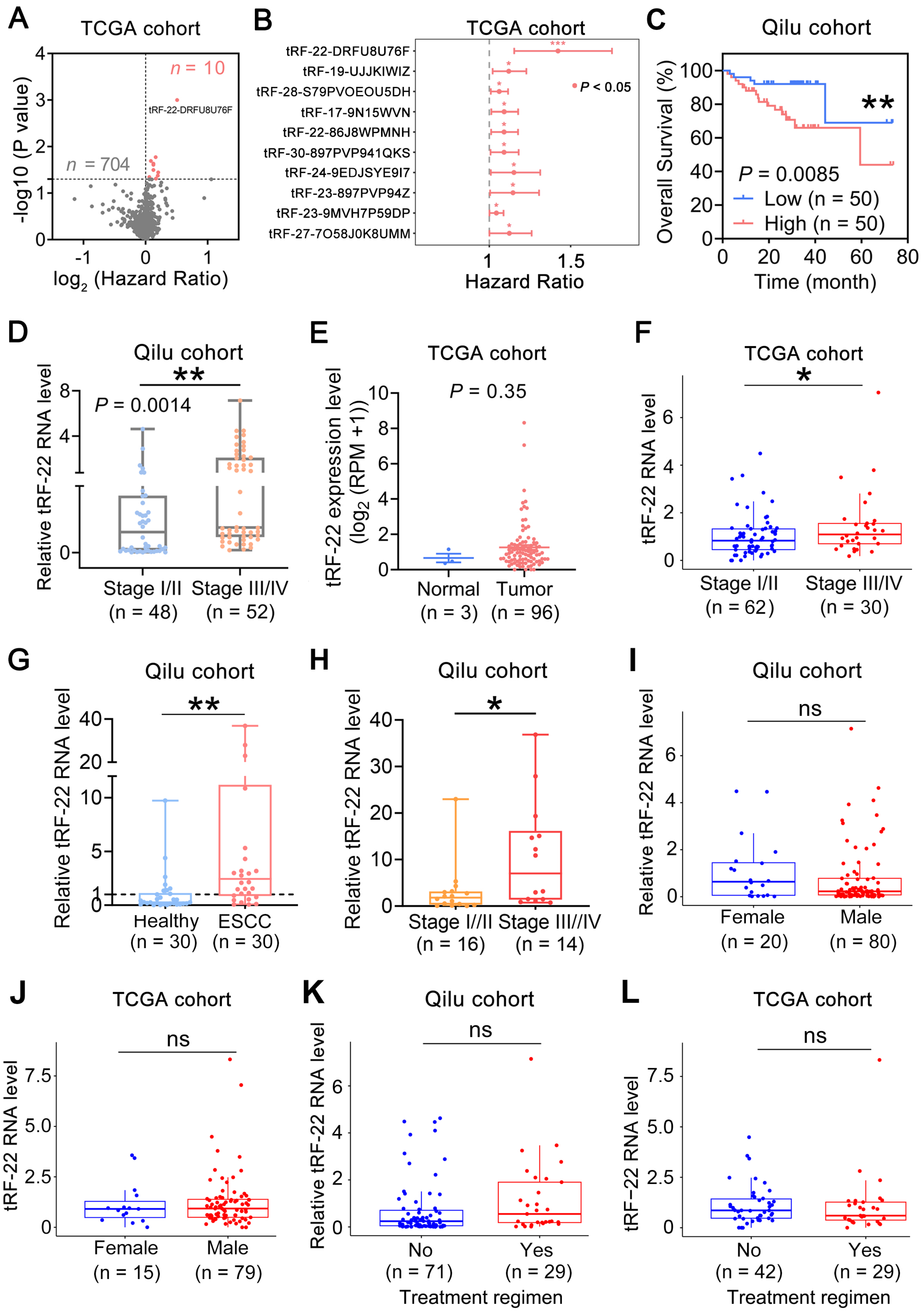
A
TCGA cohort
n = 10
tRF-22-DRFU8U76F
n = 704
-log10 (P value)
log2 (Hazard Ratio)
B
TCGA cohort
tRF-22-DRFU8U76F
tRF-19-UJJKIWIZ
tRF-28-S79PVOEOU5DH
tRF-17-9N15WVN
tRF-22-86J8WPMNH
tRF-30-897PVP941QKS
tRF-24-9EDJSYE9I7
tRF-23-897PVP94Z
tRF-23-9MVH7P59DP
tRF-27-7O58J0K8UMM
P < 0.05
Hazard Ratio
C
Qilu cohort
Overall Survival (%)
P = 0.0085
Low (n = 50)
High (n = 50)
Time (month)
D
Qilu cohort
P = 0.0014
Relative tRF-22 RNA level
Stage I/II (n = 48)
Stage III/IV (n = 52)
E
TCGA cohort
P = 0.35
tRF-22 expression level (log2 (RPM +1))
Normal (n = 3)
Tumor (n = 96)
F
TCGA cohort
tRF-22 RNA level
Stage I/II (n = 62)
Stage III/IV (n = 30)
G
Qilu cohort
Relative tRF-22 RNA level
Healthy (n = 30)
ESCC (n = 30)
H
Qilu cohort
Relative tRF-22 RNA level
Stage I//II (n = 16)
Stage III//IV (n = 14)
I
Qilu cohort
ns
Relative tRF-22 RNA level
Female (n = 20)
Male (n = 80)
J
TCGA cohort
ns
tRF-22 RNA level
Female (n = 15)
Male (n = 79)
K
Qilu cohort
ns
Relative tRF-22 RNA level
No (n = 71)
Yes (n = 29)
Treatment regimen
L
TCGA cohort
ns
tRF-22 RNA level
No (n = 42)
Yes (n = 29)
Treatment regimen

Supplementary Figure S1. *tRF-22* identification and its clinical relevance, related to Figure 1.

(***A***) Volcano plot of tRFs associated with survival time in the TCGA ESCC patients. Pink dots represent *P* values below 0.05, whereas gray dots represent *P* values of 0.05 or higher. Univariate Cox hazard analysis was applied. (***B***) Associations of the expression levels of ten tRFs and patients' survival time in patients from the TCGA ESCC cohort. Univariate Cox hazard analysis was applied. *, $P < 0.05$; ***, $P < 0.001$. (***C***) Kaplan-Meier estimates of patients' overall survival time in the Qilu cohort according to *tRF-22* levels in ESCC. *P* values were determined by log-rank test. **, $P < 0.01$. (***D***) Relative *tRF-22* expression in ESCC tissues across tumor stages in the Qilu cohort by RT-qPCR. (***E***) *tRF-22* levels in ESCC tissues and adjacent normal tissues in the TCGA cohort based on small RNA-seq. (***F***) *tRF-22* levels in ESCC tissues across tumor stages in the TCGA cohort. (***G***) Relative *tRF-22* expression in the serum of 30 healthy persons and 30 ESCC patients in the Qilu cohort. (***H***) Relative serum *tRF-22* levels in 30 ESCC patients across tumor stages in the Qilu cohort. (***I***) Relative *tRF-22* expression in ESCC tissues across sexes in the Qilu cohort. (***J***) *tRF-22* levels in ESCC tissues across sexes in the TCGA cohort. (***K***) Relative *tRF-22* expression in tumor tissues of ESCC patients from the Qilu cohort receiving surgery alone (No) or surgery combined with other antitumor therapies, like radiotherapy and chemotherapy (Yes). (***L***) *tRF-22* levels in tumor tissues of ESCC patients from the TCGA cohort receiving surgery alone (No) or surgery combined with radiotherapy (Yes). In the TCGA cohort of 96 patients, stage information was unavailable for 4 patients (***F***), sex information was missing for 2 patients (***J***) and treatment regimens were not reported for 25 patients (***L***). (***D-L***) *, $P < 0.05$; **, $P < 0.01$ and ns, not significant by t test.

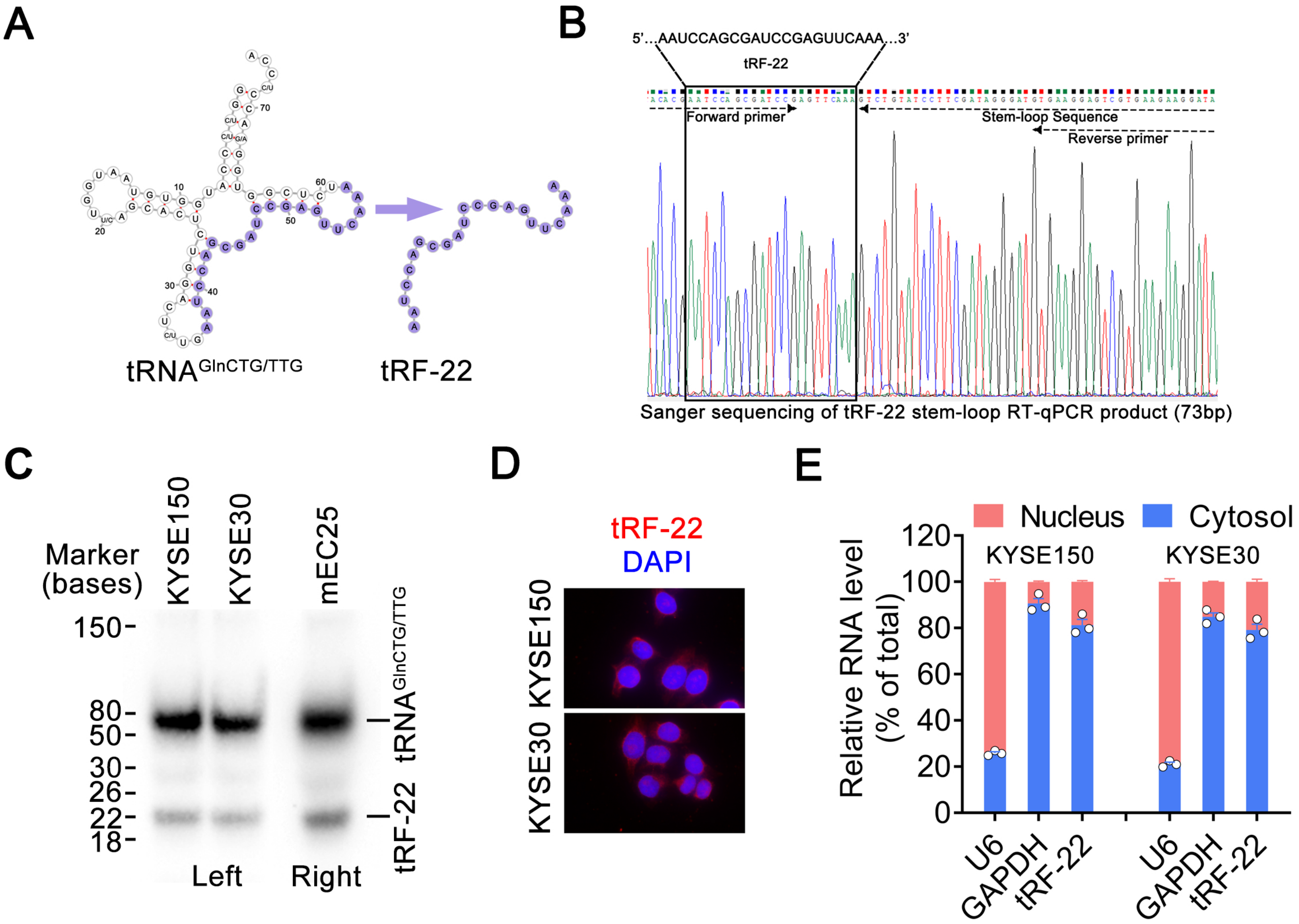
A
tRNA GlnCTG/TTG
tRF-22
B
5'...AAUCCAGCGAUCCGAGUUCAAA...3'
tRF-22
Forward primer
Stem-loop Sequence
Reverse primer
Sanger sequencing of tRF-22 stem-loop RT-qPCR product (73bp)
C
Marker (bases)
KYSE150
KYSE30
mEC25
150
80
50
30
26
22
18
tRNA GlnCTG/TTG
tRF-22
Left
Right
D
tRF-22
DAPI
KYSE150
KYSE30
E
Nucleus
Cytosol
KYSE150
KYSE30
Relative RNA level (% of total)
U6
GAPDH
tRF-22

Supplementary Figure S2. Characterization of *tRF-22*, related to Figure 1.

(***A***) Diagram and sequence of *tRF-22* derived from mature *tRNA*$^{GlnCTG/TTG}$. (***B***) Sanger sequencing of stem-loop production of RT-qPCR. The sequence within the frame is *tRF-22* and arrows indicate primers. (***C***) Representative images of northern blot of *tRF-22* in ESCC cells by urea–polyacrylamide gel electrophoresis. (***D*** and ***E***) Analysis of subcellular localization by RNA FISH (***D***) or RT-qPCR (***E***) shows *tRF-22* is mainly located in the cytoplasm of ESCC cells. *U6* and *GAPDH* are respectively nuclear and cytoplasmic markers. (***E***) Data presented as mean ± SEM. All experiments were performed at least three independent times.

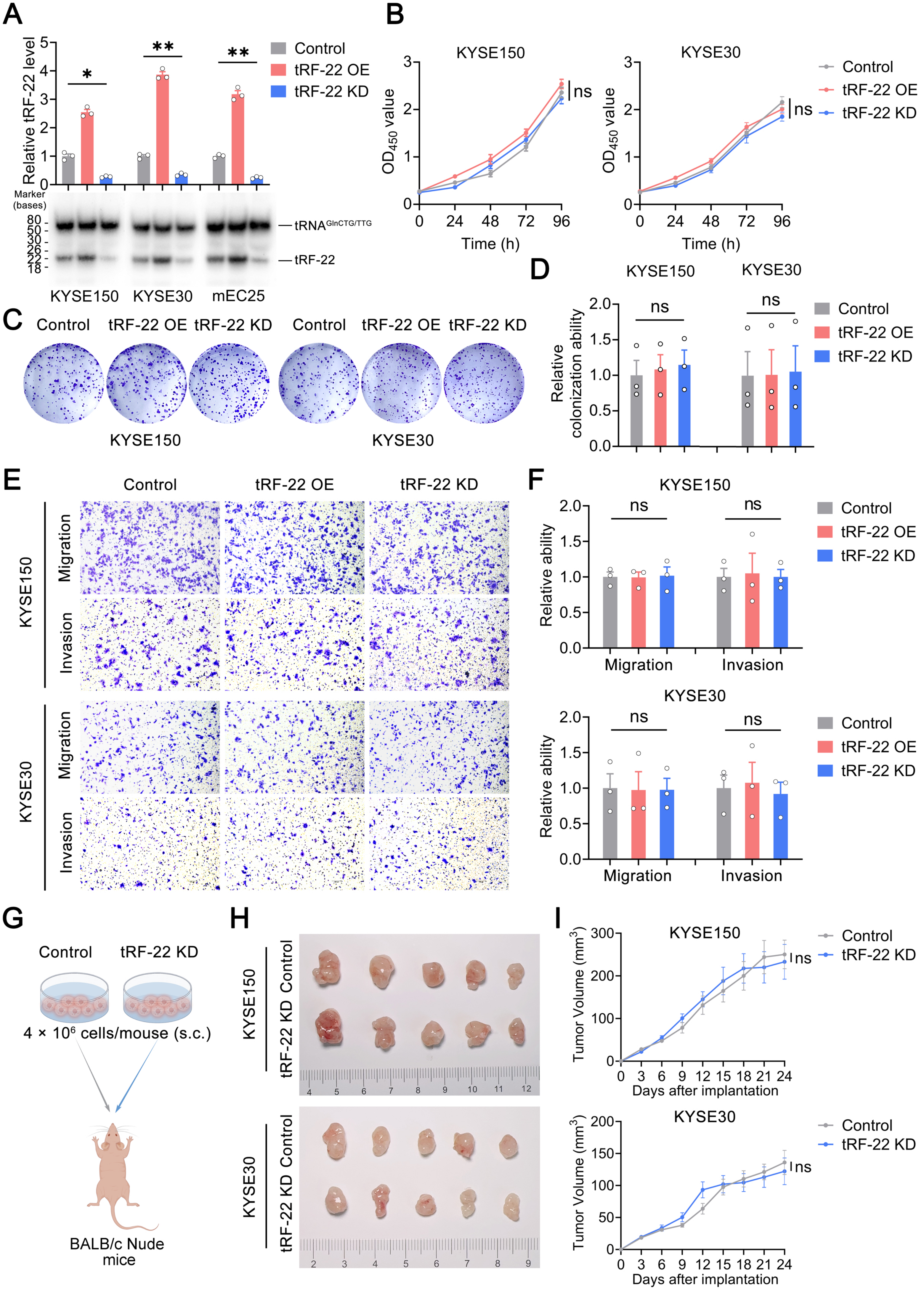

A
Relative tRF-22 level
Control
tRF-22 OE
tRF-22 KD
Marker (bases)
tRNA GlnCTG/TTG
tRF-22
KYSE150
KYSE30
mEC25
B
KYSE150
KYSE30
OD450 value
Time (h)
ns
C
Control
tRF-22 OE
tRF-22 KD
KYSE150
KYSE30
D
Relative colonization ability
E
Migration
Invasion
F
Relative ability
G
4 × 10^6 cells/mouse (s.c.)
BALB/c Nude mice
H
I
Tumor Volume (mm^3)
Days after implantation

Supplementary Figure S3. *tRF-22* does not directly influence the malignant phenotypes of ESCC cells, related to Figure 1.

(***A***) Cells with *tRF-22* overexpression (*tRF-22* OE) or silence (*tRF-22* KD) were subjected to determine relative *tRF-22* levels by RT-qPCR (*upper panel, n* = 3) and *tRF-22* and mature $tRNA^{GlnCTG/TTG}$ levels by northern blot (*lower panel*). (***B-F***) Effects of *tRF-22* overexpression or silence on ESCC cell proliferation (***B***), colony formation (***C*** and ***D***) and migration and invasion (***E*** and ***F***). (***G***) Schematic of the experiment of human ESCC cells in nude mice. (***H*** and ***I***) Effects of *tRF-22* expression changes on tumor burdens in nude mice with subcutaneously transplanted human ESCC cells (*n* = 5 per group). (***A, B, D, F***) Data presented as mean ± SEM, n = 3. *, $P < 0.05$; **, $P < 0.01$ and ns, not significant by Brown-Forsythe ANOVA with Dunnett's T3 multiple comparison test. (***I***) Data presented as mean ± SEM. ns, not significant by t test. All experiments were performed at least three independent times.

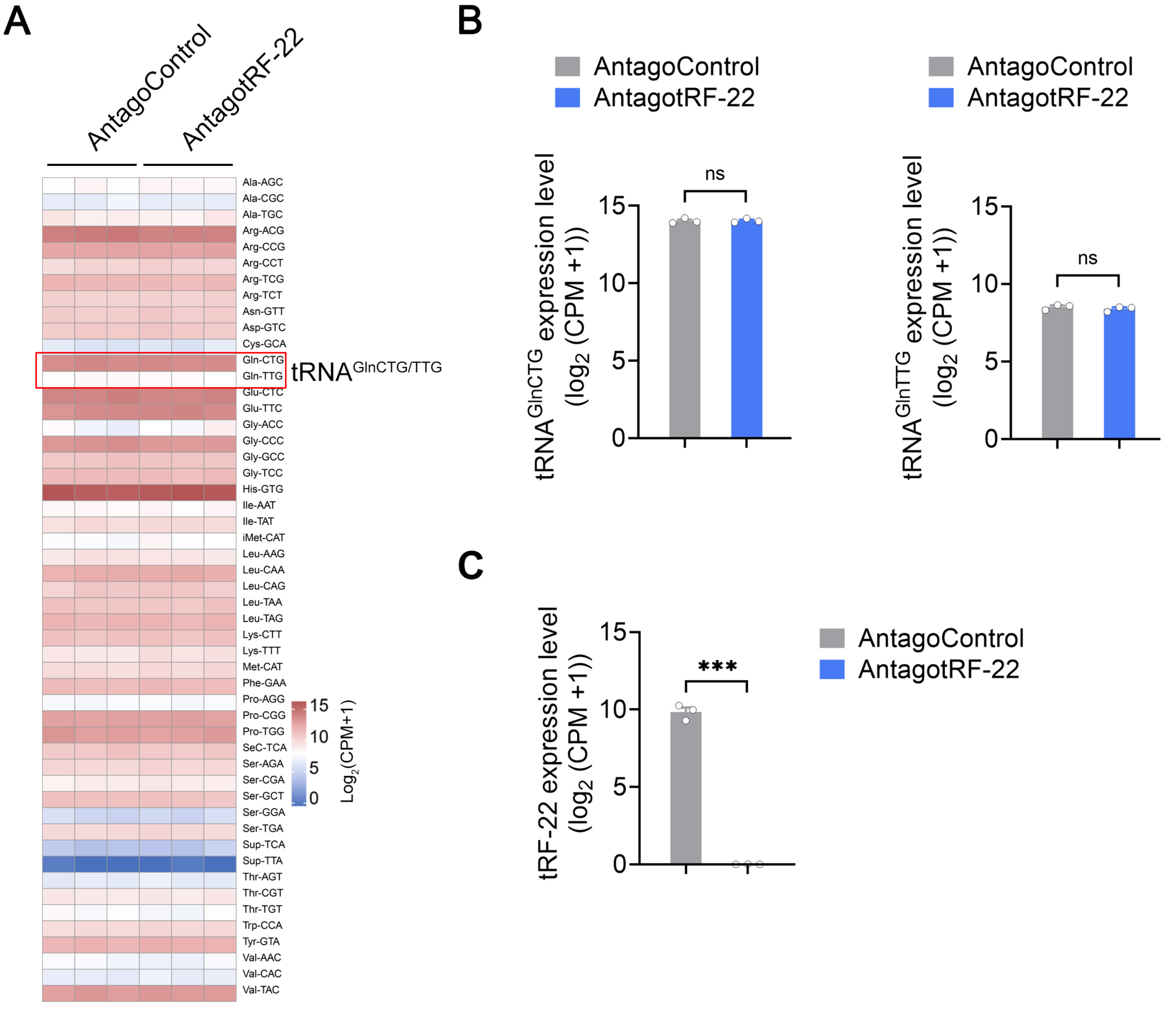

A
AntagoControl
AntagotRF-22
Ala-AGC
Ala-CGC
Ala-TGC
Arg-ACG
Arg-CCG
Arg-CCT
Arg-TCG
Arg-TCT
Asn-GTT
Asp-GTC
Cys-GCA
Gln-CTG
Gln-TTG
tRNA^GlnCTG/TTG
Glu-CTC
Glu-TTC
Gly-ACC
Gly-CCC
Gly-GCC
Gly-TCC
His-GTG
Ile-AAT
Ile-TAT
iMet-CAT
Leu-AAG
Leu-CAA
Leu-CAG
Leu-TAA
Leu-TAG
Lys-CTT
Lys-TTT
Met-CAT
Phe-GAA
Pro-AGG
Pro-CGG
Pro-TGG
SeC-TCA
Ser-AGA
Ser-CGA
Ser-GCT
Ser-GGA
Ser-TGA
Sup-TCA
Sup-TTA
Thr-AGT
Thr-CGT
Thr-TGT
Trp-CCA
Tyr-GTA
Val-AAC
Val-CAC
Val-TAC
15
10
5
0
Log2(CPM+1)
B
AntagoControl
AntagotRF-22
tRNA^GlnCTG expression level (log2 (CPM +1))
ns
15
10
5
0
AntagoControl
AntagotRF-22
tRNA^GlnTTG expression level (log2 (CPM +1))
ns
15
10
5
0
C
tRF-22 expression level (log2 (CPM +1))
***
15
10
5
0
AntagoControl
AntagotRF-22

Supplementary Figure S4. Analyses of tsRNA-seq and tRNA-seq for *tRF-22* expression and global tRNAs abundance, related to Figure 1.

(***A***) Heatmap showing no differences in the levels of mature tRNAs ($\log_2$(CPM+1)) in mEC25 cells with indicated treatments measured by tRNA-seq. Rows represent individual tRNA isoacceptors and columns represent independent replicates (n = 3). (***B***) No significant differences in mature $tRNA^{GlnCTG/TTG}$ levels between antagoControl and antagotRF-22 treatment groups. Data presented as mean ± SEM. ns, not significant by t test. (***C***) Significant reduction of *tRF-22* levels in mEC25 cells following antagotRF-22 treatment compared to antagoControl. Data presented as mean ± SEM. ***, $P < 0.001$ by t test.

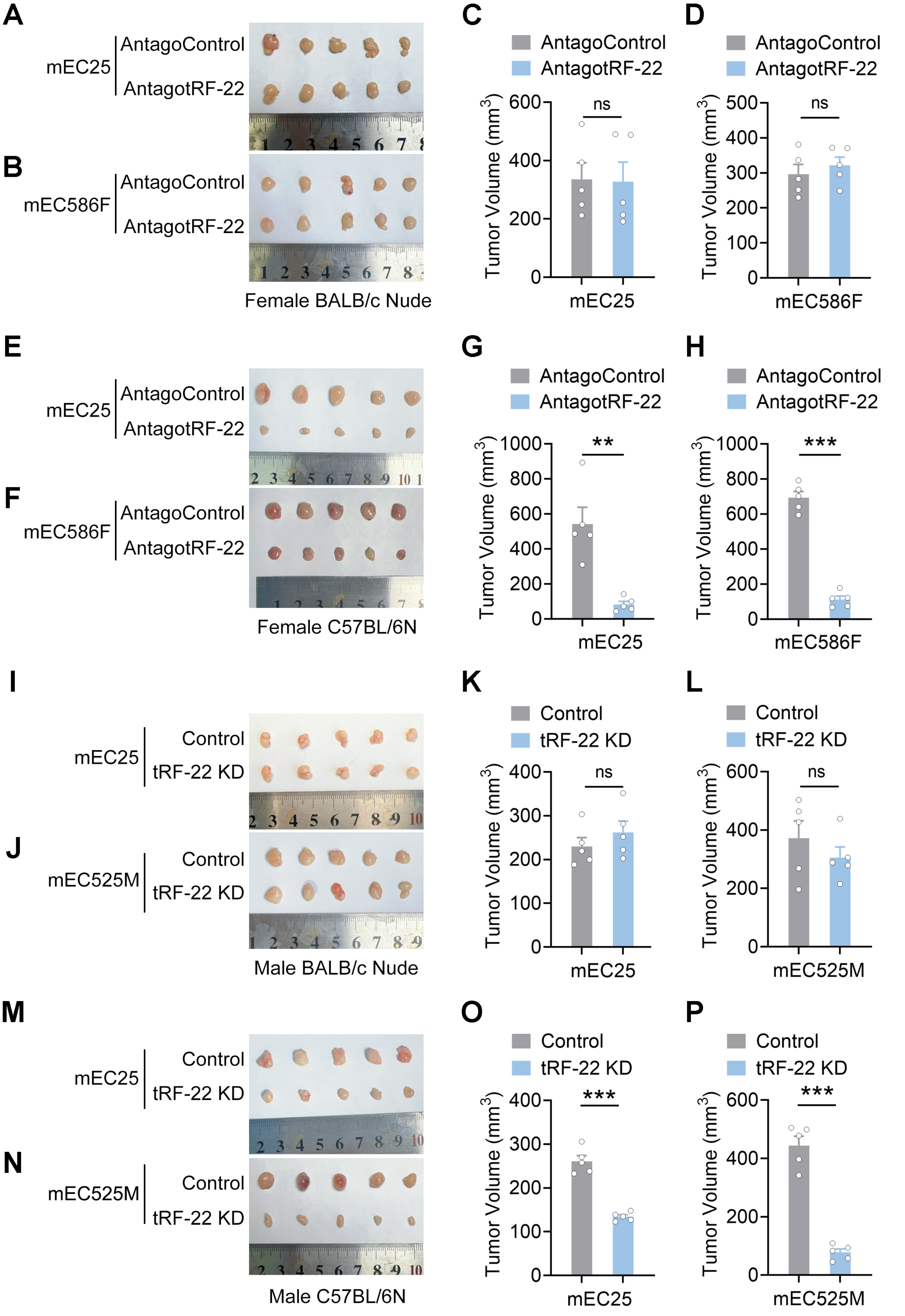

A
mEC25
AntagoControl
AntagotRF-22
B
mEC586F
AntagoControl
AntagotRF-22
Female BALB/c Nude
C
AntagoControl
AntagotRF-22
Tumor Volume (mm3)
ns
mEC25
D
AntagoControl
AntagotRF-22
Tumor Volume (mm3)
ns
mEC586F
E
mEC25
AntagoControl
AntagotRF-22
F
mEC586F
AntagoControl
AntagotRF-22
Female C57BL/6N
G
AntagoControl
AntagotRF-22
Tumor Volume (mm3)
**
mEC25
H
AntagoControl
AntagotRF-22
Tumor Volume (mm3)
***
mEC586F
I
mEC25
Control
tRF-22 KD
J
mEC525M
Control
tRF-22 KD
Male BALB/c Nude
K
Control
tRF-22 KD
Tumor Volume (mm3)
ns
mEC25
L
Control
tRF-22 KD
Tumor Volume (mm3)
ns
mEC525M
M
mEC25
Control
tRF-22 KD
N
mEC525M
Control
tRF-22 KD
Male C57BL/6N
O
Control
tRF-22 KD
Tumor Volume (mm3)
***
mEC25
P
Control
tRF-22 KD
Tumor Volume (mm3)
***
mEC525M

Supplementary Figure S5. *tRF-22* expression changes affect tumor burdens in a sex-independent manner, related to Figure 1.

(***A-H***) Effects of antagotRF-22 treatment on tumor burdens in female BALB/c nude mice (***A-D***) and female C57BL/6N mice (***E-H***) with subcutaneously transplanted mEC25 and mEC586F cells (n = 5 per group). (***I-P***) Effects of *tRF-22* expression changes on tumor burdens in male BALB/c nude mice (***I-L***) and male C57BL/6N mice (***M-P***) with subcutaneously transplanted mEC25 and mEC535M cells (n = 5 per group). **(*C, D, G, H, K, L, O, P*)** Data presented as mean ± SEM. **, $P < 0.01$, ***, $P < 0.001$ and ns, not significant by t test.

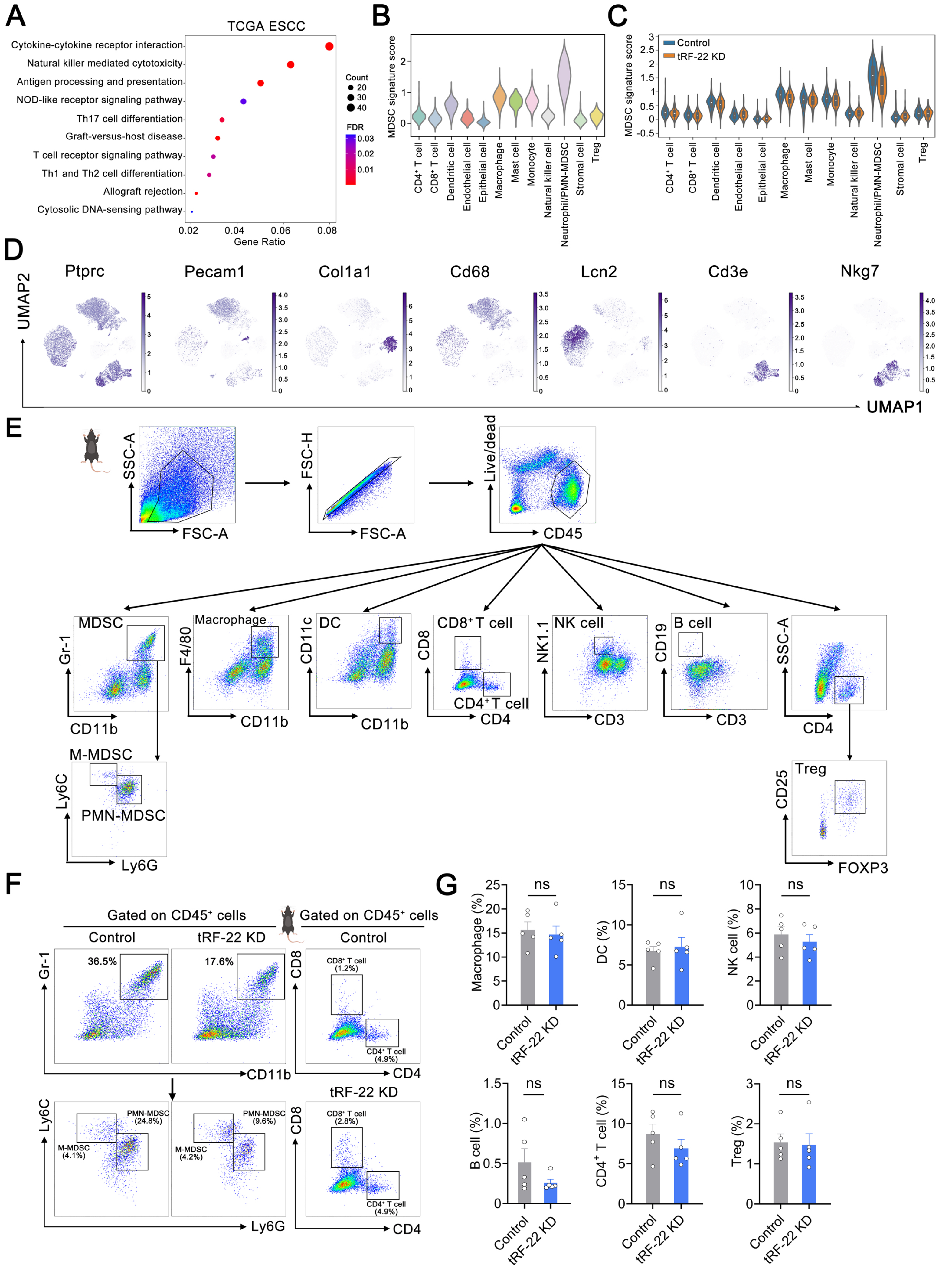
A
TCGA ESCC
Cytokine-cytokine receptor interaction
Natural killer mediated cytotoxicity
Antigen processing and presentation
NOD-like receptor signaling pathway
Th17 cell differentiation
Graft-versus-host disease
T cell receptor signaling pathway
Th1 and Th2 cell differentiation
Allograft rejection
Cytosolic DNA-sensing pathway
Gene Ratio
Count
FDR
B
MDSC signature score
C
Control
tRF-22 KD
CD4⁺ T cell
CD8⁺ T cell
Dendritic cell
Endothelial cell
Epithelial cell
Macrophage
Mast cell
Monocyte
Natural killer cell
Neutrophil/PMN-MDSC
Stromal cell
Treg
D
Ptprc
Pecam1
Col1a1
Cd68
Lcn2
Cd3e
Nkg7
UMAP1
UMAP2
E
SSC-A
FSC-A
FSC-H
Live/dead
CD45
MDSC
Gr-1
CD11b
Macrophage
F4/80
DC
CD11c
CD8⁺ T cell
CD8
CD4⁺ T cell
CD4
NK cell
NK1.1
CD3
B cell
CD19
M-MDSC
PMN-MDSC
Ly6C
Ly6G
Treg
CD25
FOXP3
F
Gated on CD45⁺ cells
Control
tRF-22 KD
36.5%
17.6%
PMN-MDSC (24.8%)
M-MDSC (4.1%)
PMN-MDSC (9.6%)
M-MDSC (4.2%)
CD8⁺ T cell (1.2%)
CD4⁺ T cell (4.9%)
CD8⁺ T cell (2.8%)
CD4⁺ T cell (4.9%)
G
Macrophage (%)
DC (%)
NK cell (%)
B cell (%)
CD4⁺ T cell (%)
Treg (%)
ns

Supplementary Figure S6. ***tRF-22*** **alters the infiltration of PMN-MDSCs and CD8$^+$ T cells within ESCC tumors, related to Figure 2.**

(***A***) KEGG enrichment analyses display immune related pathways associated with *tRF-22*. (***B*** and ***C***) MDSC signature enrichment score of indicated cell subclusters between Control and *tRF-22* KD groups. (***D***) UMAP plots showing the marker genes of total MDSCs and MDSC subclusters (PMN-MDSCs and M-MDSCs). (***E***) Gating strategy for different immune cells. (***F***) The representative pictures of PMN-MDSCs, M-MDSCs and CD8$^+$ T cells by flow cytometry, related to Figure 2H. (***G***) The percentages of Macrophage, dendritic cell, NK cell, B cell, CD4$^+$ T cell and Treg cell in tumors from Control or *tRF-22* KD groups were determined by flow cytometry (*n* = 5 per group). Data presented as mean ± SEM. ns, not significant by t test.

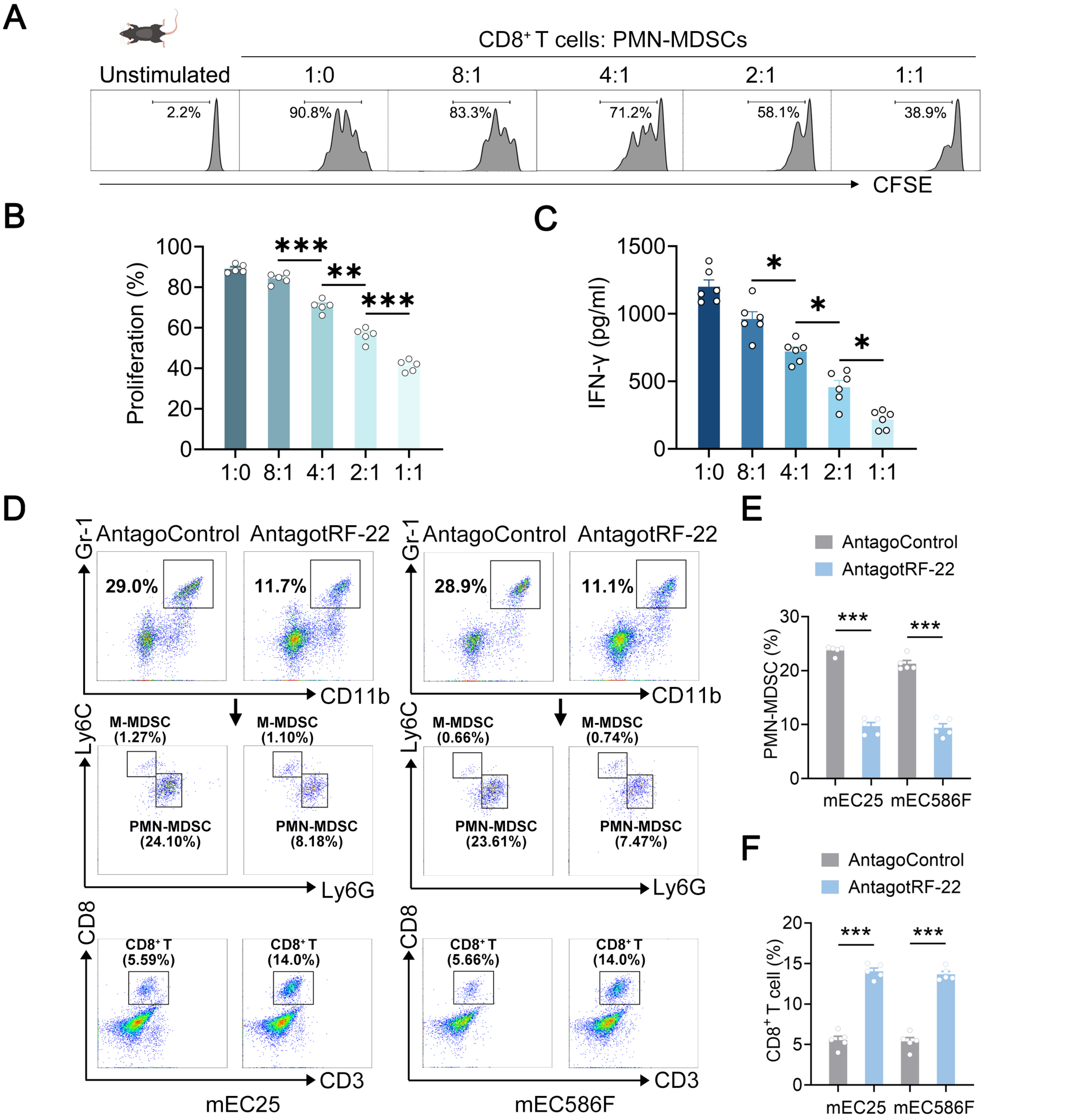
A
CD8+ T cells: PMN-MDSCs
Unstimulated
1:0
8:1
4:1
2:1
1:1
2.2%
90.8%
83.3%
71.2%
58.1%
38.9%
CFSE
B
Proliferation (%)
***
**
***
C
IFN-γ (pg/ml)
*
D
AntagoControl
AntagotRF-22
Gr-1
CD11b
29.0%
11.7%
28.9%
11.1%
Ly6C
Ly6G
M-MDSC (1.27%)
M-MDSC (1.10%)
M-MDSC (0.66%)
M-MDSC (0.74%)
PMN-MDSC (24.10%)
PMN-MDSC (8.18%)
PMN-MDSC (23.61%)
PMN-MDSC (7.47%)
CD8
CD3
CD8+ T (5.59%)
CD8+ T (14.0%)
CD8+ T (5.66%)
CD8+ T (14.0%)
mEC25
mEC586F
E
PMN-MDSC (%)
F
CD8+ T cell (%)

Supplementary Figure S7. PMN-MDSCs repress the proliferation and antitumor activity of $CD8^+$ T cells, related to Figure 2.

(***A-C***) PMN-MDSCs derived from ESCC tumors exhibit immunosuppressive function on the proliferation (***A*** and ***B***, $n = 5$) and function (***C***, $n = 6$) of $CD8^+$ T cell *in vitro*. (***D***) The representative pictures of PMN-MDSCs and $CD8^+$ T cells by flow cytometry, related to Supplementary Figure S5E-H. (***E*** and ***F***) The percentages of PMN-MDSCs and $CD8^+$ T cells in tumors from antagoControl or antagotRF-22 treatment group were determined by flow cytometry ($n = 5$ per group). (***B*** and ***C***) Data presented as mean ± SEM. *, $P < 0.05$; **, $P < 0.01$ and ***, $P < 0.001$ by Brown-Forsythe ANOVA with Dunnett's T3 multiple comparison test. (***E*** and ***F***) Data presented as mean ± SEM. ***, $P < 0.001$ by t-test.

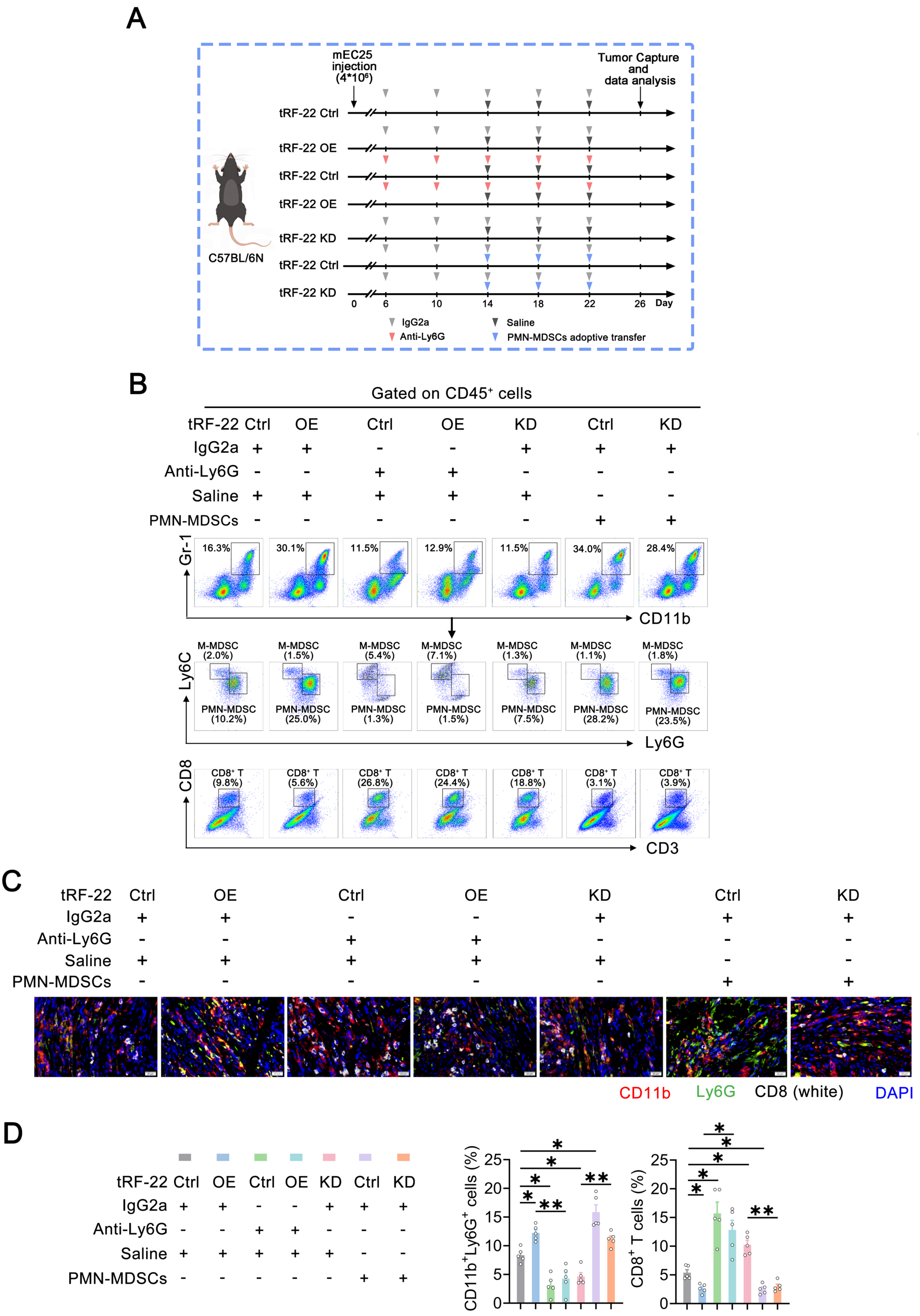
A
mEC25 injection (4*10⁶)
Tumor Capture and data analysis
tRF-22 Ctrl
tRF-22 OE
tRF-22 KD
C57BL/6N
Day
IgG2a
Anti-Ly6G
Saline
PMN-MDSCs adoptive transfer
B
Gated on CD45⁺ cells
IgG2a
Anti-Ly6G
Saline
PMN-MDSCs
Gr-1
CD11b
Ly6C
Ly6G
M-MDSC
PMN-MDSC
CD8
CD3
CD8⁺ T
C
CD11b
Ly6G
CD8 (white)
DAPI
D
CD11b⁺Ly6G⁺ cells (%)
CD8⁺ T cells (%)

Supplementary Figure S8. PMN-MDSCs are the key mediators for tumor-promoting function of *tRF-22*, related to Figure 2.

(***A***) Schematic diagram of *tRF-22* overexpression or silence, combined with anti-Ly6G or PMN-MDSCs adoptive transfer applied in ESCC mouse models (n = 5 per group). (***B***) The representative pictures of PMN-MDSCs and $CD8^+$ T cells by flow cytometry (n = 5 per group). (***C*** and ***D***) The abundance of $CD11b^+Ly6G^+$ PMN-MDSCs and $CD8^+$ T cells within these tumors. Representative mIHC staining images (***C***) and quantification (***D***). Data presented as mean ± SEM. *, $P < 0.05$; **, $P < 0.01$ by Brown-Forsythe ANOVA with Dunnett's T3 multiple comparison test. (***C***) Scale bars, 20 μm.

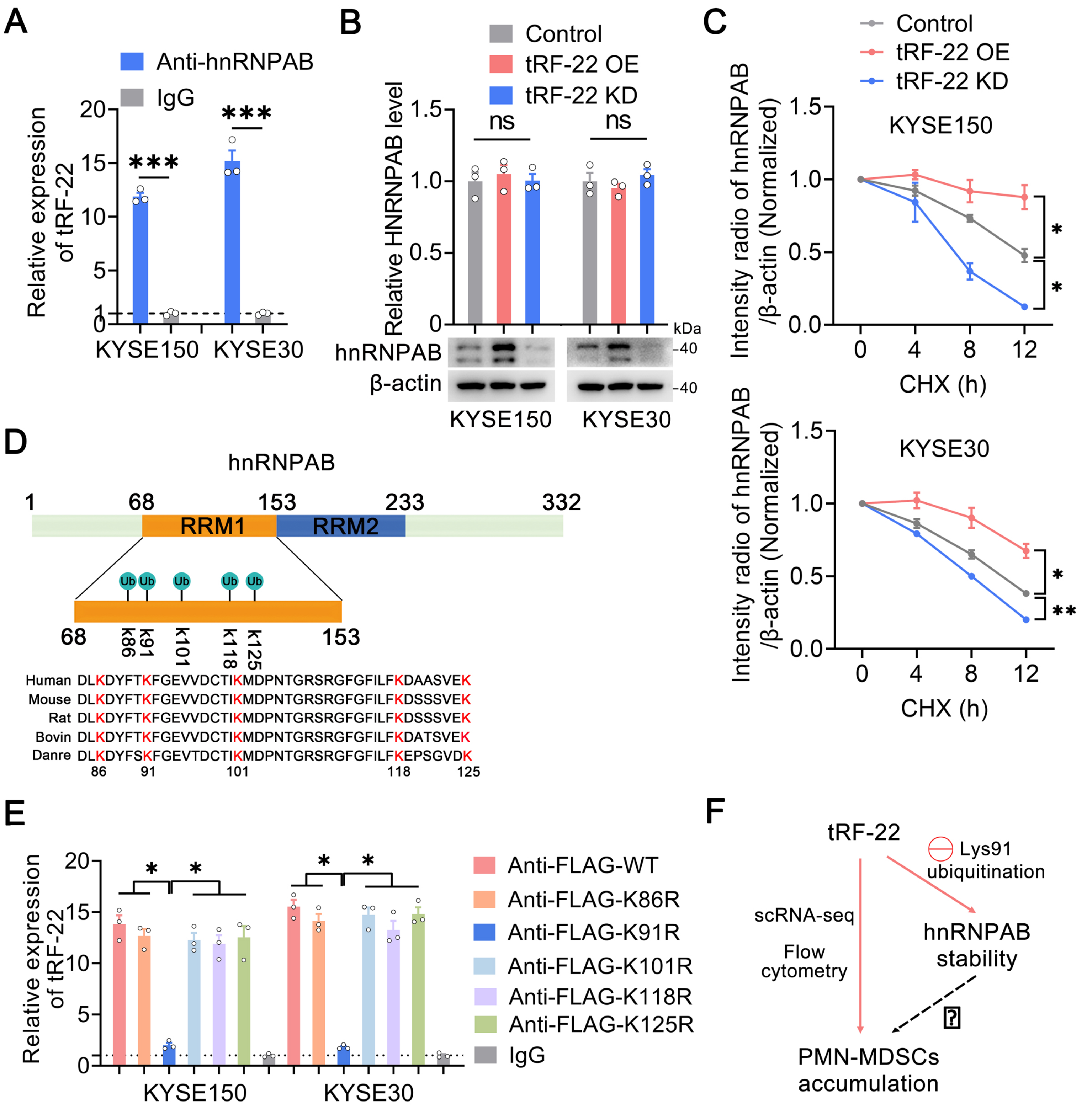
A
Anti-hnRNPAB
IgG
Relative expression of tRF-22
***
***
KYSE150
KYSE30
B
Control
tRF-22 OE
tRF-22 KD
Relative HNRNPAB level
ns
ns
kDa
hnRNPAB
β-actin
-40
-40
KYSE150
KYSE30
C
Control
tRF-22 OE
tRF-22 KD
Intensity radio of hnRNPAB /β-actin (Normalized)
KYSE150
KYSE30
CHX (h)
*
**
D
hnRNPAB
1
68
153
233
332
RRM1
RRM2
Ub
k86
k91
k101
k118
k125
Human DLKDYFTKFGEVVDCTIKMDPNTGRSRGFGFILFKDAASVEK
Mouse DLKDYFTKFGEVVDCTIKMDPNTGRSRGFGFILFKDSSSVEK
Rat DLKDYFTKFGEVVDCTIKMDPNTGRSRGFGFILFKDSSSVEK
Bovin DLKDYFTKFGEVVDCTIKMDPNTGRSRGFGFILFKDATSVEK
Danre DLKDYFSKFGEVTDCTIKMDPNTGRSRGFGFILFKEPSGVDK
86 91 101 118 125
E
Relative expression of tRF-22
Anti-FLAG-WT
Anti-FLAG-K86R
Anti-FLAG-K91R
Anti-FLAG-K101R
Anti-FLAG-K118R
Anti-FLAG-K125R
IgG
KYSE150
KYSE30
F
tRF-22
Lys91 ubiquitination
scRNA-seq
Flow cytometry
hnRNPAB stability
PMN-MDSCs accumulation

Supplementary Figure S9. ***tRF-22*** **interacts with hnRNPAB at Lys91, related to Figure 3.** (***A***) Association of hnRNPAB with *tRF-22* in ESCC cells determined by RIP assays followed by RT-qPCR. RT-qPCR data represent enrichment (mean ± SEM) relative to input. IgG was used as a negative control. ***, *P* < 0.001 by t test. (***B***) *HNRNPAB* mRNA and hnRNPAB protein levels upon *tRF-22* overexpression or silence. RT-qPCR data indicate the mean ± SEM. (***C***) ImageJ software was used to quantify hnRNPAB protein levels and β-actin was used for normalization. (***D***) Schematic of ubiquitination sites in the RRM1 domain of hnRNPAB. (***E***) RIP assays with anti-FLAG antibody showed the interaction of FLAG-tagged hnRNPAB mutants with *tRF-22*. Data represent enrichment (mean ± SEM) relative to input. IgG was used as a negative control. (***F***) A simplified view of the role of hnRNPAB in the *tRF*-22–mediated enhanced PMN-MDSCs accumulation. (***B, C, E***) *, *P* < 0.05; **, *P* < 0.01 and ns, not significant by Brown-Forsythe ANOVA with Dunnett's T3 multiple comparison test. All experiments were performed at least three independent times.

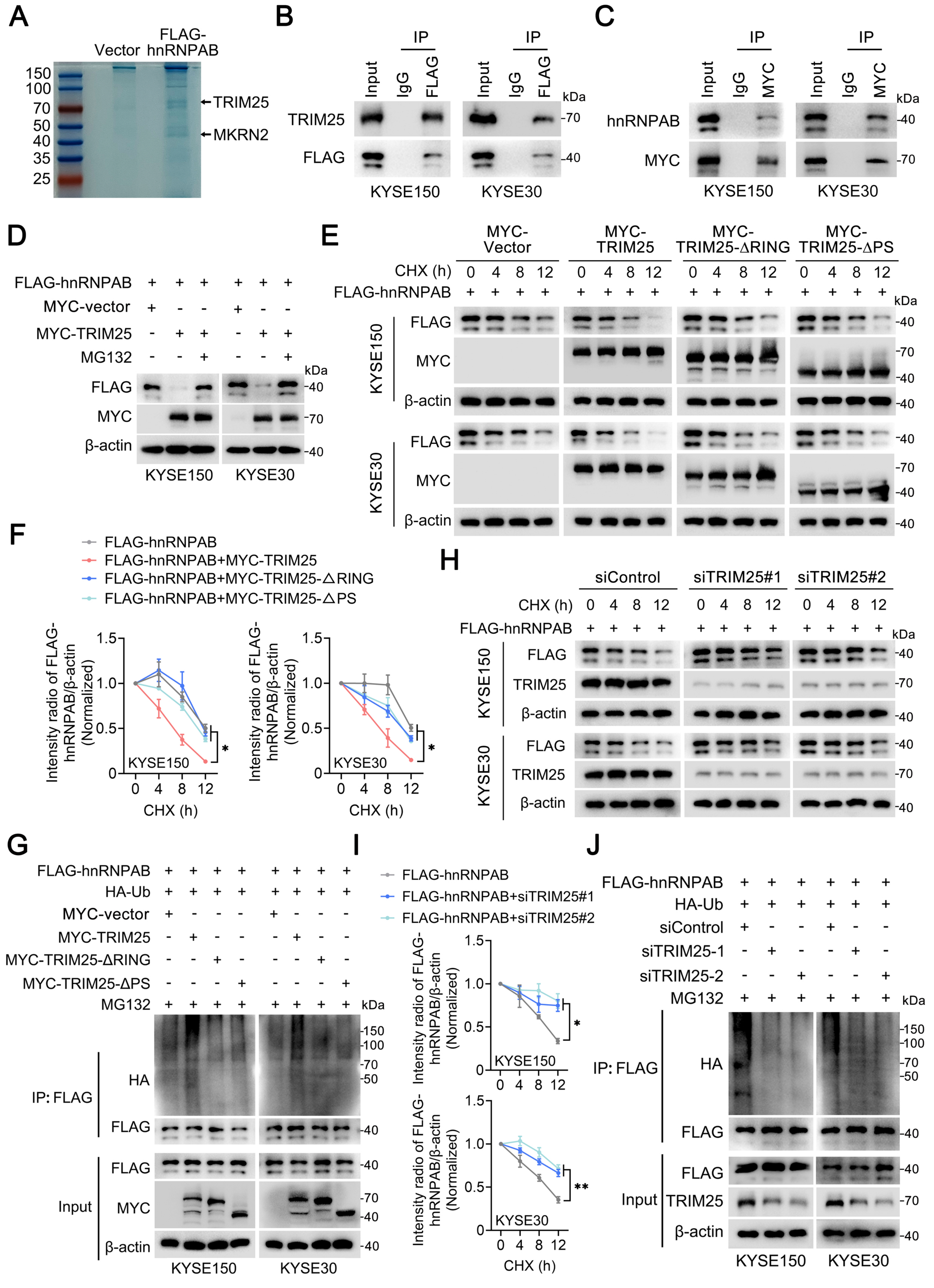
A
Vector
FLAG-hnRNPAB
TRIM25
MKRN2
B
IP
Input IgG FLAG
TRIM25
FLAG
KYSE150
KYSE30
kDa
C
IP
Input IgG MYC
hnRNPAB
MYC
KYSE150
KYSE30
D
FLAG-hnRNPAB
MYC-vector
MYC-TRIM25
MG132
FLAG
MYC
β-actin
KYSE150
KYSE30
E
MYC-Vector
MYC-TRIM25
MYC-TRIM25-ΔRING
MYC-TRIM25-ΔPS
CHX (h)
FLAG-hnRNPAB
KYSE150
KYSE30
FLAG
MYC
β-actin
F
FLAG-hnRNPAB
FLAG-hnRNPAB+MYC-TRIM25
FLAG-hnRNPAB+MYC-TRIM25-ΔRING
FLAG-hnRNPAB+MYC-TRIM25-ΔPS
Intensity radio of FLAG-hnRNPAB/β-actin (Normalized)
KYSE150
KYSE30
CHX (h)
H
siControl
siTRIM25#1
siTRIM25#2
CHX (h)
FLAG-hnRNPAB
FLAG
TRIM25
β-actin
KYSE150
KYSE30
G
FLAG-hnRNPAB
HA-Ub
MYC-vector
MYC-TRIM25
MYC-TRIM25-ΔRING
MYC-TRIM25-ΔPS
MG132
IP: FLAG
HA
FLAG
Input
MYC
β-actin
KYSE150
KYSE30
I
FLAG-hnRNPAB
FLAG-hnRNPAB+siTRIM25#1
FLAG-hnRNPAB+siTRIM25#2
Intensity radio of FLAG-hnRNPAB/β-actin (Normalized)
KYSE150
KYSE30
CHX (h)
J
FLAG-hnRNPAB
HA-Ub
siControl
siTRIM25-1
siTRIM25-2
MG132
IP: FLAG
HA
FLAG
Input
TRIM25
β-actin
KYSE150
KYSE30

Supplementary Figure S10. TRIM25 is the E3 ubiquitin ligase for the ubiquitination and degradation of hnRNPAB, related to Figure 4.

(***A***) Lysates from 293T cells were immunoprecipitated with anti-FLAG agarose and subjected to SDS-PAGE and coomassie staining. (***B*** and ***C***) Reciprocal immunoprecipitation assays showed the interaction of hnRNPAB with TRIM25 and the representative immunoblots were shown. (***D***) ESCC cells co-transfected with the indicated plasmids for 48 h and incubated with MG132 (10 μM) for 4 h, followed by western blotting and the representative immunoblots were shown. (***E***) ESCC cells co-transfected with the indicated plasmids were incubated with CHX for the indicated periods and then analyzed by western blotting and the representative immunoblots were shown. (***F***) Quantitation of FLAG-hnRNPAB levels and β-actin was used for normalization based on the western blotting results shown in (***E***). (***G***) ESCC cells were co-transfected with the indicated plasmids for 48 h and then subjected to immunoprecipitation using anti-FLAG antibody followed by western blotting and the representative immunoblots were shown. (***H***) ESCC cells co-transfected with the indicated plasmids or siRNAs were incubated with CHX for the indicated periods and then analyzed by western blotting, shown are the representative immunoblots. (***I***) Quantitation of FLAG-hnRNPAB levels and β-actin was used for normalization based on the western blotting results shown in (***H***). (***J***) ESCC cells were co-transfected with the indicated plasmids or siRNAs for 48 h and then subjected to immunoprecipitation using anti-FLAG antibody followed by western blotting. The representative immunoblots were shown. (***F, I***) Data presented as mean ± SEM. *, $P < 0.05$, **, $P < 0.01$ by Brown-Forsythe ANOVA with Dunnett's T3 multiple comparison test. All experiments were performed at least three independent times.

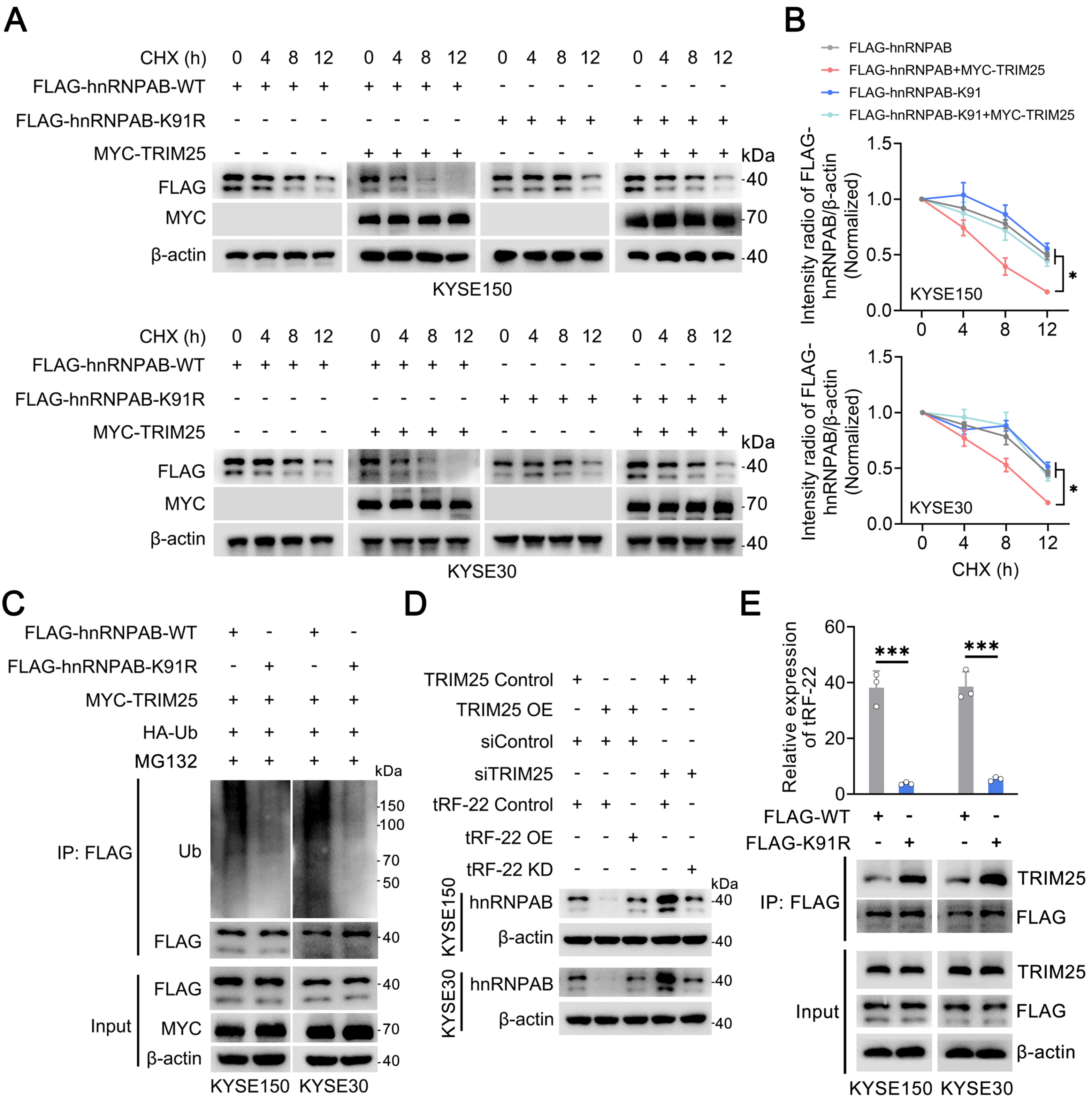
A
CHX (h)
0 4 8 12
FLAG-hnRNPAB-WT
FLAG-hnRNPAB-K91R
MYC-TRIM25
kDa
FLAG
MYC
β-actin
40
70
KYSE150
KYSE30
B
FLAG-hnRNPAB
FLAG-hnRNPAB+MYC-TRIM25
FLAG-hnRNPAB-K91
FLAG-hnRNPAB-K91+MYC-TRIM25
Intensity radio of FLAG-hnRNPAB/β-actin (Normalized)
0.0
0.5
1.0
1.5
CHX (h)
*
C
FLAG-hnRNPAB-WT
FLAG-hnRNPAB-K91R
MYC-TRIM25
HA-Ub
MG132
IP: FLAG
Ub
Input
150
100
50
D
TRIM25 Control
TRIM25 OE
siControl
siTRIM25
tRF-22 Control
tRF-22 OE
tRF-22 KD
hnRNPAB
E
Relative expression of tRF-22
0
20
40
60
***
FLAG-WT
FLAG-K91R
TRIM25

Supplementary Figure S11. *tRF-22* prevents the ubiquitination of hnRNPAB at Lys91 by TRIM25, related to Figure 4.

(***A***) ESCC cells co-transfected with the indicated plasmids were incubated with CHX for the indicated periods and then analyzed by western blotting and the representative immunoblots were shown. (***B***) Quantitation of FLAG-hnRNPAB levels and β-actin was used for normalization based on the western blotting results shown in (***A***). Data presented as mean ± SEM. *, $P < 0.05$ by Brown-Forsythe ANOVA with Dunnett's T3 multiple comparison test. (***C***) ESCC cells were co-transfected with the indicated plasmids for 48 h and then subjected to immunoprecipitation using anti-FLAG antibody followed by western blotting. The representative immunoblots were shown. (***D***) ESCC cells were co-transfected with the indicated plasmids or siRNAs followed by western blotting for quantifying hnRNPAB levels. The representative immunoblots were shown. (***E***) ESCC cells were co-transfected with the indicated plasmids and subjected to RNA or protein immunoprecipitation using anti-FLAG antibody followed by RT-qPCR (*upper panel*) or western blotting (*lower panel*). RT-qPCR data represent enrichment (mean ± SEM) relative to input. IgG was used as a negative control. ***, $P < 0.001$ by t test. All experiments were performed at least three independent times.

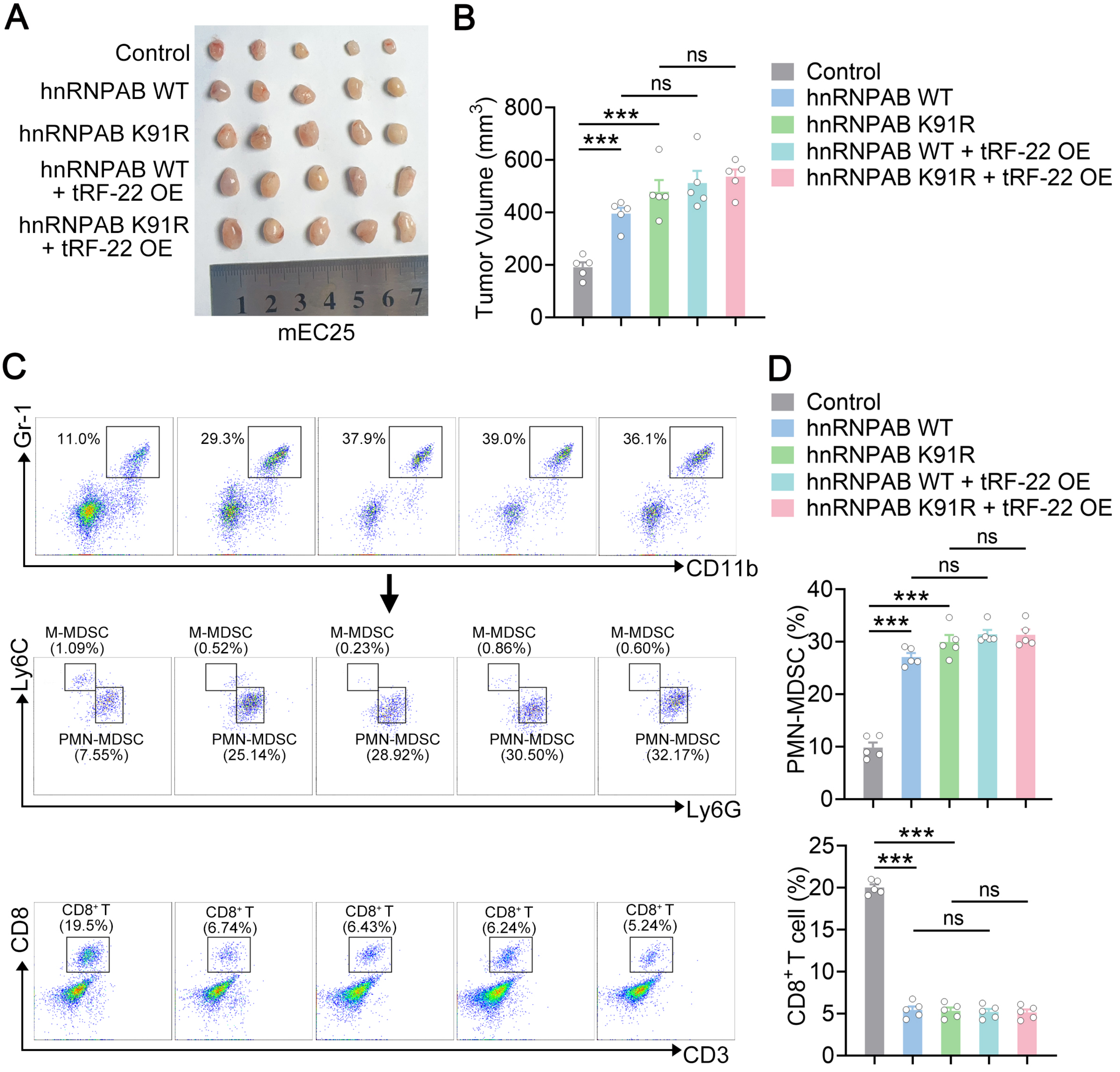

A
Control
hnRNPAB WT
hnRNPAB K91R
hnRNPAB WT + tRF-22 OE
hnRNPAB K91R + tRF-22 OE
mEC25
B
Tumor Volume (mm3)
***
***
ns
ns
C
Gr-1
CD11b
11.0%
29.3%
37.9%
39.0%
36.1%
Ly6C
Ly6G
M-MDSC (1.09%)
M-MDSC (0.52%)
M-MDSC (0.23%)
M-MDSC (0.86%)
M-MDSC (0.60%)
PMN-MDSC (7.55%)
PMN-MDSC (25.14%)
PMN-MDSC (28.92%)
PMN-MDSC (30.50%)
PMN-MDSC (32.17%)
CD8
CD3
CD8+ T (19.5%)
CD8+ T (6.74%)
CD8+ T (6.43%)
CD8+ T (6.24%)
CD8+ T (5.24%)
D
PMN-MDSC (%)
CD8+ T cell (%)

Supplementary Figure S12. K91 hnRNPAB mediates the concurrent interaction of *tRF-22* and TRIM25 *in vivo*, related to Figure 3 and 4.

(***A*** and ***B***) Image (***A***) and quantification (***B***) of tumors receiving treatments as mentioned (n = 5 per group). (***C***) The representative pictures of $CD11b^+Gr\text{-}1^+Ly6G^+$ PMN-MDSCs and $CD3^+CD8^+$ T cells in these tumors by flow cytometry. (***D***) The proportions of PMN-MDSCs (*upper panel*) and $CD8^+$ T cells (*lower panel*) in these tumors by flow cytometry. (***B*** and ***D***) Data presented as mean ± SEM. ***, $P < 0.0001$ and ns, not significant by Brown-Forsythe ANOVA with Dunnett's T3 multiple comparison test.

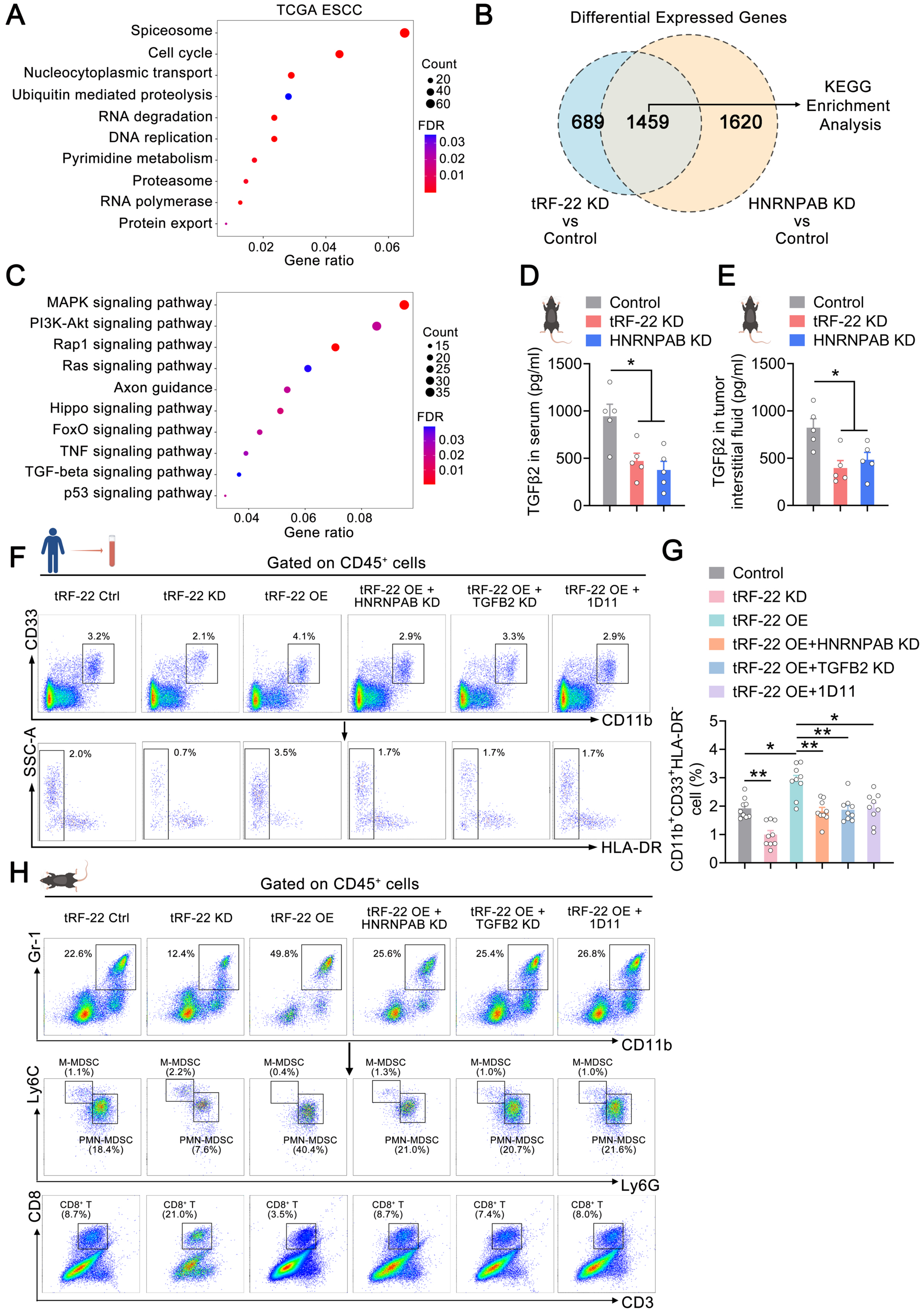

A
TCGA ESCC
Spiceosome
Cell cycle
Nucleocytoplasmic transport
Ubiquitin mediated proteolysis
RNA degradation
DNA replication
Pyrimidine metabolism
Proteasome
RNA polymerase
Protein export
Gene ratio
Count
FDR
B
Differential Expressed Genes
689
1459
1620
KEGG Enrichment Analysis
tRF-22 KD vs Control
HNRNPAB KD vs Control
C
MAPK signaling pathway
PI3K-Akt signaling pathway
Rap1 signaling pathway
Ras signaling pathway
Axon guidance
Hippo signaling pathway
FoxO signaling pathway
TNF signaling pathway
TGF-beta signaling pathway
p53 signaling pathway
Gene ratio
D
Control
tRF-22 KD
HNRNPAB KD
TGFβ2 in serum (pg/ml)
E
TGFβ2 in tumor interstitial fluid (pg/ml)
F
Gated on CD45⁺ cells
tRF-22 Ctrl
tRF-22 KD
tRF-22 OE
tRF-22 OE + HNRNPAB KD
tRF-22 OE + TGFB2 KD
tRF-22 OE + 1D11
CD33
CD11b
SSC-A
HLA-DR
G
Control
tRF-22 KD
tRF-22 OE
tRF-22 OE+HNRNPAB KD
tRF-22 OE+TGFB2 KD
tRF-22 OE+1D11
CD11b⁺CD33⁺HLA-DR⁻ cell (%)
H
Gated on CD45⁺ cells
Gr-1
CD11b
Ly6C
Ly6G
M-MDSC
PMN-MDSC
CD8⁺ T
CD8
CD3

Supplementary Figure S13. PMN-MDSCs accumulation is regulated by *tRF-22* and hnRNPAB-mediated transcription activation of *TGFB2*, related to Figure 5 and Figure 6. (***A***) KEGG enrichment analyses of TCGA ESCC RNA-seq data display pathways associated with *HNRNPAB*. (***B*** and ***C***) Schematic diagram of KEGG enrichment analyses of differential expressed genes from RNA-seq of Control (co-transfected with a negative control for *tRF-22* and siControl for *HNRNPAB*), *tRF-22* or *HNRNPAB*–silenced groups (***B***) and enriched pathways (***C***) (n = 3). (***D*** and ***E***) ELISA analyses of TGFβ2 in serum (***D***) and tumor interstitial fluid (***E***) of tumors obtained from C57BL/6N mice inoculated with mEC25 cells with *tRF-22* or *HNRNPAB* silence (n = 5). (***F*** and ***G***) Flow cytometry dot plot (***F***) and percentage (***G***) of $CD11b^+CD33^+HLA\text{-}DR^-$ cell populations in healthy donor PBMCs cultured in indicated conditional KYSE150 cell culture medium for 5 days (n = 9). (***H***) The representative pictures of $CD11b^+Gr\text{-}1^+Ly6G^+$ PMN-MDSCs and $CD3^+CD8^+$ T cells in tumors by flow cytometry, related to Figure 5I. (***D***, ***E, G***) Data presented as mean ± SEM. *, P < 0.05; **, P < 0.01 by Brown-Forsythe ANOVA with Dunnett's T3 multiple comparison test.

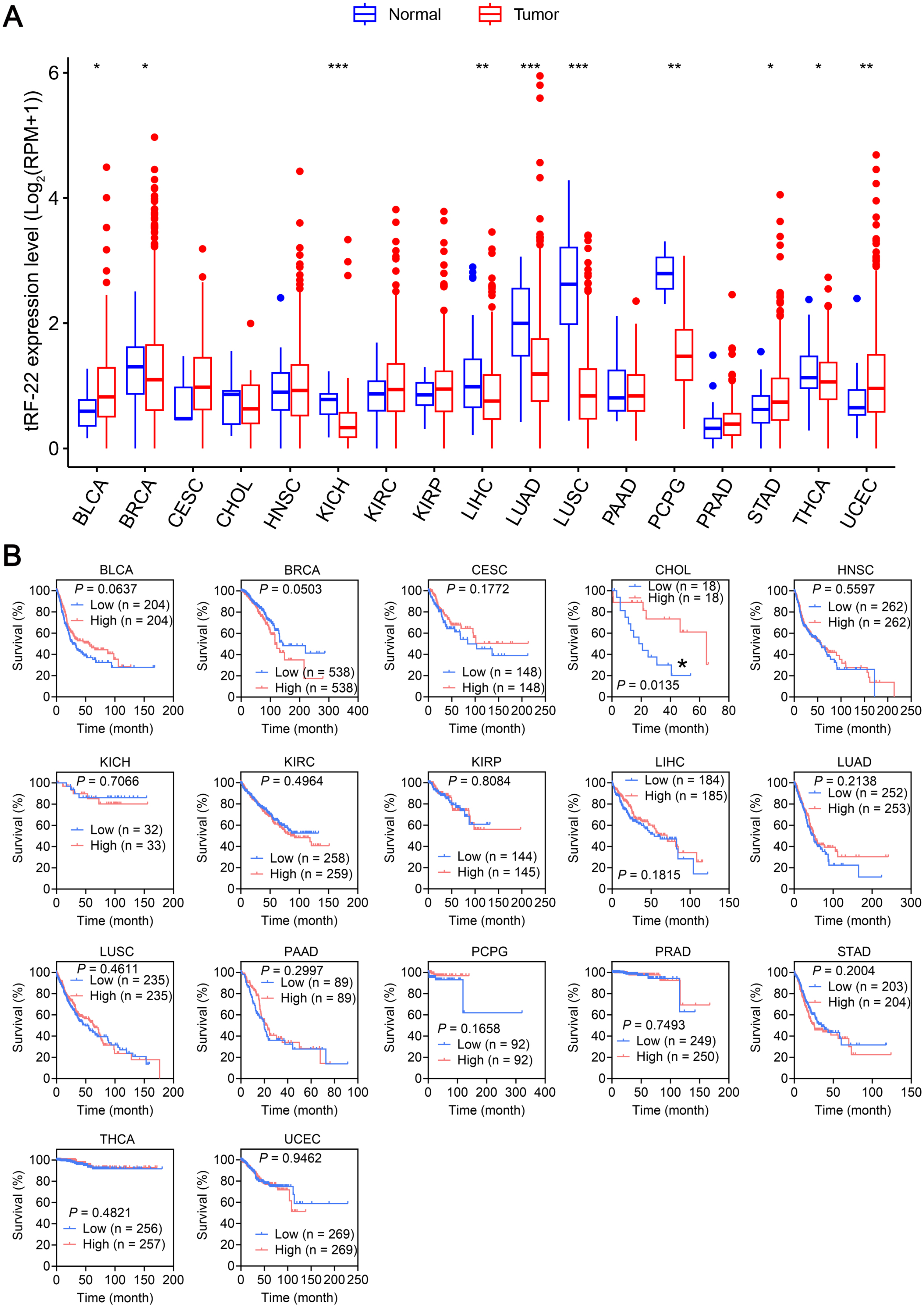
A
Normal
Tumor
tRF-22 expression level (Log2(RPM+1))
0
2
4
6
*
*
***
**
***
***
**
*
*
**
BLCA
BRCA
CESC
CHOL
HNSC
KICH
KIRC
KIRP
LIHC
LUAD
LUSC
PAAD
PCPG
PRAD
STAD
THCA
UCEC
B
BLCA
P = 0.0637
Low (n = 204)
High (n = 204)
BRCA
P = 0.0503
Low (n = 538)
High (n = 538)
CESC
P = 0.1772
Low (n = 148)
High (n = 148)
CHOL
Low (n = 18)
High (n = 18)
*
P = 0.0135
HNSC
P = 0.5597
Low (n = 262)
High (n = 262)
KICH
P = 0.7066
Low (n = 32)
High (n = 33)
KIRC
P = 0.4964
Low (n = 258)
High (n = 259)
KIRP
P = 0.8084
Low (n = 144)
High (n = 145)
LIHC
Low (n = 184)
High (n = 185)
P = 0.1815
LUAD
P = 0.2138
Low (n = 252)
High (n = 253)
LUSC
P = 0.4611
Low (n = 235)
High (n = 235)
PAAD
P = 0.2997
Low (n = 89)
High (n = 89)
PCPG
P = 0.1658
Low (n = 92)
High (n = 92)
PRAD
P = 0.7493
Low (n = 249)
High (n = 250)
STAD
P = 0.2004
Low (n = 203)
High (n = 204)
THCA
P = 0.4821
Low (n = 256)
High (n = 257)
UCEC
P = 0.9462
Low (n = 269)
High (n = 269)
Survival (%)
Time (month)

Supplementary Figure S14. *tRF-22* expression and correlations between its levels in tumors and survival time in patients with different cancer types in the TCGA database, related to Figure 5.

(***A***) Shown are *tRF-22* levels of tumor tissues and their adjacent normal tissues for 17 cancer types in the TCGA database. *, $P < 0.05$, **, $P < 0.01$ and ***, $P < 0.0001$ by Wilcoxon test. (***B***) Shown are Kaplan-Meier survival curves with log-rank P values for 17 cancer types in TCGA database. *, $P < 0.05$.

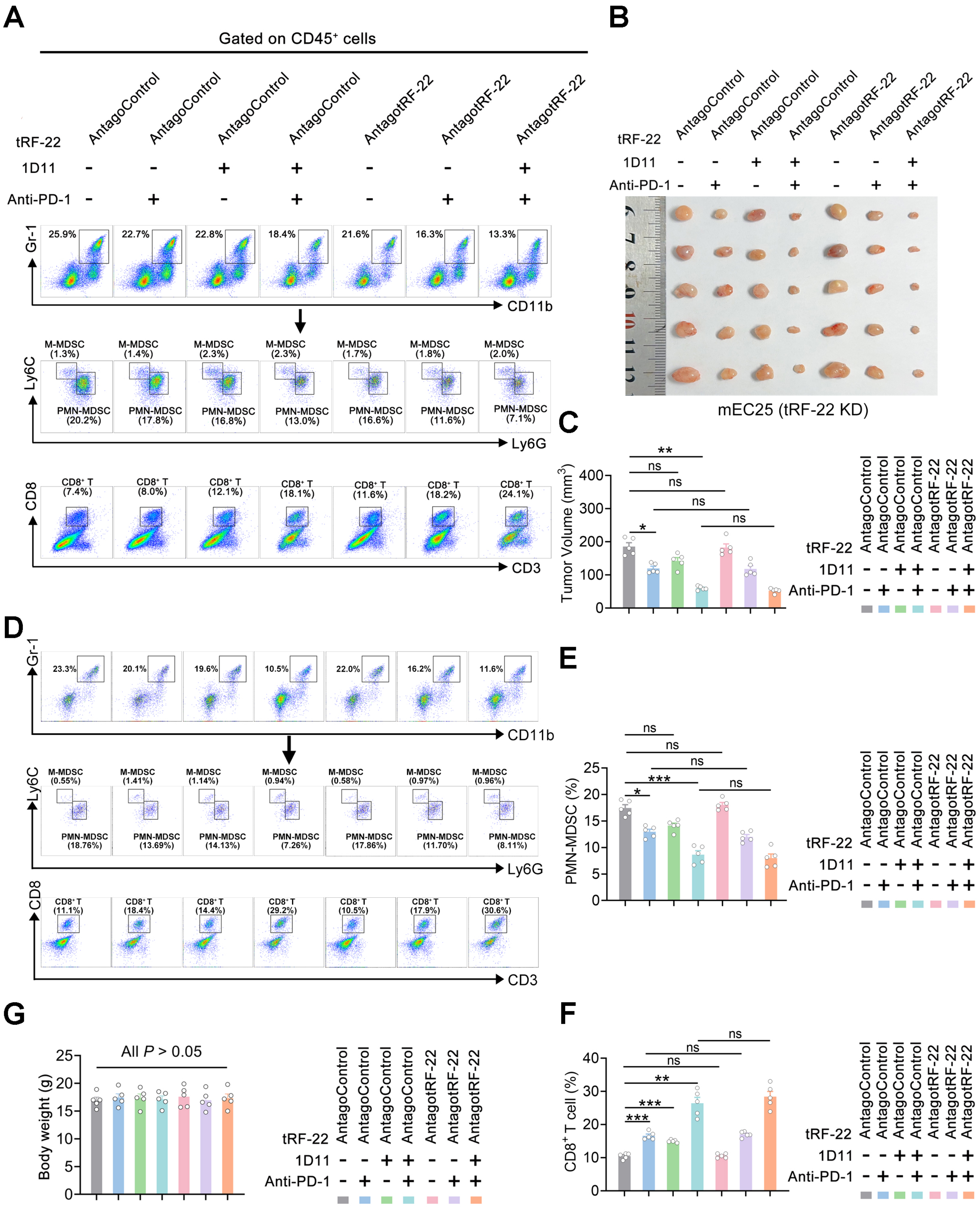
A
Gated on CD45⁺ cells
tRF-22
AntagoControl
AntagoControl
AntagoControl
AntagoControl
AntagotRF-22
AntagotRF-22
AntagotRF-22
1D11 - - + + - - +
Anti-PD-1 - + - + - + +
25.9%
22.7%
22.8%
18.4%
21.6%
16.3%
13.3%
Gr-1
CD11b
M-MDSC (1.3%)
M-MDSC (1.4%)
M-MDSC (2.3%)
M-MDSC (2.3%)
M-MDSC (1.7%)
M-MDSC (1.8%)
M-MDSC (2.0%)
PMN-MDSC (20.2%)
PMN-MDSC (17.8%)
PMN-MDSC (16.8%)
PMN-MDSC (13.0%)
PMN-MDSC (16.6%)
PMN-MDSC (11.6%)
PMN-MDSC (7.1%)
Ly6C
Ly6G
CD8⁺ T (7.4%)
CD8⁺ T (8.0%)
CD8⁺ T (12.1%)
CD8⁺ T (18.1%)
CD8⁺ T (11.6%)
CD8⁺ T (18.2%)
CD8⁺ T (24.1%)
CD8
CD3
B
mEC25 (tRF-22 KD)
C
Tumor Volume (mm³)
ns
*
**
D
23.3%
20.1%
19.6%
10.5%
22.0%
16.2%
11.6%
M-MDSC (0.55%)
M-MDSC (1.41%)
M-MDSC (1.14%)
M-MDSC (0.94%)
M-MDSC (0.58%)
M-MDSC (0.97%)
M-MDSC (0.96%)
PMN-MDSC (18.76%)
PMN-MDSC (13.69%)
PMN-MDSC (14.13%)
PMN-MDSC (7.26%)
PMN-MDSC (17.86%)
PMN-MDSC (11.70%)
PMN-MDSC (8.11%)
CD8⁺ T (11.1%)
CD8⁺ T (18.4%)
CD8⁺ T (14.4%)
CD8⁺ T (29.2%)
CD8⁺ T (10.5%)
CD8⁺ T (17.9%)
CD8⁺ T (30.6%)
E
PMN-MDSC (%)
***
F
CD8⁺ T cell (%)
G
Body weight (g)
All P > 0.05

Supplementary Figure S15. *tRF-22* inhibition and TGFβ2 blockade enhance ICB efficacy in ESCC with high *tRF-22* expression, related to Figure 6.

(***A***) The representative pictures of $CD11b^{+}Gr\text{-}1^{+}Ly6G^{+}$ PMN-MDSCs and $CD3^{+}CD8^{+}$ T cells in tumors by flow cytometry, related to Figure 6C. (***B*** and ***C***) Image (***B***) and quantification (***C***) of tumors obtained receiving the same treatment regimen as Figure 6A (n = 5 per group). (***D***) The representative pictures of $CD11b^{+}Gr\text{-}1^{+}Ly6G^{+}$ PMN-MDSCs and $CD3^{+}CD8^{+}$ T cells in these tumors by flow cytometry. (***E-F***) The proportions of PMN-MDSCs (***E***) and $CD8^{+}$ T cells (***F***) in these tumors by flow cytometry. (***G***) Mice body weight was recorded when the treatments finished, related to Figure 6A. Data in (***C, E-G***) are mean ± SEM (n = 5 per group). (***C, E, F, G***) Data presented as mean ± SEM. *, $P < 0.05$; **, $P < 0.01$; ***, $P < 0.001$ and ns, not significant by Brown-Forsythe ANOVA with Dunnett's T3 multiple comparison test.
