# Supplementary material for: tRNA‐Derived Fragment tRF‐22 Promotes Immunosuppression by Inhibiting HnRNPAB Ubiquitination in Esophageal Squamous Cell Carcinoma
Source: Adv Sci (Weinh). 2025 Oct 27;13(1):e05806. doi: 10.1002/advs.202505806 (PMC12767015; doi:10.1002/advs.202505806)
Supplement: Supplementary file 2 — Supporting Information [file ADVS-13-e05806-s002.pdf]

**Table S1. Analysis of the species conservation of *tRNA*$^{GlnCTG/TTG}$/*tRF-22* sequence between human and mouse.**

| Candidate Mature tRNA Sources | tRNA / tRF Sequence |
|---|---|
| >Homo_sapiens_tRNA-Gln-CTG-1-1 (chr6.trna1-GlnCTG)<br>>Homo_sapiens_tRNA-Gln-CTG-1-2 (chr6.trna51-GlnCTG)<br>>Homo_sapiens_tRNA-Gln-CTG-1-3 (chr6.trna102-GlnCTG)<br>>Homo_sapiens_tRNA-Gln-CTG-1-4 (chr15.trna7-GlnCTG)<br>>Homo_sapiens_tRNA-Gln-CTG-1-5 (chr17.trna2-GlnCTG)<br>>Mus_musculus_tRNA-Gln-CTG-2-1 (chr9.trna550-GlnCTG)<br>>Mus_musculus_tRNA-Gln-CTG-2-2 (chr11.trna2829-GlnCTG)<br>>Mus_musculus_tRNA-Gln-CTG-2-3 (chr13.trna86-GlnCTG)<br>>Mus_musculus_tRNA-Gln-CTG-2-4 (chr13.trna1516-GlnCTG) | GGTTCCATGGTGTAATGGTTAGCACTCTGGACTCTGAATCCAGCGATCCGAGTTCAAATCTCGGTGGAACCT |
| >Homo_sapiens_tRNA-Gln-TTG-3-1 (chr6.trna179-GlnTTG)<br>>Homo_sapiens_tRNA-Gln-TTG-3-2 (chr6.trna178-GlnTTG)<br>>Homo_sapiens_tRNA-Gln-TTG-3-3 (chr6.trna134-GlnTTG)<br>>Mus_musculus_tRNA-Gln-TTG-3-1 (chr13.trna137-GlnTTG)<br>>Mus_musculus_tRNA-Gln-TTG-3-2 (chr13.trna138-GlnTTG) | GGCCCCATGGTGTAATGGTTAGCACTCTGGACTTTGAATCCAGCGATCCGAGTTCAAATCTCGGTGGGACCT |

| | |
|---|---|
| >Homo_sapiens_tRNA-Gln-TTG-1-1 (chr17.trna14-GlnTTG)<br>>Mus_musculus_tRNA-Gln-TTG-1-1 (chr11.trna2317-GlnTTG) | GGTCCCATGGTGTAATGGTTAGCACTCTGGACTTTG<u>AATCCAGCGATCCGAGTTCAAA</u>TCT<br>CGGTGGGACCT |

**Table S2. Proteins interacted with *tRF-22* in ESCC cell lines identified by mass spectrometry.**

| No. | Name and official symbol | Abundance (KYSE150) | Abundance (KYSE30) |
|---|---|---|---|
| 1 | Staphylococcal nuclease domain-containing protein 1, SND1 | 2536934 | 10861959 |
| 2 | Filamin A-interacting protein 1-like (Fragment), FILIP1L | 15690950 | 8433112 |
| 3 | Enhancer of rudimentary homolog, ERH | 5359959 | 6191951 |
| 4 | Heterogeneous nuclear ribonucleoprotein A/B, HNRNPAB | 5177691 | 2620050 |
| 5 | RNA-binding motif protein, X chromosome, RBMX | 1487516 | 2491019 |
| 6 | Flap endonuclease 1 (Fragment), FEN1 | 693393 | 1649056 |
| 7 | ATP synthase subunit alpha, mitochondrial, ATP5F1A | 3061986 | 1618098 |
| 8 | Serine-threonine kinase receptor-associated protein, STRAP | 983168 | 1542458 |
| 9 | Ataxin 1, isoform CRA_c, ATXN1 | 3500302 | 1341622 |
| 10 | Protein S100-A8, S100A8 | 461699 | 919930 |
| 11 | 60S ribosomal protein L14, RPL14 | 728809 | 799059 |
| 12 | NAD-dependent malic enzyme, mitochondrial, ME2 | 20688709 | 700060 |
| 13 | CD59 glycoprotein (Fragment), CD59 | 1259516 | 593311 |
| 14 | Heterogeneous nuclear ribonucleoproteins C1/C2, HNRNPC | 560761 | 488099 |
| 15 | Histone H2B type 2-F, H2BC18 | 3323776 | 475916 |
| 16 | 1,4-beta-N-acetylmuramidase C, LYZ | 647871 | 251100 |
| 17 | Signal recognition particle 9 kDa protein, SRP9 | 166926 | 130105 |
| 18 | Drug-sensitive protein 1, YA61 | 500852 | 129359 |

**Table S3. E3-ligases interacted with hnRNPAB identified by mass spectrometry.**

| No. | Name and official symbol | Unique Peptides | Score |
|---|---|---|---|
| 1 | E3 ubiquitin/ISG15 ligase TRIM25, TRIM25 | 2 | 1.74 |
| 2 | Probable E3 ubiquitin-protein ligase makorin-2, MKRN2 | 1 | 1.94 |

**Table S4. Baseline demographic and clinical characteristics of individuals with esophageal squamous cell carcinoma (ESCC) in Figure 1.**

| | Stable (*n* = 76) | Relapsed (*n* = 24) |
|---|---|---|
| Age, mean (SEM[a]) | 64.4 (0.8) | 64.0 (1.8) |
| Sex, *n* (%) | | |
| Male | 61 (80.3) | 19 (79.2) |
| Female | 15 (19.7) | 5 (20.8) |
| Differentiation, *n* (%) | | |
| Well | 7 (9.2) | 0 (0) |
| Moderate | 51 (67.1) | 17 (70.8) |
| Poor | 15 (19.7) | 5 (20.8) |
| Unknown | 3 (3.9) | 2 (8.3) |
| Lymph node metastasis, *n* (%) | | |
| Positive | 34 (44.7) | 19 (79.2) |
| Negative | 42 (55.3) | 5 (20.8) |
| Intravascular tumor thrombus, *n* (%) | | |
| Yes | 17 (22.4) | 8 (33.3) |
| No | 52 (68.4) | 11 (45.8) |
| Unknown | 7 (9.2) | 5 (20.8) |
| Neural invasion, *n* (%) | | |
| Yes | 11 (14.5) | 7 (29.2) |
| No | 58 (76.3) | 12 (50.0) |
| Unknown | 7 (9.2) | 5 (20.8) |
| TNM stage[b], *n* (%) | | |
| I | 13 (17.1) | 0 (0) |
| II | 29 (38.2) | 6 (25.0) |
| III | 33 (43.4) | 17 (70.8) |
| IV | 1 (1.3) | 1 (4.2) |
| Smoking status, *n* (%) | | |
| Ever | 47 (61.8) | 17 (70.8) |
| Never | 25 (32.9) | 7 (29.2) |
| Unknown | 4 (5.3) | 0 (0) |
| Drinking status, *n* (%) | | |
| Ever | 50 (65.8) | 18 (75.0) |
| Never | 21 (27.6) | 5 (20.8) |
| Unknown | 5 (6.6) | 1 (4.2) |
| Treatment, n (%) | | |
| Surgery only | 55 (72.4) | 13 (54.2) |
| Surgery + neo-adjuvant therapy | 3 (3.9) | 0 (0) |
| Surgery + radiotherapy / chemotherapy / Targeted therapy / Immunotherapy | 18 (23.7) | 11 (45.8) |

[a]SEM, standard error of mean.

[b]Tumor TNM staging were reviewed by at least 3 pathologists and defined according to the American Joint Committee on Cancer (AJCC) 8th edition.

**Table S5. Baseline demographic and clinical characteristics of individuals with ESCC treated with ICB in Figure 7.**

| No. | Sex | Age | Progression | Smoking | Drinking |
|---|---|---|---|---|---|
| Patient 1 | Male | 53 | No | No | No |
| Patient 2 | Male | 65 | No | No | No |
| Patient 3 | Female | 65 | Yes | No | No |
| Patient 4 | Male | 71 | Yes | No | No |
| Patient 5 | Male | 62 | No | Yes | Yes |
| Patient 6 | Male | 55 | Yes | Yes | Yes |
| Patient 7 | Male | 53 | No | No | No |
| Patient 8 | Male | 62 | Yes | No | No |
| Patient 9 | Male | 56 | Yes | Yes | Yes |
| Patient 10 | Male | 59 | No | Yes | Yes |
| Patient 11 | Male | 68 | No | Yes | No |
| Patient 12 | Male | 58 | No | No | No |
| Patient 13 | Male | 69 | No | No | Yes |
| Patient 14 | Male | 57 | Yes | Yes | Yes |
| Patient 15 | Male | 63 | Yes | Yes | Yes |
| Patient 16 | Male | 59 | Yes | Yes | Yes |
| Patient 17 | Male | 49 | No | Yes | Yes |
| Patient 18 | Male | 77 | Yes | No | No |
| Patient 19 | Male | 62 | No | Yes | Yes |
| Patient 20 | Male | 63 | No | No | No |
| Patient 21 | Male | 70 | No | Yes | Yes |
| Patient 22 | Male | 57 | No | Yes | Yes |
| Patient 23 | Male | 56 | No | No | Yes |

**Table S6. Antibodies used in this study.**

**Flow cytometry**

| **Species** | **Antibody** | **Fluorophore** | **Clone** | **Vendor** |
|---|---|---|---|---|
| Human /Mouse | Live/dead | Viability Dye 780 | - | Biogems |
| Mouse | CD45 | PE/Cyanine7 | 30-F11 | Biolegend |
| Human /Mouse | CD11b | FITC | M1/70 | Biolegend |
| Mouse | CD11c | APC | N418 | Biolegend |
| Mouse | F4/80 | PE | BM8 | Biolegend |
| Mouse | Gr-1 | BV421 | RB6-8C5 | Biolegend |
| Mouse | Ly6G | BV510 | 1A8 | Biolegend |
| Mouse | Ly6C | BV650 | HK1.4 | Biolegend |
| Mouse | CD4 | FITC | GK1.5 | ebioscience |
| Mouse | CD19 | APC | 6D5 | Biolegend |
| Mouse | NK1.1 | PE | PK136 | ebioscience |
| Mouse | CD8 | BV421 | 53-6.7 | Biolegend |
| Mouse | CD3 | BV510 | 145-2C11 | BD |
| Mouse | FOXP3 | APC | FJK-16s | ebioscience |
| Mouse | CD25 | PE | PC61.5 | ebioscience |
| Human | CD33 | APC | WM-53 | ebioscience |
| Human | HLA-DR | PE/Cyanine7 | LN3 | ebioscience |

**Western blot and Northern blot**

| Species | Immunogen | Source | Dilution | Vendor |
|---|---|---|---|---|
| Human | hnRNPAB | Mouse | 1/200 | Santa Cruz |
| Human | FILIP1L | Rabbit | 1/1000 | SAB |
| Human | ERH | Rabbit | 1/1500 | Immunoway |
| Human | SND1 | Rabbit | 1/1000 | HUABIO |
| Human | RBMX | Rabbit | 1/1000 | HUABIO |
| Human | ATP5F1A | Rabbit | 1/10000 | Proteintech |
| Human | ATXN1 | Mouse | 1/1000 | HUABIO |
| Human | TRIM25 | Mouse | 1/1000 | Proteintech |
| Human | MKRN2 | Rabbit | 1/8000 | Proteintech |
| Human | Ubiquitin | Rabbit | 1/1000 | Proteintech |
| Human | FLAG | Mouse | 1/1000 | Sigma |
| Human | MYC | Rabbit | 1/4000 | Proteintech |
| Human | HA | Mouse | 1/2000 | Abways |
| Human | β-actin | Mouse | 1/2000 | Servicebio |
| Human /Mouse | Digoxigenin | Sheep | 1/5000 | Roche |

**IHC and IF staining**

| Species | Immunogen | Source | Dilution | Vendor |
|---|---|---|---|---|
| Human /Mouse | CD11b | Rabbit | 1/200 | Bioss |
| Human | CD15 | Rabbit | 1/200 | Immunoway |

| | | | | |
|---|---|---|---|---|
| Human /Mouse | CD8a | Rabbit | 1/1000 | Proteintech |
| Human | hnRNPAB | Mouse | 1/200 | Santa Cruz |
| Human | TGFB2 | Rabbit | 1/200 | Bioss |
| Mouse | Ly6G | Rabbit | 1/200 | HUABIO |

**Table S7. Primers and probes used in this study.**

| Species | RNA pulldown probes (3'-biotin) | Sequence (5' → 3') |
|---|---|---|
| Human | non-targeting oligo | UUGUACUACACAAAAGUACUG |
| Human | *tRF-22-DRFU8U76F* sense | AAUCCAGCGAUCCGAGUUCAAA |
| Human | *tRF-22-DRFU8U76F* antisense | UUUGAACUCGGAUCGCUGGAUU |

| Species | qRT-PCR primers for tRFs | Sequence (5' → 3') Forward | Reverse |
|---|---|---|---|
| Human/Mouse | *tRF-22-DRFU8U76F* | ACACGAATCCAGCGATCCG | TATCCTTCTTCACGACTCCTTCAC |
| Human/Mouse | *U6* | CAGCACATATACTAAAATTGGAACG | ACGAATTTGCGTGTCATCC |

| Species | Gene Symbol | Sequence (5' → 3') Forward | Reverse |
|---|---|---|---|
| Human | *HNRNPAB* | TTTGGCGAGTTTGGGGAGATT | GCCATACTGCTGCTGCTGATAGAC |
| Mouse | *hnrnpab* | AGCCCAAAGAGGTGTATCAGC | TGTACTACCCTGACCTCCACC |
| Human | *TGFB2* | TCACCAGTCCCCCAGAAGAC | AGTACTCTTCGTCGCTCCTCT |
| Mouse | *tgfb2* | AGAGCTCGAGGCGAGATTTG | GATGTGGGGTCTTCCCACTG |
| Human | *THBS1* | TGCTCCAATGCCACAGTTCC | CTGCTGAATTCCATTGCCACA |
| Human | *CCND1* | GTGCATCTACACCGACAACTCC | GTTCCACTTGAGCTTGTTCACC |
| Human | *IL-1B* | GACCACCACTACAGCAAGGG | AGGGAAAGAAGGTGCTCAGGT |
| Human | *MAP2K3* | AACCAGAAGGGCTACAATGTCAAG | TCGTAAGGGAACCGCAGGAT |
| Human | *ETS1* | GGAGATGGCTGGGAATTCAAACTT | CCGCTGTCTTGTGGATGATGTT |

| | | | |
|---|---|---|---|
| Human | *EPHA2* | CACCACAACATCATCCGCCTAG | GCTGAACTCGCCATCCTTCTC |
| Human | *BCL2L1* | CCTGAATGACCACCTAGAGCC | GAAGAGTGAGCCCAGCAGAAC |
| Human | *PLXNA2* | TCCTCATCATCGTCATCATCGT | CAGGTCACTGGTCAACTCATTG |
| Human | *LAMB3* | GACGCACACGGCTCCTAATC | TCGCTGACCTCCTGGATAGTG |
| Human | *PLK2* | AGGCTAGAACCCTTGGAACAC | TCTGATTCACAGCCATGTCCT |
| Human/Mouse | *ACTB* | GACAGGATGCAGAAGGAGAT | GAGGCCAGGATGGAGC |

| **Species** | **CUT & RUN** | **Sequence (5' → 3')** | |
|---|---|---|---|
| | | **Forward** | **Reverse** |
| Human | *TGFB2* (Site 1) | GGGAGGACCTAACTGGATGAAACT | CCAGCAGGTTCCTTTCCTTGATAC |
| Human | *TGFB2* (Site 2) | CAGCTTCCTGGATTAATGCTATGG | CTGCCTAAGCCACTTCTTGTATG |
| Human | *TGFB2* (Site 3) | GGTGAAGAAGAAAGCCATACAAGA | CTCTGTTTGTGAACTGCTGGAA |
| Human | *TGFB2* (Site 4) | AGGCTGTGACTGAGCTACACTAAG | GGAGGAAGGAGGTGGAATGTG |

| **Species** | **RNA FISH Probe (5'-CY3)** | **Sequence (5' → 3')** |
|---|---|---|
| Human | *tRF-22-DRFU8U76F* | UUUGAACUCGGAUCGCUGGAUU |

| **Species** | **Northern blot Probe (5'-DIG)** | **Sequence (5' → 3')** |
|---|---|---|
| Human/Mouse | *tRF-22-DRFU8U76F* | UUUGAACUCGGAUCGCUGGAUU |

**Table S8. Sequences of siRNAs used in this study.**

| Species | siRNAs | Sequence (5’ → 3’) |
|---|---|---|
| Human/Mouse | siControl | UUCUCCGAACGUGUCACGUTT |
| Human | HNRNPAB siRNA#1 | GGUAGUACAAACUACGGCATT |
| Human | HNRNPAB siRNA#2 | TGGAAGCAAGTGTGAGATCAATT |
| Mouse | hnrnpab siRNA#1 | CCAUUGAGCUUCCAAUAGATT |
| Mouse | hnrnpab siRNA#2 | GAAGGACCCUGUGAAGAAATT |
| Human | TGFB2 siRNA#1 | GCGGCCUAUUGCUUUAGAATT |
| Human | TGFB2 siRNA#2 | GACCCUACUUCAGAAUUGUTT |
| Mouse | tgfb2 siRNA#1 | GAGGUGAUUUCCAUCUACATT |
| Mouse | tgfb2 siRNA#2 | GCAGCGGAUUGAACUGUAUTT |
| Human | TRIM25 siRNA#1 | AACUGAACCACAAGCUGAUATT |
| Human | TRIM25 siRNA#2 | CTGGUGCCACACUCUCCAUCUTT |
| **Species** | **Inhibitor/antagomir** | **Sequence (5’→ 3’)** |
| Human | *tRF-22-DRFU8U76F* Control | CAGUACUUUUGUGUAGUACAA |
| Human | *tRF-22-DRFU8U76F* inhibitor | UUUGAACUCGGAUCGCUGGAUU |
| Mouse | antagoControl | CAGUACUUUUGUGUAGUACAA |
| Mouse | antago*tRF-22-DRFU8U76F* | UUUGAACUCGGAUCGCUGGAUU |
